# Photo-redox reactivity of titanium-oxo clusters: mechanistic insight into a two-electron intramolecular process, and structural characterisation of mixed-valent Ti(iii)/Ti(iv) products†
†Electronic supplementary information (ESI) available: NMR, ESI-MS and IR spectra for complexes **1–8**, crystallography details, *in situ* NMR, EPR and UV/vis spectroscopy data. CCDC 1902111–1902116 and 1912033–1912035. For ESI and crystallographic data in CIF or other electronic format see DOI: 10.1039/c9sc01241a


**DOI:** 10.1039/c9sc01241a

**Published:** 2019-06-07

**Authors:** Tobias Krämer, Floriana Tuna, Sebastian. D. Pike

**Affiliations:** a Department of Chemistry , University of Cambridge , Lensfield Road , CB2 1EW , UK . Email: sp842@cam.ac.uk; b Department of Chemistry , Maynooth University , Maynooth , Co. Kildare , Ireland; c School of Chemistry and Photon Science Institute , University of Manchester , Oxford Road , Manchester , M13 9PL , UK

## Abstract

Small titanium-oxo-alkoxide clusters, [TiO(OR)(O_2_PR′_2_)]_4_, synthesised by the stoichiometric reaction of Ti(O^i^Pr)_4_, phosphinic acid and water, undergo a photo-redox transformation under long-wave UV light. The photo-reaction generates blue coloured, mixed-valence Ti(iii)/Ti(iv)-oxo clusters alongside acetone and isopropanol by-products. This reactivity indicates the ability for photoactivated charge separation to occur in even the smallest of Ti-oxo clusters. EPR and NMR spectroscopic studies support a photo-redox mechanism that occurs *via* an intramolecular, two-electron pathway, directly relating to current doubling effects observed at TiO_2_ photoanodes in the presence of alcohols. The rate of photo-reaction is solvent dependent, with donor solvents supporting the formation of low coordinate Ti(iii) sites. The nature of the electronic transition is identified by DFT and TDDFT calculations as an oxygen to titanium charge transfer and it is possible to finetune the UV absorption onset observed by changing the phosphinate ligand. A two-electron photo-reduced cluster, [Ti_4_O_4_(O_2_PPh_2_)_6_], forms spontaneously from the photo-reaction and its structure is identified by X-ray crystallography with supporting DFT calculations. These indicate that [Ti_4_O_4_(O_2_PPh_2_)_6_] is high-spin and contains two ferromagnetically coupled electrons delocalised over the Ti_4_ core. [Ti_4_O_4_(O_2_PPh_2_)_6_] undergoes rapid oxidation in air in the solid-state and performs a remarkable single-crystal to single-crystal transformation, to form a stable cluster-superoxide salt.

## Abstract 2

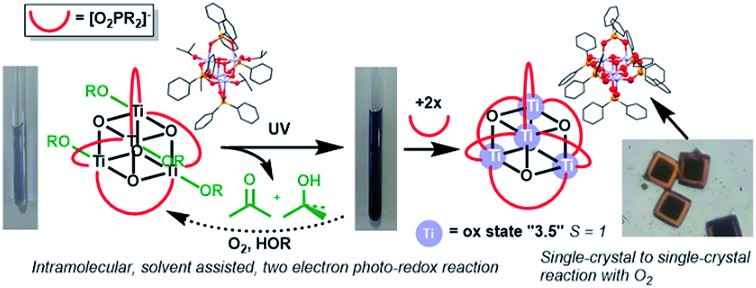
The photo-reactivity of titanium-oxo clusters is investigated, revealing an intramolecular, solvent assisted, two-electron redox process that generates blue-coloured Ti(iii)/Ti(iv) clusters.

## Introduction

TiO_2_ is an important semiconductor material, with applications ranging from sun creams and pigments, to self-cleaning windows, antibacterial surfaces, photovoltaics and photocatalysis.^1^–^7^ Many applications arise from the ability to absorb UV light across the band gap (3.03–3.2 eV), promoting electrons from the predominantly oxygen-based valence band to the titanium (3d)-based conduction band. Atomically precise titanium-oxo clusters, with sizes of up to 52 Ti centres,^8^–^12^ act as molecular relations to TiO_2_ and due to their precise molecular structures offer great opportunities for detailed mechanistic insight. In particular, these systems may act as useful models for dye-sensitised solar cells.^13^–^16^ Other applications of this family of clusters include as photocatalysts, fluorescent labels, vertices within metal organic frameworks (MOFs), or nano-building blocks for hybrid materials,^17^,^18^ and due to their good solubility they may act as single source precursors for thin films.^19^,^20^

**Fig. 1:**
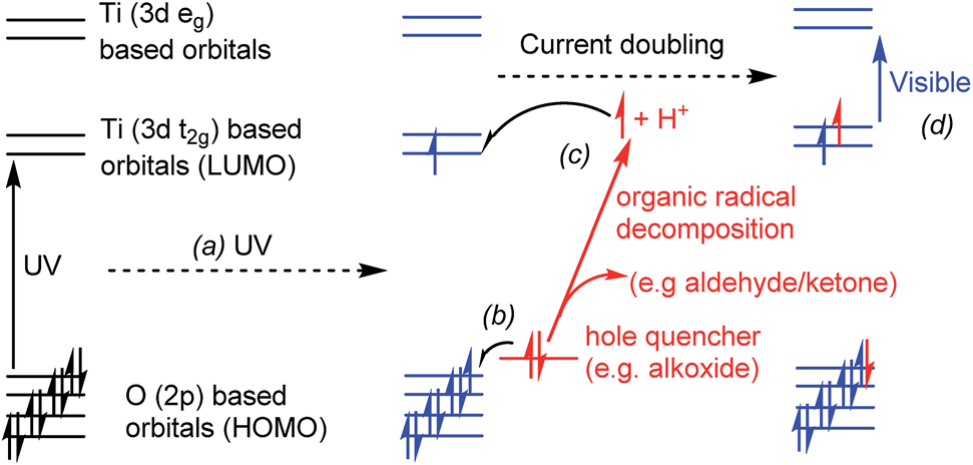
Conceptual diagram showing a photo-excitation process in a titanium-oxo cluster, (a) UV light promotes an electron from oxygen-based orbitals to titanium orbitals (OMCT), (b) hole quencher donates an electron to fill the photogenerated hole and becomes an unstable radical, (c) organic radical may decompose (current doubling effect) releasing a proton and an electron which is passed onto the Ti (3d) orbitals of the cluster. The final product is coloured due to electronic transitions (d) in the visible range (*e.g.* d–d transitions or intervalence charge transfer).

Titanium-oxo clusters are also able to absorb UV light, which promotes an electron into the titanium (3d) orbitals (Fig. 1a). The minimum excitation energy is generally larger in small clusters than that required for bulk TiO_2_.^8^,^21^ These enlarged absorption energies are consistent with quantum confinement effects which are estimated to occur in TiO_2_ particles smaller than ∼2 nm.^22^ Considering this effect from a molecular perspective, the frontier molecular orbitals in Ti-oxo clusters comprise of a combination of atomic orbitals (O 2p orbitals in the HOMO and Ti 3d orbitals in the LUMO). As clusters increase in size towards nanoparticles a combination of a greater number of atomic orbitals increases the spread of available energy states and pushes the HOMO and LUMO closer together, with the orbitals accumulating towards a band structure. However, it should be noted that in molecular systems the supporting ligands,^23^ anion or cation dopants,^24^,^25^ and cluster shape may all affect the charge transfer absorption onset in titanium-oxo clusters, and may alter the lowest energy electronic transition from an oxygen-to-metal charge transfer (OMCT, analogous to the band-gap transition in TiO_2_) into a ligand-to-metal charge transfer (LMCT) in dye substituted clusters.^13^–^16^

If photo-excitation of titanium-oxo clusters is conducted in the presence of a hole quencher (*e.g.* an alcohol or alkoxide ligand) the excited hole can be transferred away from the cluster, resulting in a formally reduced molecule with Ti(iii) sites, which is typically blue in colour (Fig. 1). Further decomposition of an oxidised alcohol (*e.g.* a radical species) can result in the production of a further electron and proton, which we show can be passed back onto the metal-oxo cluster. Such a two-electron photo-redox process is referred to as “current doubling” in the analogous reaction at TiO_2_ photoanodes (Fig. 1).^6^,^26^,^27^

There have been multiple reports of the photo-reduction of titanium-oxo clusters, MOFs, and bulk or nanoscale TiO_2_ under UV light, leading to blue or black coloured materials, with the Ti(iii) sites identified by electron paramagnetic resonance (EPR) spectroscopy. For instance, the titanium based MOF COK-69, (Ti_3_(μ_3_-O)(μ_2_-O)_2_(H_2_O)(1,4-C_6_H_4_(CO_2_)_2_)_3_), may be photo-reduced in ethanol to give a blue solid containing one Ti(iii) site per Ti_3_ vertex.^28^ Isotope studies reveal that this process occurs *via* proton-coupled electron transfer with ethanol as reductant, with an oxo site gaining a proton during the process. Further notable examples are the reversible yellow to dark-purple colour change in dye-sensitised Ti_6_O_6_(O^i^Pr)_6_(9-AC)_6_ (9-AC = 9-anthracenecarboxylate) as electrons are transferred from the dye to Ti upon irradiation,^13^ and the photo-reduction of cluster Ti_6_O_3_(O^i^Pr)_14_(1,2-C_6_H_4_(CO_2_)_2_)_2_ in the solid-state, which resulted in a darkening of the surface of the crystalline material.^29^ Detailed studies have also investigated the photo-reduction of TiO_2_ (or ZnO) nanoparticles in the presence of alcohols describing the ability to store electrons within the conduction band of these semiconductors, which may then undergo onward reactivity *via* proton-coupled electron transfer.^30^–^33^ These coloured photo-reduced titanium-oxo species relate to partially reduced TiO_2_ materials which may be blue or black in colour, and may be employed as photocatalysts which operate using visible light.^34^–^36^

Despite the many reports which discuss photo-reduction, there is a scarcity of structural characterisation of the highly reactive coloured Ti(iii) containing products, which typically react rapidly with air, reducing O_2_ to superoxide radicals.^24^,^37^ There are also only isolated reports of chemically reduced titanium-oxo clusters, such as the blue octahedral cluster Cp_6_Ti_6_O_8_ with formally two Ti(iii) centres, formed *via* reaction of Cp_2_Ti(CO)_2_ with H_2_/CO.^38^

Whilst much success in building Ti-oxo clusters has been achieved by solvothermal synthetic methods, the hydrolysis of Ti-alkoxide units with a stoichiometric quantity of water is an attractive and controlled approach. In this report, small phosphinate supported titanium-oxo-alkoxide clusters are synthesised in this way and their reactivity with UV light examined. The use of phosphinate ligands (R_2_PO_2_^–^) to stabilise the clusters is favourable for several reasons: oxygen donors maintain the relationship with bulk metal oxides; variable R groups allow solubility to be optimised; the ^31^P nucleus allows for *in situ* multinuclear NMR spectroscopy;^39^ and, unlike carboxylates,^19^,^40^ competing esterification reactions between ligand and alkoxide are not observed – allowing excellent stoichiometric control of added moisture. The small clusters allow detailed mechanistic examination of the photo-reaction using spectroscopic methods, revealing that two alkoxide ligands upon the same cluster can be converted into a ketone and a free alcohol by a photo-oxidation process. This process leaves a doubly photo-reduced mixed-valent Ti-oxo cluster which is observed by EPR spectroscopy, and can be structurally characterised using X-ray crystallography and further rationalised with DFT calculations.

## Results and discussion

### Synthesis and characterisation of titanium-oxo clusters

**Fig. 2:**
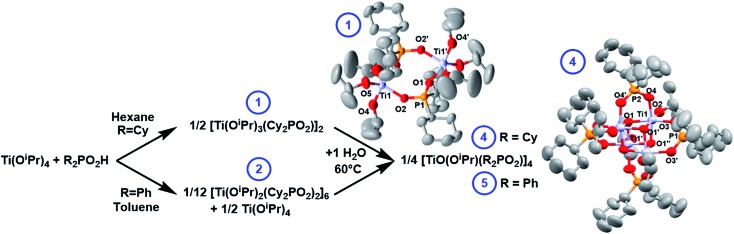
Reaction of Ti(O^i^Pr)_4_ with R_2_PO_2_H and subsequent hydrolysis, with solid-state structures of **1** and **4**, hydrogen atoms omitted for clarity. Ellipsoids displayed at 50% probability. Selected bond lengths (Å) and angles (°) **1**: P1–O1, 1.5054(17); P1–O2, 1.5260(17); Ti1–O1, 2.0050(16); Ti1–O2, 1.9837(16); Ti1–O^i^Pr (range), 1.793(2)–1.807(2); O1–Ti1–O2, 83.08(7); O1–Ti1–O4, 169.50(8). **4**: Ti1–O1, 1.937(2); Ti1–O1′, 2.121(2); Ti1–O1′′, 1.912(2); Ti1–O2, 1.790(2); Ti1–O3, 2.011(3); Ti1–O4, 2.021(3); P1–O3, 1.522(3); P2–O4, 1.518(3); Ti1···Ti1′, 2.9105(12); Ti1–O1–Ti1′, 98.25(10)–99.16(10); O3–P1–O3′, 114.2(2); O4–P2–O4′, 114.9(2).

The reaction of Ti(O^i^Pr)_4_ with one equivalent of dicyclohexylphosphinic acid (Cy_2_PO_2_H) forms the dimeric complex [Ti(O^i^Pr)_3_(Cy_2_PO_2_)]_2_ (**1**) and ^i^PrOH. An X-ray structure of **1** (Fig. 2) shows the phosphinate ligands bridge between the two Ti centres, which are 5-coordinate and adopt a pseudo trigonal-bipyramidal structure. This dimeric structure is consistent with solution NMR spectra collected in d_8_-toluene (Fig. S1–S7†).

The analogous reaction of Ti(O^i^Pr)_4_ with one equivalent of diphenylphosphinic acid (Ph_2_PO_2_H) in d^8^-toluene or CDCl_3_ solvent generates a set of nine signals in its ^31^P NMR spectrum (Fig. S8 and S10†); these nine signals are consistently present in an exact 1 : 1 : 2 : 1 : 3 : 1 : 1 : 1 : 1 ratio which suggests a single compound (**2**) with multiple ^31^P environments. The ^1^H NMR spectrum shows that half of the Ti(O^i^Pr)_4_ remains unreacted (Fig. S9†), suggesting that **2** has a formula [Ti(O^i^Pr)_2_(O_2_PPh_2_)_2_]_*n*_. **2** can be formed directly by reacting Ti(O^i^Pr) with two equivalents of Ph_2_PO_2_H, and isolated as a powder. The ^1^H NMR spectrum of isolated **2**, is consistent with the expected integrals for the formula [Ti(O^i^Pr)_2_(O_2_PPh_2_)_2_]_*n*_ with multiple phosphinate and alkoxide environments (Fig. S11†), however, no crystalline material of **2** was retrieved. It is expected that **2** is most likely an asymmetric hexameric species, with *n* = 6 (since the sum of ^31^P integrals = 12). A minor impurity species was also identified in some of the ^31^P NMR spectra of **2**, which has a 2 : 2 : 1 ratio of ^31^P signals (23.6, 20.5, 18.7 ppm) (Fig. S8 and S12–S14†); this species, previously observed by Schmidt and co-workers,^41^ could be separated from **2** due to its preferable solubility in hexane. A solid-state structure was collected characterising the complex as Ti_3_(O^i^Pr)_7_(Ph_2_PO_2_)_5_ (**3**) (Fig. S15†).

The remaining Ti–O^i^Pr bonds in **1** and **2** are susceptible to hydrolysis and condensation can be initiated with a controlled amount of water. **1** reacts with 1 equivalent of water to form a single compound (**4**) after heating at 60 °C overnight, with a single ^31^P NMR signal at 56.3 ppm (Fig. S16 and S17†). **4** crystallises from hexane at reduced temperatures as a tetrameric cluster, [TiO(O^i^Pr)(Cy_2_PO_2_)]_4_ (Fig. 2). The solid-state structure identified by single crystal X-ray diffraction is fully consistent with the solution ^1^H and ^31^P NMR spectra (Fig. S18–S20†). The structure is very similar to the previously reported [Ph_2_PO_2_]^–^ analogue, [TiO(O^i^Pr)(Ph_2_PO_2_)]_4_ (**5**).^42^,^43^ **5** may also be straightforwardly produced by adding two equiv. of water to a 1 : 1 mixture of **2** and Ti(O^i^Pr)_4_ and heating to 60 °C overnight (Fig. 2). Whilst **5** was previously reported as the DMSO or ^i^PrOH solvate, here **5** is crystallised from toluene as **5**·toluene (Fig. S21–25†).^42^,^43^ The structures of **4** and **5** relate to other phosphinate supported heterocubane clusters with a M_4_(μ_3_-O_4_) core (M = V, Mn, Mo, In, Sn, W) that have been previously reported;^42^,^44^–^48^

The solid-state structures of **4** and **5** reveal that the phosphinate ligands are delocalised with all P–O bonds of similar lengths. Each Ti centre adopts an approximately octahedral geometry, each coordinated to three oxo ligands, two phosphinate O donor atoms and an isopropoxide. Each Ti forms two shorter bonds to μ_3_-oxo centres (1.91–1.94 Å) and one slightly longer bond to the third μ_3_-oxo (2.12–2.16 Å), such that the structure may be viewed as two Ti_2_O_2_ square units bridged by four phosphinate ligands. The Ti–Ti distances within the Ti_2_O_2_ square unit are shorter than between the squares (2.91 Å *vs.* 3.07–3.12 Å respectively), with similar magnitudes to the Ti–Ti distances in brookite (2.95 & 3.06 Å).^49^

### UV spectroscopy and electronic structure of Ti-oxo clusters

**Fig. 3:**
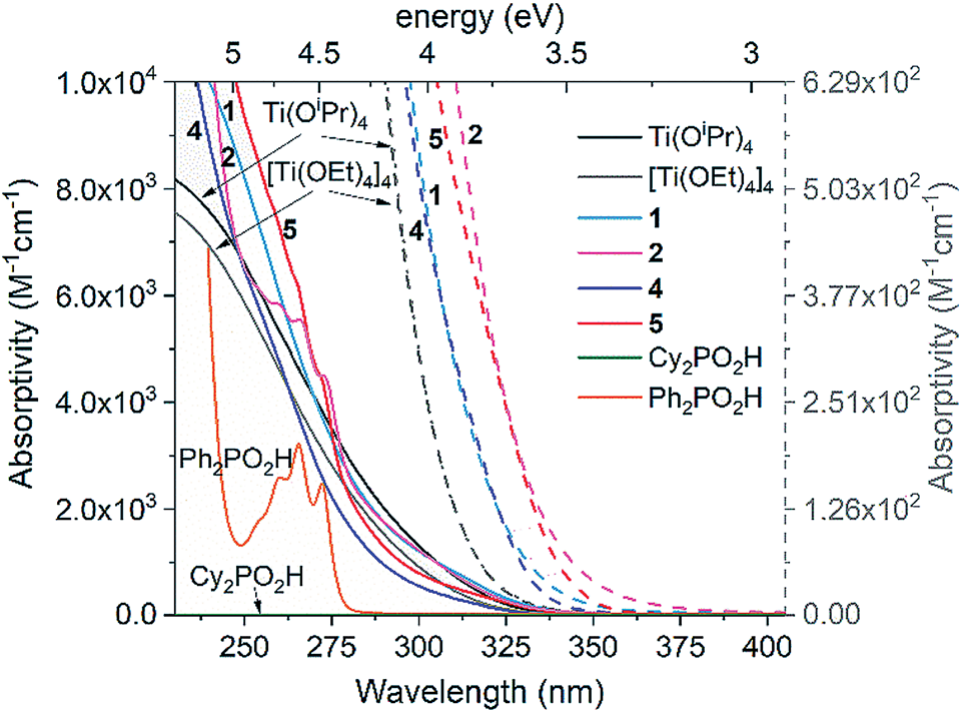
UV spectra of complexes **1**, **2**, **4** and **5** in comparison to Ti(OR)_4_ complexes and free R_2_PO_2_H ligands (see Fig. S26–S31† for spectra over a range of concentrations). Absorptivity of **1**, **2**, **4** and **5** determined in terms of concentration of Ti (M_[Ti]_^–1^ cm^–1^). Dashed lines show onset of absorptions to highlight the differences between the Ti species (collected with [Ti] = 1.75 mM), plotted *vs.* dashed scale bar to right hand side. Solvent = pentane (or CH_2_Cl_2_ for **2**, **5** and Ph_2_PO_2_H, N.B. identical spectra for **4** in pentane and CH_2_Cl_2_).

The UV spectra of precursor Ti(O^i^Pr)_4_ (and related tetramer [Ti(OEt)_4_]_4_), **1**, **2**, **4**, **5**, and free ligands R_2_PO_2_H (R = Ph or Cy) are shown in Fig. 3. They reveal that all Ti complexes show similar oxygen-to-metal charge transfer (OMCT) absorption onsets, ranging between 3.74–3.47 eV, which may be calculated using the Tauc plot method using spectra from relatively concentrated solutions ([Ti] = 1.75 and 0.88 mM, Fig. S26–S38†).^21^,^50^ All spectra show expected Beer–Lambert concentration profiles over the concentration range [Ti] = 1.75–0.05 mM. The precursor alkoxides [Ti(OR)_4_]_*n*_ (R = ^i^Pr or Et) exhibit essentially identical spectra and begin to absorb at higher energies (3.74 ± 0.2 eV) than the clusters. Introducing [Cy_2_PO_2_]^–^ in **1** or **4** causes a red shift in the absorption onset (**1**, 3.53 ± 0.1 eV; **4**, 3.59 ± 0.1 eV) and this effect is extended when the [Ph_2_PO_2_]^–^ ligand is used in **2** and **5** (**2**, 3.50 ± 0.1 eV; **5**, 3.47 ± 0.1 eV).^43^ The electronic absorptions in Ti(OR)_4_, **1**, **2**, **4** and **5** are considered to be OMCT in nature (see calculations below), in contrast to LMCT previously reported in dye functionalised titanium-oxo clusters and many Ti MOFs.^13^–^17^ π–π* transitions in the free Ph_2_PO_2_H ligand (and similar peaks in the spectra of **2** and **5**) occur at high energies (>4.5 eV) (Fig. 3) and are not expected to overlap with OMCT. **4** and **5** have an absorption onset similar to that of other carboxylate and alkoxide ligated Ti-oxo clusters^10^,^21^,^23^ but greater than the bandgap in bulk (>2 nm) TiO_2_. This is expected for small clusters which have discreet frontier orbitals rather than the extended band structure found in TiO_2_ (N.B. the Ti_4_O_16_ cores of **4** and **5** span around 0.64 nm, below the exciton Bohr radius of TiO_2_.^22^

**Fig. 4:**
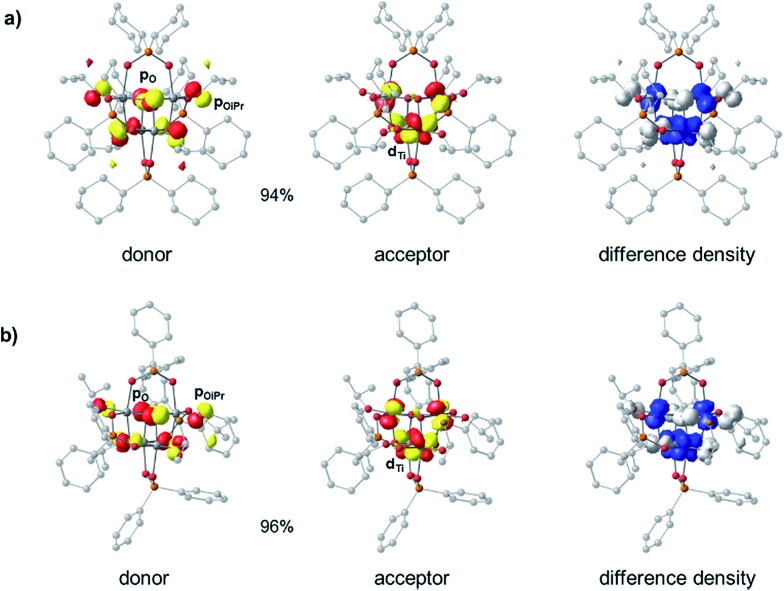
Dominant donor/acceptor NTO pairs (isovalue = 0.05 au) for the first electronic excitation of (a) **4** and (b) **5** (ZORA-B3LYP/def2-TZVP(-f)/def2-SV(P)), including percentage contributions of each pair to the transition. The isosurfaces of the electron difference densities between the excited and ground states are contoured at 0.003 au. White regions correspond to the depletion of the electron density during the transition while blue regions correspond to the accumulation of electrons.

Density Functional Theory (DFT) calculations were carried out in order to elucidate the electronic structures of the above titanium-oxo clusters. Optimised geometries of clusters **4** and **5** are in very good agreement with their X-ray crystallographic counterparts (Fig. S39 and Table S1†). As seen from the calculated frontier orbitals (Fig. S39–S41†), the HOMOs of both **4** and **5** are predominantly localised on the oxygen atoms (O 2p) with contributions from the oxo (**4**: 10.8%, **5**: 23.6%), alkoxide (**4**: 36.9%, **5**: 17.6%) and phosphinate (**4**: 15.7%, **5**: 2.0%) oxygen groups (Table S2†). There is also a minor contribution (∼7%) to the HOMO in **4** from the *ipso*-carbons of the cyclohexyl substituents. In **5** there is a negligible contribution of the diphenylphosphinate oxo centres to the HOMO orbital, possibly as a result of the more electron withdrawing Ph substituents, however, some admixture (∼29%) from phenyl π orbitals is present. Both LUMOs are similarly comprised of Ti 3d orbitals (82–85%), forming pairwise bonding interactions between adjoining Ti centres. The LUMO in **5** is slightly stabilised in energy by 0.06 eV compared to **4**. As a result, one finds an overall reduced HOMO–LUMO gap in **5** (4.88 eV) relative to **4** (4.94 eV), consistent with the experimental absorption onsets. It appears that the greater electron withdrawing nature of the phenyl substituent in the [Ph_2_PO_2_]^–^ ligand of **5**, relative to the cyclohexyl groups in **4**, subtly influences the nature of the frontier orbitals. We note that the calculations overestimate the energy gap relative to the experimental value, a phenomenon that is well-known for hybrid functionals due to their tendency to over-delocalise unoccupied states.^51^,^52^ The above results suggest that the first excited state in both **4** and **5** corresponds to a charge transfer from oxygen to the metal core (OMCT). The leading Natural Transition Orbitals (NTOs) generated from time-dependent DFT (TD-DFT) calculations of the singlet low-energy electronic transitions in **4** and **5** confirm that this is indeed the case (Fig. 4). Both clusters show a donor state comprised of a combination of μ_3_-oxo and alkoxide oxygens which upon excitation donates into a combination of the Ti 3d orbitals, with a high degree of similarity in their transition characters. The TD-DFT vertical excitation energies for **4** (3.97 eV) and **5** (3.93 eV) are lower than the calculated HOMO–LUMO gap as expected, and in reasonable agreement with experiment. Time-dependent calculations generally describe the optical gap more accurately (Table S3†), owing to the configuration interaction expansion of the electronically excited state as a linear combination of one-electron transition configurations. Whilst the changes in the energy gap between **4** and **5** are relatively small, it shows the opportunity to fine-tune the HOMO–LUMO energy gap and the relative orbital energies by subtle changes in ligand backbone, without affecting the nature of the HOMO–LUMO transitions (*i.e.* OMCT); a useful strategy for designing photocatalysts.

### Photo-redox reactivity

**Fig. 5:**
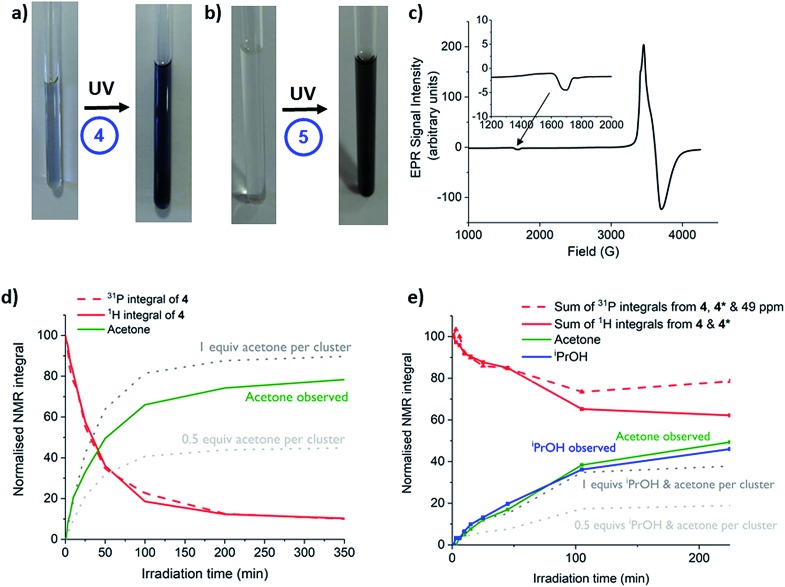
(a and b) Photographs of toluene solutions of **4** and **5** (with 30 equiv. ^i^PrOH) before and after irradiation (a), 13 h, MW-UV; (b), 3 h, LW-UV. (c) X-band EPR spectrum at 10 K of **4** after photo-reduction with MW-UV (7.5 h, [**4**] = 10 mM, 9 : 1 toluene : ^i^PrOH) with small signal at ∼1700 G enhanced. (d and e) Normalised NMR integrals of **4**, acetone and ^i^PrOH during MW-UV irradiation in toluene ((d): 30 equiv. ^i^PrOH; (e): 30 equiv. pyridine), dotted lines show the expected amount of acetone that will be produced by a 1-electron redox process (0.5 equiv. per cluster) or a 2-electron redox process (1 equiv. per cluster), estimated from the consumption of ^1^H integrals of **4**. N.B. 4* and the ^31^P signal at 49 ppm remain uncharacterised.

Complexes **1**, **2**, **4**, **5** and precursor Ti(O^i^Pr)_4_ were irradiated with long wave (LW-UV, 4 W) or medium wave (MW-UV, 6 W) UV lamps under inert atmosphere (N_2_) to investigate photo-redox reactivity (LW-UV: ‘365 nm’ output range ∼3.8–3.1 eV, MW-UV: ‘302 nm’ output range ∼4.4–3.3 eV). By absorbing a photon greater than the HOMO–LUMO gap, OMCT can occur, with an electron promoted to occupy a Ti based orbital. If the hole which forms upon an oxygen-based orbital is quenched, then a photo-reduced complex is formed in which Ti(iii) sites are found; these induce a colouration to the solution (typically blue).^24^,^28^,^53^ A small excess (∼30 equiv.) of ^i^PrOH was added to d^8^-toluene solutions of the complexes in glass NMR tubes (with an internal reference capillary of CH_2_Cl_2_ and PPh_3_ in CDCl_3_) and the samples irradiated with UV light (N.B. the glass tube will begin filtering photons > 3.7 eV). NMR spectra were collected after 1, 3 and 5 hours of irradiation. Both **1** and **2** slowly decompose under irradiation (LW or MW-UV) to give precipitates but give no blue colour (Fig. S42†); TiO^i^Pr_4_ is unreactive under LW-UV, but very slowly photo-reduces under MW-UV light to give a pale blue solution, with acetone produced as the oxidised component (Fig. S42 and S43†). Both **4** and **5** undergo photo-reduction under LW-UV light, highlighting lower energy photo-excitation in these complexes relative to TiO^i^Pr_4_. The photo-redox reaction of **4** and **5** occurs with conversion of some isopropoxide ligands into acetone (the oxidised product) and also isopropanol (maintaining proton balance, *vide infra*). The solution of **5** changes colour to a dark blue/grey colour without precipitate, and analysis of the NMR spectra reveal 88% consumption of **5** after 5 h (Fig. 5a and S44†, [**5**] = 9.4 μmol, [^i^PrOH] = 320 μmol); however, prolonged irradiation (>5 h) causes formation of a dark solid (*vide infra*). **4** undergoes slower photo-reduction with only 10% consumption after 4 h with LW-UV, but 88% if MW-UV is used ([**4**] = 9.7 μmol, [HO^i^Pr] = 320 μmol, Fig. S44†) to give a blue solution (Fig. 5a).

The presence of paramagnetic Ti(iii) species is confirmed by UV and EPR spectroscopy of the blue solutions. X-band EPR spectra of photo-irradiated solutions of **4** or **5** (frozen at 10 K) show resonance signals consistent with Ti(iii) centres (Fig. 5c and S45–S50†; for previously reported examples *g* = 1.84–1.99).^54^,^55^ Both spectra display a small signal at approximately 1700 G (Fig. 5c, S46 and S48†) corresponding to a formally-forbidden two-electron spin flip (Δ*M*_s_ = ±2), indicating the presence of complexes with *S* = 1, *e.g.* two unpaired electrons in a single molecule. Comparison of the spectra at various temperatures between 10 and 100 K indicate that the minor signal at 1700 G is retained over the entire temperature range (Fig. S46†). Upon cooling, the spectra broaden and shift to higher fields, indicating a decrease in the *g* value.^55^ Simulation of the spectra using an *S* = 1 model, gave a good agreement with the experimental results (Fig. S49 and S50†) and produced values of *g*_*xy*_ = 1.860 and *g*_*z*_ = 1.868 at 10 K (or 1.926 and 1.938 respectively at 100 K) for photo-reduced **4**, and *g*_*xy*_ = 1.880 and *g*_*z*_ = 1.905 for photo-reduced **5** (10 K). These spectra conclusively show the presence of *S* = 1 complexes in the photo-reduced solutions, although we cannot discount a contribution also from *S* = 1/2 species with similar *g* values.

The UV spectra after irradiation show broad absorptions across the visible region (maxima: from **5**, 693 nm; from **4**, ∼700 nm, with smaller peaks at 406 nm (**5**) and 480 nm (**4**) also observed, Fig. S51 and S52†). These absorptions are indicative of d–d transitions and/or intervalence Ti(iii)–Ti(iv) electron transfer.^29^,^37^,^53^ The presence of *S* = 1 clusters are also supported by the electronic absorption spectra predicted from TDDFT calculations. The absorption maxima positions for the photoreduced solutions are favourably reproduced (Fig. S53†) using doubly-reduced cluster models in their triplet ground states (*S* = 1). The d–d and intervalence transition character of these absorption bands is confirmed by difference density plots. The TDDFT spectra of singly-reduced cluster species (*S* = 1/2) were also calculated (Fig. S53†), suggesting that these could also play a role as chromophores in solution with absorption bands spanning a wide spectral range.

The irradiation of **5** in toluene without additional ^i^PrOH was also followed by NMR spectroscopy – in this case negligible photo-reduction occurs over 5 h. Various additives (alcohols, pyridine, THF and water) were tested, and all were found to increase the rate of photo-reduction (Fig. S54†). As ^t^BuOH, THF and pyridine were all found to accelerate photo-reduction, the additive is not required to undergo a redox transformation. Therefore, it appears that the additive plays a role as a donor ligand which may stabilise low coordinate Ti(iii) sites generated through photo-reduction. This is supported by the colour of the photo-reduced solutions which is dependent upon the additive or solvent.

**Fig. 6:**
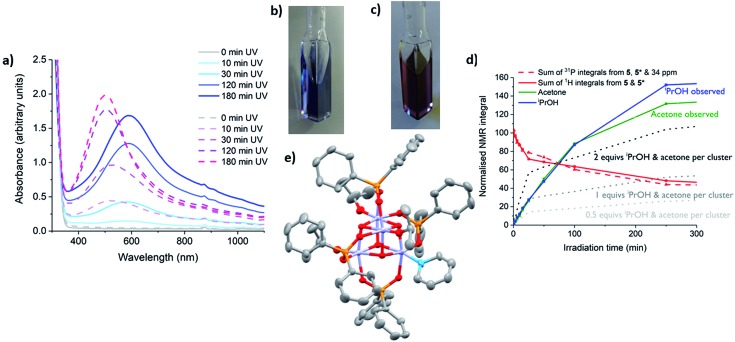
(a) UV/vis spectra of a solution of **5** under LW-UV irradiation in the presence of pyridine ([Ti] = 7.3 mM). Solid lines: toluene solution with 30 equiv. pyridine; dashed lines: neat pyridine solution. (b) photograph of a toluene solution of **5** with 30 equiv. pyridine after 3 h LW-UV photo-reduction. (c) Photograph of a pyridine solution of **5** after 3 h LW-UV photo-reduction. (d) Normalised NMR integrals of clusters, acetone and ^i^PrOH during irradiation with expected quantities of acetone produced relative to clusters consumed in the cases of 1, 2 and 4-electron photo-reduction per cluster shown by dotted lines. N.B. Extra minor ^31^P NMR signals (with a 2 : 1 ratio) were observed during prolonged irradiation; these were accompanied by Ti–O^i^Pr signals in the ^1^H NMR spectrum, suggesting a diamagnetic complex, and are termed ‘4*’ or ‘5*’. The signals of 4*/5* slowly convert back to **4**/**5** if left without irradiation. (e) Solid state structure of **6**, hydrogen atoms omitted for clarity. Ellipsoids displayed at 50% probability. **6** co-crystallises with **5** (27% **6**) with all atoms identically placed except for pyridine group (replacing an [O^i^Pr]^–^ group in **5**).

After photo-reaction of **4** or **5**, acetone and ^i^PrOH are observed by ^1^H NMR spectroscopy. It is known that alcohols can quench photogenerated holes,^24^,^53^ and in the photo-reaction some of the O^i^Pr substituents on **4** or **5** are oxidised to acetone. To proceed to acetone the [^i^PrO]^–^ unit must accept one hole (lose an electron) to form a radical [^i^PrO]˙ species followed by decomposition of the radical to give acetone, plus one electron and one proton. The excess proton can then react with a further [^i^PrO]^–^ substituent to form ^i^PrOH. Solutions of **4** and **5** with excess (∼30 equiv.) of either ^i^PrOH or pyridine were monitored by NMR spectroscopy during photo-irradiation (Fig. 5d, e, 6d and S55–S59†). The spectra confirm that the production of acetone is linked to the formation of the same quantity of ^i^PrOH, and both organic products grow in at the same rate as the consumption of starting cluster. This suggests that an overall two-electron reaction scheme occurs: [Ti_4_O_4_(O_2_PR_2_)_4_(O^i^Pr)_4_] → [Ti_4_O_4_(O_2_PR_2_)_4_(O^i^Pr)_2_]˙˙ + acetone + ^i^PrOH, in which two electrons are transferred to the cluster unit producing two Ti(iii) sites that are likely stabilised by donor solvents. Such a process resembles current doubling effects observed at TiO_2_ photoanodes in the presence of sacrificial electron donors (Fig. 1).^6^,^26^,^27^ Current doubling refers to the generation of two conduction band electrons after photo-excitation by a single photon, one electron being directly photo-excited, and the second electron injected into the conduction band after decomposition of the photo-oxidised organic radical. It is interesting that this effect is observed for a cluster molecule as this indicates that the second electron is preferentially donated to the originally photo-excited cluster – *e.g.* the entire process is likely intramolecular. Such findings are highly relevant for photocatalysis as two-electron processes are key for bond breaking/forming steps. It has been reported that photo-reduction of only up to 16% of the Ti atoms can occur in 3 nm amorphous TiO_2_ nanoparticles, therefore it is noteworthy that 50% of the Ti atoms are reduced in the small Ti_4_ clusters; perhaps the molecular nature and interaction with organic ligands makes a higher Ti(iii) content accessible.^32^,^33^

The photo-redox reaction of **4** or **5** in the presence of 30 equiv. pyridine occurs with the generation of a vivid blue colour (Fig. 6b). The intense blue colour suggests an additional electronic interaction occurs in the photo-reduced complexes in the presence of pyridine. This is attributed to the introduction of Ti(iii) to pyridine (π*) charge transfer absorptions, which are indeed implied by TDDFT calculations (Fig. S53†). A deep blue solution generated from irradiation of **5** (∼10 h, LW-UV) in toluene with 30 equiv. pyridine was concentrated under vacuum and deep blue crystals grew out of the blue solution.

The X-ray diffraction data revealed the expected unit cell for **5** and shows co-crystallisation of (colourless) starting material **5** (73%) alongside a photo-reduced complex, most likely [Ti(iv)_(4–*x*)_Ti(iii)_*x*_O_4_(O^i^Pr)_(4–*x*)_(O_2_PPh_2_)_4_(pyridine)_*x*_] (**6**) (27%), in which at least one anionic [^i^PrO]^–^ is replaced by a neutral pyridine. One pyridine is clearly modelled in the minor crystalline component, however, due to ligand disorder and the overlapping structure of **5**, it is difficult to determine if more than one coordinated pyridine is present (Fig S61 and ESI note 1†). The structure with *x* = 1 is shown in Fig. 6e and was also modelled using DFT calculations. The calculated frontier orbitals imply that in this *S* = 1/2 model the unpaired spin density is located predominantly upon the Ti atom coordinated to pyridine (55%) (Fig. S61 and Table S4†).

Whilst solutions of **4** and pyridine generate the expected 1 equiv. of acetone and ^i^PrOH per cluster consumed (Fig. 5e and S58†), solutions of **5** and pyridine begin in the same manner, but after 25 minutes irradiation the quantity of acetone/^i^PrOH rises to ∼2 equiv. per cluster consumed (Fig. 6d and S59†). This indicates a four-electron redox reaction in which all four Ti-O^i^Pr units of **5** are consumed, clearly highlighting the important role of the donor solvent. Furthermore, if pyridine is used as the neat solvent, photo-reduction of **5** yields a red/purple solution (Fig. 6a and c). In contrast, neat pyridine solutions of **4** irradiate to deep blue (Fig. S62†).

**Fig. 7:**
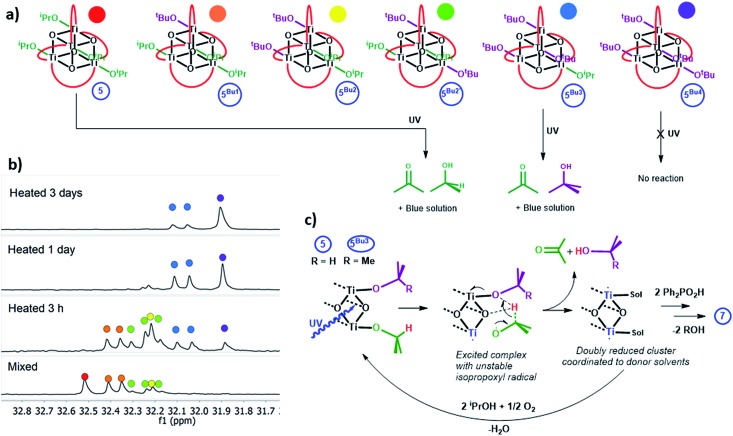
(a) Alkoxide exchange products from **5** and ^t^BuOH and the organic products released on irradiation with UV light (red curves represent [O_2_PPh_2_]^–^). (b) ^31^P {^1^H} NMR spectra of **5** + 300 equiv. ^t^BuOH in toluene after heating to 70 °C for various time periods. (c) Suggested intramolecular mechanism for formation of acetone and alcohol from one cluster unit, square Ti_2_O_2_ face shown for clarity.

Clusters **4** and **5** undergo slow alkoxide exchange in the presence of excess alcohols. The mixed alkoxide species [Ti_4_O_4_(O_2_PPh_2_)_4_(O^i^Pr)_*x*_(O^t^Bu)_*y*_] (*y* = 1, **5^Bu1^**; *y* = 2, **5^Bu2^** (2 isomers); *y* = 3, **5^Bu3^**; *y* = 4, **5^Bu4^**) can be prepared as mixtures during the slow alkoxide exchange of **5** with ^t^BuOH, and all can be identified by distinct ^31^P NMR signals (Fig. 7a, b and S63†; for the similar exchange reaction with EtOH see ESI note 2†). After heating **5** with 100 equiv. of ^t^BuOH at 70 °C for 7 days **5^Bu4^** was formed as the major species (^31^P NMR: 31.9 ppm, Fig. S64 and S65†). A minor doublet signal was also observed, due to a small proportion of **5^Bu3^**, with the compositions of both **5^Bu4^** and **5^Bu3^** confirmed by electrospray ionisation mass spectrometry (ESI-MS, Fig. S66†). A crystal structure of **5^Bu4^** was collected confirming the expected geometry (Fig. S67†). A solution of **5^Bu4^** with excess ^t^BuOH + 4 equiv. ^i^PrOH was exposed to LW-UV irradiation, however, no photo-reduction of **5^Bu4^** occurred, indicating that Ti-O^i^Pr units are essential for photo-reduction (as ^t^BuO^–^ cannot be oxidised) which must process *via* an intramolecular pathway. In contrast, mixtures of **5^Bu1^**, **5^Bu2^** and **5^Bu3^** all undergo photo-reduction, and interestingly **5^Bu3^** (as a mixture with **5^Bu4^** with 30 equiv. THF or pyridine) generates exclusively acetone and ^t^BuOH upon irradiation, with no production of ^i^PrOH (and no reaction of **5^Bu4^**, Fig. S68 and S69†). This supports an intramolecular, two-electron process in which acetone and alcohol are generated from the same cluster unit. Conversely, if an [^i^PrO]˙ radical was ejected from **5^Bu3^** (following a 1 electron photo-reaction) subsequent intermolecular reaction with a different cluster of **5^Bu3^** is expected to produce a mixture of ^t^BuOH and ^i^PrOH. Compound **4** undergoes similar alkoxide exchange reactions, albeit at a slower rate, and a solution of **4^Bu3^**/**4^Bu4^** similarly generates ^t^BuOH and acetone under MW-UV irradiation, with no production of ^i^PrOH. Whilst several mechanistic pathways are possible, a process is suggested in Fig. 7c, in which the photogenerated [^i^PrO]˙ radical is retained within the coordination sphere of the cluster, and rapid H atom transfer to the adjacent alkoxide group occurs across the Ti_2_O_2_ square face, to directly release acetone and alcohol.

Oxidation of the blue photo-reduced solutions by exposure to air results in an almost instantaneous colour change to pale yellow solutions. Various unidentified ^31^P NMR signals are observed directly after oxidation with air. If excess ^i^PrOH is present, upon standing in air the solution slowly regenerates starting material (*e.g.* 37% **5** reformed over 1 week). The regeneration of complex **5** under air completes a catalytic cycle for the oxidation of ^i^PrOH to acetone, with water also generated (see ESI note 3†). Understanding the effect of additives, solvents, and ligands is essential for designing effective photocatalysts, and the studies detailed here should be useful when utilising metal-oxo clusters or metal oxide materials.^56^

### Structural characterisation of photo-reduced clusters and their re-oxidation under air

**Fig. 8:**
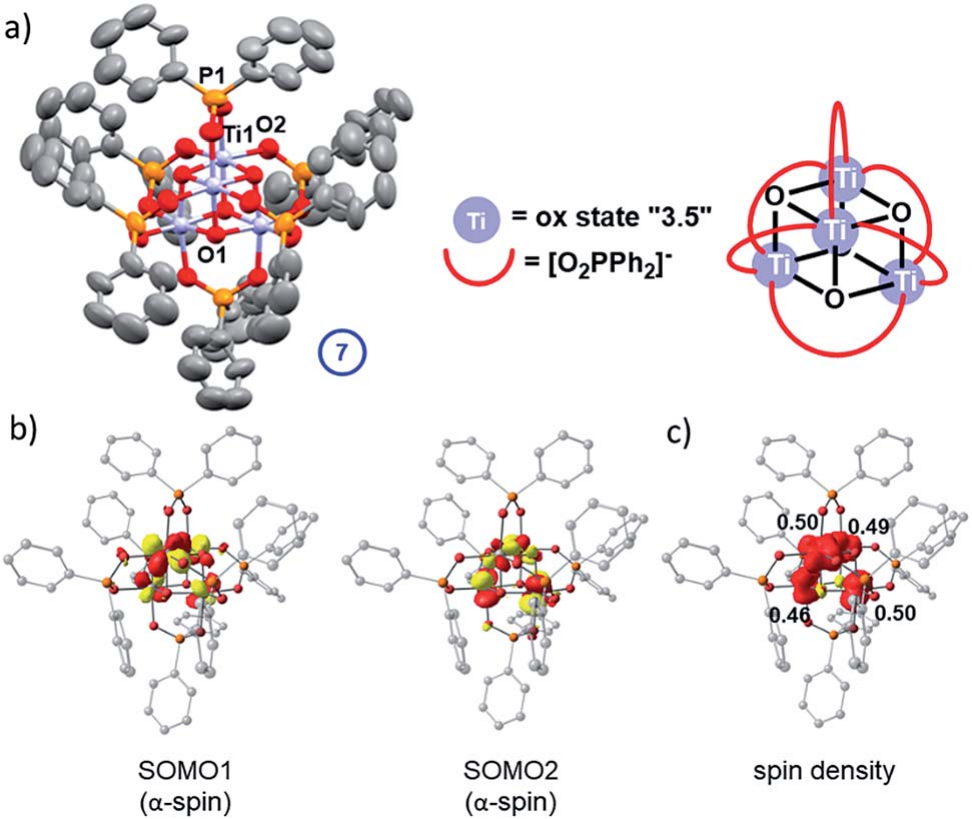
(a) Solid-state structure of **7** (hydrogen atoms omitted for clarity, ellipsoids displayed at 50% probability) selected bond lengths (Å) and angles (°) **7**: Ti1–O1, 1.970(2); Ti1–O2, 2.025(3); Ti1···Ti1′, 2.933(2); P1–O2, 1.522(4); Ti1–O1–Ti1′, 96.25(16), O2–P1–O2′ 115.9(3). (b) Calculated α-spin SOMOs (isovalue = 0.05 au); (c) a spin density plot and Loewdin atomic spin populations.

In the absence of strong donor solvents (*e.g.* pyridine), loss of alkoxide during the photo-reaction leaves a vacant coordination site at the Ti(iii) centres. This can be compensated by ligand exchange of one remaining monodentate alkoxide for a bidentate phosphinate. After irradiation of a toluene solution of **5** with 30 equiv. ^i^PrOH for >2.5 h, small dark coloured cubic crystals spontaneously form in the reaction flask overnight. The solid-state structure revealed these dark crystals to be comprised of high symmetry Ti_4_O_4_(Ph_2_PO_2_)_6_ (**7**, space group = *I*4[combining macron]3*m*) – a cluster that has retained the Ti_4_O_4_ core from **5** but which now has exclusively phosphinate ligands (Fig. 8a). The cluster is a symmetrical cube (Ti–O–Ti, 96.3°) with an average oxidation state of +3.5 per Ti centre, which is supported by bond-valence sum calculations (Table S5†). This indicates that two electrons occupy the Ti 3d based molecular orbitals in the cluster. The Ti-μ_3_–O bond length in **7** (1.970(2) Å) and Ti···Ti distance (2.933(2) Å) are intermediary to the longer and shorter distances found in asymmetrical **5**. **7** is isostructural with the previously reported mixed valent complex Mn_4_O_4_(Ph_2_PO_2_)_6_.^47^

The experimental findings are further corroborated by a DFT electronic structure analysis of **7**, which predicts the electronic triplet state to lie energetically below the closed-shell singlet (Δ*E* = +0.26 eV) and open-shell diradical singlet states (Δ*E* = +0.06 eV), the latter modelled using the broken-symmetry approach (Table S6 and Fig. S71–S73†). The symmetrical geometry of the Ti_4_O_4_ cluster core in the triplet ground state is well reproduced by the calculations, with average Ti-oxo and Ti···Ti distances of 1.95 Å and 2.90 Å, respectively (Fig. S70 and Table S7†). The unpaired electrons in two singly occupied molecular orbitals (SOMOs, Fig. 8b) are coupled ferromagnetically, resulting in a *S* = 1 total spin per molecular cluster. The spin density is evenly delocalised over four Ti centres with Loewdin populations of approximately 0.5 e^–^ per atom (Fig. 8c), fully consistent with the assigned average oxidation state of +3.5 per Ti centre and the EPR results.

The migration of two extra phosphinate ligands required in the formation of **7** suggests the phosphinate ligands may be exchanged in solution.^39^ The formation of **7** generates an insoluble material that crystallises, whilst the residual soluble Ti complexes remain uncharacterised. Irradiation of **5** in the presence of two extra equivalents of Ph_2_PO_2_H, generates **7** as an insoluble black powder which can be isolated and analysed by IR spectroscopy. The IR spectrum recorded under inert atmosphere shows a series of well-defined stretches consistent with a high symmetry molecule (Fig. S74†). Upon exposure to air, solid **7** rapidly changes colour to bright yellow/orange and the IR spectrum evolves to give broader, less-defined signals (Fig. S75†). We were only able to isolate mg scale quantities of very air sensitive **7** by the photo-reaction of **5** + 2 Ph_2_PO_2_H. Scale-up of the photo-reaction proved challenging due to slow reaction times, air sensitivity and the formation of mixtures of starting material and product, nevertheless, it was possible to analyse the product formed from oxidation of **7**. Solid-state EPR spectroscopy of this oxidised product (**7-ox**) at room temperature revealed a paramagnetic signal consistent with a superoxide anion (Fig. S76,† *g* = 2.017, 2.006, 2.001), implying the formation of a Ti(iv)-superoxide complex upon oxidation.^37^ The dication [Ti_4_O_4_(Ph_2_PO_2_)_6_]^2+^ could also be observed by ESI-mass spectrometry, after **7-ox** was dissolved in CH_2_Cl_2_ (Fig. S77†), suggesting a formulation for **7-ox** as [Ti_4_O_4_(Ph_2_PO_2_)_6_][O_2_]_2_.

**7-ox** appears to be rather stable in the solid-state under ambient conditions, with the superoxide signal observed by EPR spectroscopy several days after oxidation. The yellow/orange colour of the compound is similar to that observed in alkali metal and titanium based superoxides.^57^ Diffuse reflectance UV/visible spectroscopy showed a broad absorption with a maximum value at ∼412 nm (Fig. S78†) consistent with similar observations upon photo-reduced and re-oxidised TiO_2_ nanoparticles.^33^

**Fig. 9:**
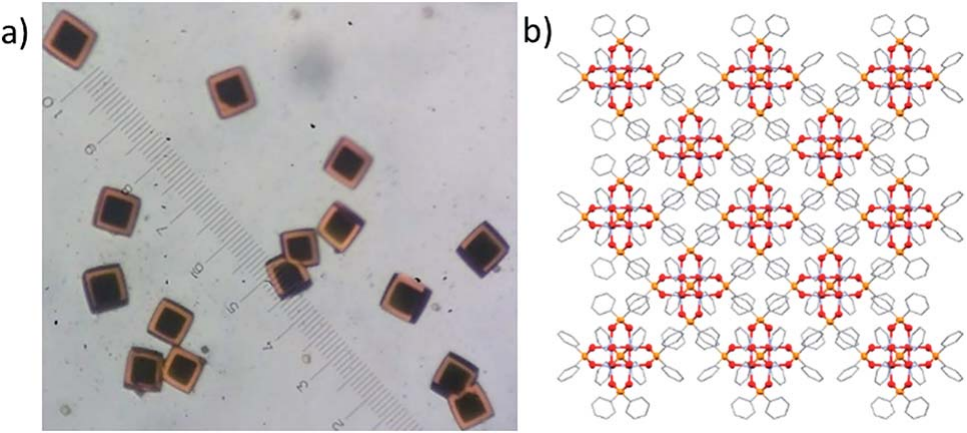
(a) Oxidation of single crystals of **7** under air. Crystals undergo reaction from surface downwards. (b) Packing diagram of **7** (with phenyl groups in stick notation and hydrogen atoms omitted) showing symmetrical nature of the complex and possibility for incorporation of small anions within the lattice.

Single crystals of **7** also react slowly with air to give yellow/orange **7-ox** whilst maintaining single crystallinity.^58^ The transition progresses smoothly through a bi-colour crystalline intermediate in which the edge of the crystal is oxidised whilst the centre remains in its reduced form (Fig. 9a). For ∼0.5 mm^3^ crystals, the oxidation requires ∼3 days under ambient conditions. The final yellow/orange crystal diffracts weakly at high angle, but the original unit cell is confirmed, and the data collected fits the structural model of **7** with an *R* factor of 14% (for **7**, *R* = 4%). The data suggest that the Ti_4_O_4_(Ph_2_PO_2_)_6_ unit is retained, although no other clear areas of electron density were identified. Whilst only approximate bond lengths and angles are available from this dataset (Fig. S79†), it is noteworthy that the Ti–phosphinate bonds in **7-ox** appear shortened compared to **7** (from 2.03 to ∼1.95 Å) and the Ti···Ti distances have slightly increased (from 2.93 to ∼3.01 Å); bond-valence sum analysis is now consistent with all Ti centres in the +4 oxidation state (Table S5†). It appears that the cluster is oxidised to [Ti_4_O_4_(Ph_2_PO_2_)_6_]^2+^ and, although the counterion has not yet been verified by crystallography (likely due to partially occupied identical sites in the high symmetry structure), the incorporation of [O_2_]^–^ into the lattice *via* reaction with air is consistent with a formula of [Ti_4_O_4_(Ph_2_PO_2_)_6_][O_2_]_2_ for **7-ox**. The pseudo-spherical shape of **7** influences the crystal packing, causing a body centred cubic arrangement within the unit cell. Small pockets between the clusters could possibly accommodate a superoxide anion upon oxidation. Titanium(iii) complexes can be used as colorimetric oxygen indicators,^59^ often useful in monitoring glovebox inert atmospheres, with an indicative blue to yellow colour change in the presence of O_2_; compound **7** acts as a solid-state example of this process. Furthermore, the ability to trap and store superoxide anions has interesting possibilities with respect to antibacterial surfaces or onward chemical reactivity.^5^,^7^,^57^

**Fig. 10:**
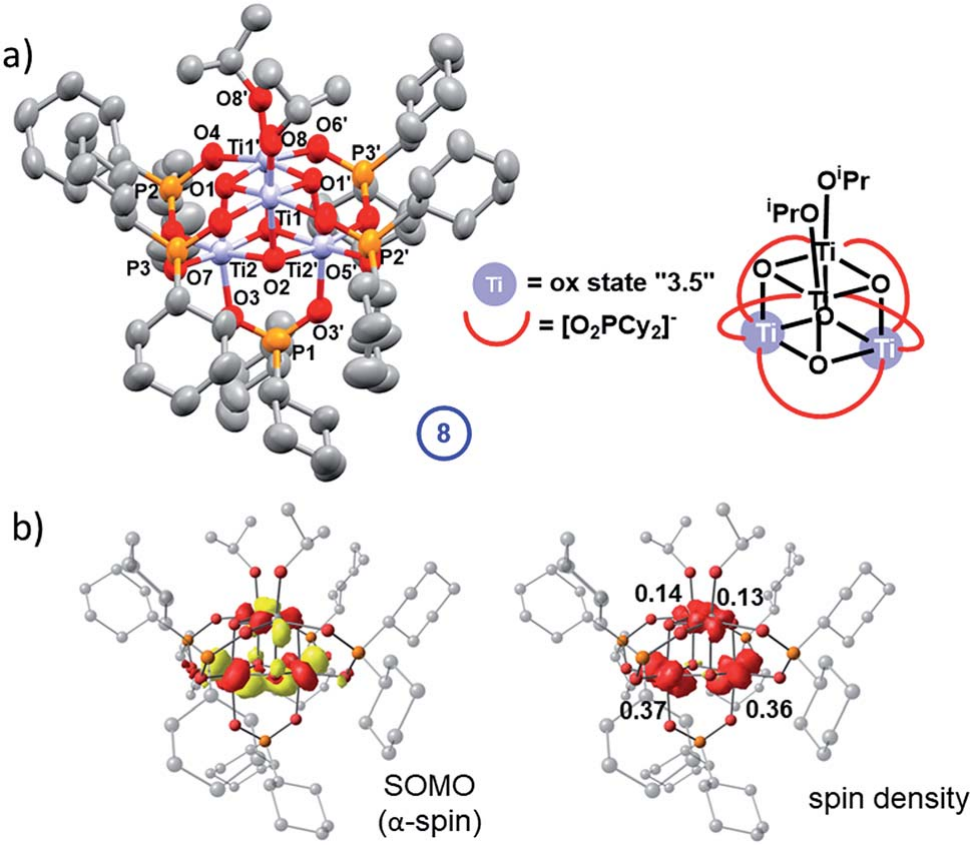
(a) Solid state structure of **8** (hydrogen atoms omitted for clarity, ellipsoids displayed at 50% probability). (b) Selected bond lengths (Å) and angles (°) **8**: Ti1–O1, 1.925(2); Ti1–O1′, 1.957(2); Ti1–O2, 2.056(2); Ti2–O1, 2.053(2); Ti2–O2, 1.9319(19); Ti2–O2′, 1.953(2); Ti1–O8, 1.795(3); Ti–OPOCy_2_ range, 2.010(2)–2.032(2); P–O range, 1.519(2)–1.528(2); Ti1···Ti1′, 2.9470(12); Ti2···Ti2′, 2.9114(12), Ti1···Ti2, 3.0080(8); Ti1···Ti2′; 3.0466(9), Ti1–O1–Ti1′, 98.76(9); Ti2–O2–Ti2′, 97.08(9); O–P–O range, 114.48(14)–115.52(19). Calculated α-spin SOMO (isovalue = 0.05 au) along with a spin density plot and Loewdin atomic spin populations.

**4** is rather soluble in hydrocarbon solvents, far more so than **5** (and **7**) due to the [Cy_2_PO_2_]^–^ ligand, however, blue crystals of a photo-reduced cluster could be slowly grown directly from a photo-irradiated solution of **4** after prolonged irradiation (with 30 equiv. ^i^PrOH) in hexane solvent. These blue crystals show a solid-state structure with the formula Ti_4_O_4_(Cy_2_PO_2_)_5_(O^i^Pr)_2_ (**8**) in which only one-electron reduction of the original cluster has occurred (Fig. 10a). **8** retains a structure comprised of two Ti_2_O_2_ squares with a longer Ti–O bond between the squares. Bond-valence sum calculations, supported by molecular orbital calculations, indicate that **8** contains two Ti(iv) sites (with coordinated alkoxide ligands) and two sites with average valence of 3.5 (Tables S5, S8 and Fig. S80–S82†), suggesting delocalisation of one 3d electron across two Ti centres (*e.g.* within one of the two Ti_2_O_2_ square units). This assessment is borne out in the contour surface of the SOMO and associated atomic spin populations (Fig. 10b), both indicating notable accumulation of electron spin (0.73 e^–^) on Ti2 and Ti2′ of the lower Ti_2_O_2_ square. It was very challenging to produce bulk samples of **8** in the absence of starting material **4**, which restricted analysis. Upon oxidation of mixtures of **8**/**4** under air, the powder turns a bright yellow/orange colour and solid-state EPR spectroscopy and diffuse-reflectance spectroscopy reveal signals for Ti(iv)-superoxide (from modelled EPR spectrum, *g* = 2.0182; 2.007; 2.0023, Fig. S78 and S83†), similar to that found for **7-ox**, such that **8-ox** is best formulated as [Ti_4_O_4_(O^i^Pr)_2_(Cy_2_PO_2_)_5_][O_2_]. The cation [Ti_4_O_4_(O^i^Pr)_2_(Cy_2_PO_2_)_5_]^+^ was also identified by ESI-MS after **8-ox** was dissolved in CH_2_Cl_2_ (Fig. S84†). The structure of **8** does not follow the two-electron photo-redox process supported by EPR and NMR experiments and may suggest that competing one-electron pathways are also possible; alternatively electron transfer may occur between photo-reduced species in solution, which may account the formation of crystals of **8** only after a prolonged UV exposure.

## Conclusions

Small phosphinate supported Ti_4_-oxo clusters have been prepared in a straightforward stoichiometric reaction. The clusters absorb LW-UV light *via* an oxygen-to-metal charge transfer process, with an absorption onset which is adjustable by ligand design. The absorption onset falls at higher energies than in bulk TiO_2_ consistent with other small Ti-oxo clusters and in line with quantum confinement effects, but at lower energies than simple Ti(OR)_4_ precursor molecules. The clusters undergo productive photo-redox reactivity under UV irradiation, with the oxidation of isopropoxide substituents to acetone (and isopropanol) and the formation of reduced Ti species containing Ti(iii) centres. EPR and NMR spectroscopy analysis of the irradiation process, alongside reactivity of mixed alkoxide cluster **5^Bu3^** are consistent with an intramolecular two-electron photo-redox pathway in which one molecule of acetone and alcohol are generated from the same cluster after photo-excitation. This leaves a two-electron reduced cluster, such as **7**. **7** is a rare example of a structurally characterised photo-reduced Ti-oxo cluster that exists in a triplet ground state. Such two-electron reactivity is consistent with current doubling effects observed at TiO_2_ photoanodes and is of interest with respect to photocatalysis in which a two-electron bond breaking/forming step is typically required. Reactivity of the clusters shows that both alkoxide and phosphinate ligands may undergo exchange in solution, and whilst competitive decomposition and/or one-electron photo-redox pathways remain possible, it is clear that a propensity for retention of the stable Ti_4_O_4_ core remains throughout these photo-reactions. Donor solvents, such as pyridine, accelerate the photo-redox process, supporting the formation of low-coordinate Ti(iii) centres. Understanding the effects of additives, solvents and ligands within metal-oxo clusters is important for developing their use in efficient photocatalytic processes. These studies should also give insight into photo-reactivity at the surfaces of metal oxides,^30^–^32^ for which detailed mechanistic study is often challenging, and are relevant in studies of partially reduced (black/blue) TiO_2_ used as a photocatalytic material.^34^–^36^ **7** and **8** react with oxygen to form superoxide complexes, and single crystals of **7** complete this reaction within the single crystalline phase, allowing for the product of oxidation to be studied directly by X-ray crystallography. These results show that productive photochemistry with charge separation can occur in even the smallest of metal-oxo clusters and highlight these molecules as useful systems for mechanistic studies.

## Experimental

All manipulations were undertaken using a nitrogen filled glovebox or using a Schlenk line, unless otherwise stated. Ph_2_PO_2_H and Ti(O^i^Pr)_4_ were used directly from suppliers, Cy_2_PO_2_H was synthesised by an amended literature prep^60^ (details included in the ESI†). As an air sensitive liquid, all additions of Ti(O^i^Pr)_4_ were transferred by syringe within the glovebox (measured by negative weight of donor flask). THF, hexane and toluene were dried by refluxing over sodium (and benzophenone for THF), pentane and dichloromethane were dried by refluxing over CaH_2_, all under nitrogen. d_8_-toluene was dried by stirring over CaH_2_, ‘extra-dry’ acetone was purchased from Acros Organics, these and all other dry solvents and reagents were degassed by bubbling with N_2_ for 30 minutes or freeze pump thaw cycles and stored over molecular sieves under nitrogen. The raw data that support the findings of this study are available from the University of Cambridge data repository, ; https://doi.org/10.17863/CAM.40665.

### **1** [Ti(O^i^Pr)_3_(Cy_2_PO_2_)]_2_

75 mg (0.33 mmol) of Cy_2_PO_2_H was placed in a Schlenk flask with a stirrer bar and dissolved in 3 mL of hexane. To this 93 mg (0.33 mmol) of TiO^i^Pr_4_ was added and the mixture stirred overnight. The solution was then evacuated to ∼0.5 mL and cooled to –20 °C overnight to yield colourless crystals. The remaining solution was removed, and the crystals dried under vacuum. 49 mg isolated crystalline yield (33%). ^31^P {^1^H} NMR spectroscopy (d^8^-toluene, 162 MHz): *δ* 45.5.

^1^H NMR spectroscopy (d^8^-toluene, 400 MHz): *δ* 5.09 (6H, sept (*J*_HH_ = 6 Hz), Ti-O^i^C**H**Me_2_), 1.35 (36H, d (*J*_HH_ = 6 Hz), Ti-O^i^CH**Me**_**2**_), 1.2–2.1 (44H, m, C_6_H_11_). Elemental analysis (predicted): % C, 55.30 (55.51); % H, 9.54 (9.54). N.B. **1** was not stable during ESI-MS, instead hydrolysis products [Ti_4_O_4_(O^i^Pr)_2/3_(Cy_2_PO_2_)_5/4_]^+^ were observed.

### **2** [Ti(O^i^Pr)_2_(Ph_2_PO_2_)_2_]_6_

76.8 mg (0.35 mmol) of Ph_2_PO_2_H was placed in a Schlenk and suspended in 2 mL CH_2_Cl_2_. To this 50 mg (0.18 mmol) of TiO^i^Pr_4_ was added. The solution was monitored directly by ^31^P NMR spectroscopy and some **3** is identified as a by-product. Removal of solvent led to a colourless solid. 50 mg isolated (44% yield). ^31^P {^1^H} NMR spectroscopy (CDCl_3_, 162 MHz): *δ* 23.9 (1P), 23.8 (1P), 23.6 (2P), 21.5 (1P), 21.3 (3P), 19.6 (1P), 19.5 (1P), 16.8 (1P), 16.6 (1P). ^31^P {^1^H} NMR spectroscopy (d^8^-toluene, 162 MHz): *δ* 24.3 (1P), 23.9 (1P), 23.8 (2P), 21.83 (1P), 21.79 (3P), 20.0 (1P), 19.9 (1P), 16.6 (1P), 16.4 (1P). ^1^H NMR spectroscopy (CDCl_3_, 400 MHz): *δ* 6.7–8.0 (120H, m, Ph_2_PO_2_), 5.02 (3H, septet, Ti-O^i^C**H**Me_2_), 4.92 (6H, septet, Ti-O^i^C**H**Me_2_), 4.85 (1H, septet, Ti-O^i^C**H**Me_2_), 4.49 (1H, septet, Ti-O^i^C**H**Me_2_), 4.03 (1H, septet, Ti-O^i^C**H**Me_2_), 1.26–0.68 (72H, m, Ti-O^i^CH**Me**_**2**_). Elemental analysis (predicted for [Ti(O^i^Pr)_2_(Ph_2_PO_2_)_2_]_6_·(2.7 CH_2_Cl_2_), N.B. sample precipitated from CH_2_Cl_2_ and thoroughly dried): % C, 57.30 (57.27); % H, 5.60 (5.51).

### **3** Ti_3_(O^i^Pr)_7_(Ph_2_PO_2_)_5_

64 mg (0.29 mmol) of Ph_2_PO_2_H was placed in Schlenk flask and suspended in 3 mL CH_2_Cl_2_. To this 50 mg (0.23 mmol) of TiO^i^Pr_4_ was added and the reaction mixture stirred for 1 hour. The solvent was removed under vacuum and 10 mL of hexane added to give a suspension. The flask was heated to 40 °C overnight whilst stirring and then the solution was filtered (and any solid discarded). The solvent was removed under vacuum to yield a white powdered solid. 25 mg isolated powder (26%). ^31^P {^1^H} NMR spectroscopy (d^8^-toluene, 162 MHz): *δ* 23.6 (2P), 20.5 (2P), 18.7 (P), ^1^H NMR spectroscopy (d^8^-toluene, 400 MHz): *δ* 8.08–7.76 (20H, Ph_2_PO_2_), 7.14–6.85 (30H, Ph_2_PO_2_), 5.37 (3H, br, Ti-O^i^C**H**Me_2_), 5.04 (4H, sept (*J*_HH_ = 6 Hz), Ti-O^i^C**H**Me_2_), 1.54–1.40 (18H, br, Ti-O^i^CH**Me**_**2**_), 1.26 (24H, d (*J*_HH_ = 6 Hz), Ti-O^i^CH**Me**_**2**_). Elemental analysis (predicted): % C, 58.15 (59.21); % H, 6.28 (6.07).

### **4** [TiO(O^i^Pr)(Cy_2_PO_2_)]_4_

810 mg (3.52 mmol) of Cy_2_PO_2_H was placed in a Schlenk flask with a stirrer bar and dissolved in 20 mL of hexane. To this 1 g (3.52 mmol) of TiO^i^Pr_4_ was added and the mixture stirred for 30 minutes. In a separate Schlenk, 63 μL of water was added to 1.5 mL of acetone, and this mixture added dropwise to the reaction solution whilst stirring. The solution was then heated to 50 °C for 48 hours whilst stirring, before concentrating under vacuum to ∼1 mL and cooling to –20 °C overnight to yield a white microcrystalline powder. The remaining solution was removed, and the powder dried under vacuum. 520 mg isolated crystalline yield (42%). ^31^P {^1^H} NMR spectroscopy (CDCl_3_, 162 MHz): *δ* 56.3. ^31^P {^1^H} NMR spectroscopy (d^8^-toluene, 162 MHz): *δ* 55.0. ^1^H NMR spectroscopy (CDCl_3_, 400 MHz): *δ* 5.04 (4H, sept (*J*_HH_ = 6 Hz), Ti-O^i^CHMe_2_), 2.15 (8H, d, Cy), 1.96 (8H, d, Cy), 1.77 (24H, m, Cy), 1.67 (8H, s, Cy), 1.49 (16H, m, Cy), 1.26 (24H, d (*J*_HH_ = 6 Hz), Ti-O^i^CHMe_2_), 1.19 (24H, m, Cy). ESI-MS (CH_2_Cl_2_): [M – H]^+^*m*/*z* = 1409.6, calc. 1409.5; [Ti_4_O_4_(O^i^Pr)_3_(Cy_2_PO_2_)_4_]^+^*m*/*z* = 1349.6, calc. 1349.5. Elemental Analysis (predicted): % C, 51.08 (51.15); % H, 8.25 (8.30).

### **5** [TiO(O^i^Pr)(Ph_2_PO_2_)]_4_·C_6_H_5_Me

768 mg (3.52 mmol) of Ph_2_PO_2_H was placed in a Young's tap flask with a stirrer bar and suspended in ∼30 mL of toluene. To this 1 g (3.52 mmol) of TiO^i^Pr_4_ was added and the mixture stirred for 30 minutes. In a separate Schlenk, 63 μL of water was added to 1.5 mL of dry acetone, and this mixture added dropwise to the reaction solution whilst stirring. The solution was then heated to 60 °C for 24 hours whilst stirring, before concentrating under vacuum to ∼2 mL and cooling to –20 °C overnight to yield a colourless crystalline product. The remaining solution was removed and the product dried under vacuum. 470 mg isolated crystalline yield (36%). ^31^P {^1^H} NMR spectroscopy (d^8^-toluene, 162 MHz): *δ* 32.5. ^1^H NMR spectroscopy (d^8^-toluene, 400 MHz): *δ* 8.01 (16H, m, Ph), 7.10 (8H, m, Ph), 7.02 (16H, m, Ph), 4.93 (4H, sept (*J*_HH_ = 6 Hz), Ti-O^i^C**H**Me_2_), 1.17 (24H, d (*J*_HH_ = 6 Hz), Ti-O^i^CH**Me**_**2**_). ESI-MS (CH_2_Cl_2_): [M – H]^+^*m*/*z* = 1361.0, calc. 1361.2; [Ti_4_O_4_(O^i^Pr)_3_(Ph_2_PO_2_)_4_]^+^*m*/*z* = 1301.0, calc. 1301.1. Elemental analysis (predicted for [TiO(O^i^Pr)(Ph_2_PO_2_)]_4_·(0.66 C_6_H_5_Me), note partial loss of solvent of crystallisation during drying): % C, 54.62 (54.61); % H, 5.18 (5.20).

### **5^Bu4^** [TiO(O^t^Bu)(Ph_2_PO_2_)]_4_·(C_6_H_5_Me)_0.5_

70 mg (0.05 mmol) of **5**·toluene was placed in a Schlenk and dissolved in 5 mL of toluene, to this 1.5 mL (18 mmol) of ^t^BuOH was added and the solution heated to 70 °C for 1 week. Over this period alkoxide exchange occurs and various [Ti_4_O_4_(O^i^Pr)_4–*x*_(O^t^Bu)_*x*_(Ph_2_PO_2_)_4_] (*x* = 1–4) complexes may be identified by NMR spectroscopy. After 1 week **5^Bu4^** may be crystallised from toluene at –20 °C. Isolated crystalline yield ∼50% (from small scale reaction). ^31^P {^1^H} NMR spectroscopy (d^8^-toluene, 162 MHz): *δ* 31.9. ^1^H NMR spectroscopy (d^8^-toluene, 400 MHz): *δ* 8.07 (16H, m, Ph), 7.10 (8H, m, Ph), 7.04 (16H, m, Ph), 1.39 (36H, s, O^t^Bu). ESI-MS (CH_2_Cl_2_): [M – H]^+^*m*/*z* = 1417.0, calc. 1417.2; [Ti_4_O_4_(O^t^Bu)_3_(Ph_2_PO_2_)_4_]^+^*m*/*z* = 1343.0, calc. 1343.1. Elemental Analysis predicted for [TiO(O^t^Bu)(Ph_2_PO_2_)]_4_·(0.5 C_6_H_5_Me): % C, 55.55 (55.43); % H, 5.38 (5.51).

### **7** [Ti_4_O_4_(Ph_2_PO_2_)_6_]

30 mg (0.02 mmol) of **5** and 50 μL of ^i^PrOH were dissolved in 2 mL toluene and irradiated with MW or LW-UV light for several hours and the blue/grey solution left to stand. Cubic grey crystals of **7** grow on the flask. Alternatively, 9 mg (0.04 mmol) of solid Ph_2_PO_2_H was suspended in the original solution before MW-UV irradiation for 3 hours generating a black microcrystalline product, the solution was removed and the solid further washed with CH_2_Cl_2_. 4 mg (12% yield) of **7** was isolated as a dark powder which turns bright yellow/orange on exposure to air. Elemental analysis after oxidation in air (predicted for [Ti_4_O_4_(Ph_2_PO_2_)_6_][O_2_]_2_): % C, 54.21 (53.30); % H, 3.94 (3.73). ESI-MS after oxidation in air (CH_2_Cl_2_): [Ti_4_O_4_(Ph_2_PO_2_)_6_]_4_^2+^*m*/*z* = 779.2, calc. 779.0.

### **8** [Ti_4_O_4_(Cy_2_PO_2_)_5_(O^i^Pr)_2_]

A sealed hexane solution of **4** was irradiated with MW-UV light for several hours and the blue solution left to stand. A small quantity of thin blue crystals of **8** grow on the flask. ESI-MS after oxidation in air (CH_2_Cl_2_): [Ti_4_O_4_(O^i^Pr)_2_(Cy_2_PO_2_)_5_]^+^*m*/*z* 1519.5, calc. 1519.6.

## Conflicts of interest

There are no conflicts to declare.

## Supplementary Material

Supplementary information

Crystal structure data

## References

[cit1] Chen X., Mao S. S. (2007). Chem. Rev..

[cit2] Xu H., Ouyang S., Liu L., Reunchan P., Umezawa N., Ye J. (2014). J. Mater. Chem. A.

[cit3] Banerjee S., Dionysiou D. D., Pillai S. C. (2015). Appl. Catal., B.

[cit4] Bai Y., Mora-Seró I., De Angelis F., Bisquert J., Wang P. (2014). Chem. Rev..

[cit5] Fagan R., McCormack D. E., Dionysiou D. D., Pillai S. C. (2016). Mater. Sci. Semicond. Process..

[cit6] Schneider J., Matsuoka M., Takeuchi M., Zhang J., Horiuchi Y., Anpo M., Bahnemann D. W. (2014). Chem. Rev..

[cit7] Li Y., Zhang W., Niu J., Chen Y. (2012). ACS Nano.

[cit8] Fang W.-H., Zhang L., Zhang J. (2018). Chem. Soc. Rev..

[cit9] Coppens P., Chen Y., Trzop E. (2014). Chem. Rev..

[cit10] Fang W.-H., Zhang L., Zhang J. (2016). J. Am. Chem. Soc..

[cit11] Matthews P. D., King T. C., Wright D. S. (2014). Chem. Commun..

[cit12] Fan X., Wang J., Wu K., Zhang L., Zhang J. (2019). Angew. Chem., Int. Ed..

[cit13] Wu Y.-Y., Lu X.-W., Qi M., Su H.-C., Zhao X.-W., Zhu Q.-Y., Dai J. (2014). Inorg. Chem..

[cit14] Negre C. F. A., Young K. J., Oviedo M. B., Allen L. J., Sánchez C. G., Jarzembska K. N., Benedict J. B., Crabtree R. H., Coppens P., Brudvig G. W., Batista V. S. (2014). J. Am. Chem. Soc..

[cit15] Hou J., Zhang Q., Wu Y., Liu Y., Du L., Tung C.-H., Wang Y. (2016). Inorg. Chim. Acta.

[cit16] Zou D.-H., Cui L.-N., Liu P.-Y., Yang S., Zhu Q.-Y., Dai J. (2018). Chem. Commun..

[cit17] Zhu J., Li P.-Z., Guo W., Zhao Y., Zou R. (2018). Coord. Chem. Rev..

[cit18] Rozes L., Sanchez C. (2011). Chem. Soc. Rev..

[cit19] Boyle T. J., Tyner R. P., Alam T. M., Scott B. L., Ziller J. W., Potter B. G. (1999). J. Am. Chem. Soc..

[cit20] Eslava S., McPartlin M., Thomson R. I., Rawson J. M., Wright D. S. (2010). Inorg. Chem..

[cit21] Benedict J. B., Coppens P. (2010). J. Am. Chem. Soc..

[cit22] Satoh N., Nakashima T., Kamikura K., Yamamoto K. (2008). Nat. Nanotechnol..

[cit23] Liu J.-X., Gao M.-Y., Fang W.-H., Zhang L., Zhang J. (2016). Angew. Chem., Int. Ed..

[cit24] Hu J., Zhan L., Zhang G., Zhang Q., Du L., Tung C.-H., Wang Y. (2016). Inorg. Chem..

[cit25] Hanf S., Matthews P. D., Li N., Luo H.-K., Wright D. S. (2017). Dalton Trans..

[cit26] Hykaway N., Sears W. M., Morisaki H., Morrison S. R. (1986). J. Phys. Chem..

[cit27] Yang J., Liu B., Xie H., Zhao X., Terashima C., Fujishima A., Nakata K. (2015). J. Phys. Chem. C.

[cit28] Bueken B., Vermoortele F., Vanpoucke D. E. P., Reinsch H., Tsou C.-C., Valvekens P., De Baerdemaeker T., Ameloot R., Kirschhock C. E. A., Van Speybroeck V., Mayer J. M., De Vos D. (2015). Angew. Chem., Int. Ed..

[cit29] Wu Y.-Y., Luo W., Wang Y.-H., Pu Y.-Y., Zhang X., You L.-S., Zhu Q.-Y., Dai J. (2012). Inorg. Chem..

[cit30] Valdez C. N., Braten M., Soria A., Gamelin D. R., Mayer J. M. (2013). J. Am. Chem. Soc..

[cit31] Schimpf A. M., Gunthardt C. E., Rinehart J. D., Mayer J. M., Gamelin D. R. (2013). J. Am. Chem. Soc..

[cit32] Schrauben J. N., Hayoun R., Valdez C. N., Braten M., Fridley L., Mayer J. M. (2012). Science.

[cit33] Kormann C., Bahnemann D. W., Hoffmann M. R. (1988). J. Phys. Chem..

[cit34] Chen X., Liu L., Yu P. Y., Mao S. S. (2011). Science.

[cit35] Yin G., Huang X., Chen T., Zhao W., Bi Q., Xu J., Han Y., Huang F. (2018). ACS Catal..

[cit36] Liu N., Schneider C., Freitag D., Hartmann M., Venkatesan U., Müller J., Spiecker E., Schmuki P. (2014). Nano Lett..

[cit37] Mason J. A., Darago L. E., Lukens W. W., Long J. R. (2015). Inorg. Chem..

[cit38] Huffman J. C., Stone J. G., Krusell W. C., Caulton K. G. (1977). J. Am. Chem. Soc..

[cit39] Pike S. D., White E. R., Shaffer M. S. P., Williams C. K. (2016). Nat. Commun..

[cit40] Schubert U. (2005). J. Mater. Chem..

[cit41] Chakraborty D., Chandrasekhar V., Bhattacharjee M., Krätzner R., Roesky H. W., Noltemeyer M., Schmidt H.-G. (2000). Inorg. Chem..

[cit42] Guerrero G., Mehring M., Hubert Mutin P., Dahan F., Vioux A. (1999). J. Chem. Soc., Dalton Trans..

[cit43] The UV absorption onset of **5** in the solid state has been previously reported as 3.6 eV using diffuse reflectance UV spectroscopy. Huang Y. Zou G.-D. Li H.-M. Cui Y. Fan Y., New J. Chem., 2018, 4217 , 14079 –14082 .

[cit44] Feng L., Maass J. S., Luck R. L. (2011). Inorg. Chim. Acta.

[cit45] Maass J. S., Chen Z., Zeller M., Luck R. L. (2011). Dalton Trans..

[cit46] Wu J.-Z., Sellitto E., Yap G. P. A., Sheats J., Dismukes G. C. (2004). Inorg. Chem..

[cit47] Ruettinger W. F., Campana C., Dismukes G. C. (1997). J. Am. Chem. Soc..

[cit48] Arif A. M., Barron A. R. (1988). Polyhedron.

[cit49] In Non-Tetrahedrally Bonded Binary Compounds II, ed. O. Madelung, U. Rössler and M. Schulz, Springer Berlin Heidelberg, Berlin, Heidelberg, 2000, pp. 1–5.

[cit50] Tauc J., Menth A., Wood D. L. (1970). Phys. Rev. Lett..

[cit51] Baerends E. J., Gritsenko O. V., van Meer R. (2013). Phys. Chem. Chem. Phys..

[cit52] Garza A. J., Scuseria G. E. (2016). J. Phys. Chem. Lett..

[cit53] Dan-Hardi M., Serre C., Frot T., Rozes L., Maurin G., Sanchez C., Férey G. (2009). J. Am. Chem. Soc..

[cit54] Morra E., Giamello E., Chiesa M. (2017). J. Magn. Reson..

[cit55] Doyle L. R., Wooles A. J., Jenkins L. C., Tuna F., McInnes E. J. L., Liddle S. T. (2018). Angew. Chem., Int. Ed..

[cit56] Lechner M., Güttel R., Streb C. (2016). Dalton Trans..

[cit57] Dewkar G. K., Nikalje M. D., Sayyed Ali I., Paraskar A. S., Jagtap H. S., Sudalai A. (2001). Angew. Chem., Int. Ed..

[cit58] Pike S. D., Weller A. S. (2015). Philos. Trans. R. Soc., A.

[cit59] Burgmayer S. J. N. (1998). J. Chem. Educ..

[cit60] Wang Y., Liu Q., Shi L.-l., Cheng C.-m., Chen L.-q. (2012). Huaxue Shiji.

