# Supplementary material for: Photo-redox reactivity of titanium-oxo clusters: mechanistic insight into a two-electron intramolecular process, and structural characterisation of mixed-valent Ti(iii)/Ti(iv) products
Source: Chem Sci. 2019 Jun 7;10(28):6886–98. doi: 10.1039/c9sc01241a (PMC6640198; doi:10.1039/c9sc01241a)
Supplement: Supplementary file 1 [file SC-010-C9SC01241A-s001.pdf]

Electronic Supplementary Material (ESI) for Chemical Science.
This journal is © The Royal Society of Chemistry 2019

Photo-redox reactivity of titanium-oxo clusters: mechanistic insight into a two-electron intramolecular process, and structural characterisation of mixed-valent Ti(III)/Ti(IV) products.

Supporting Information

Tobias Krämer,[b] Floriana Tuna [c] and Sebastian. D. Pike *[a]

a. Department of Chemistry, University of Cambridge, Lensfield Road, CB2 1EW, UK

b. Department of Chemistry, Maynooth University, Maynooth, Co. Kildare, Ireland

c. School of Chemistry and Photon Science Institute, University of Manchester, Oxford Road, Manchester, M13 9PL, UK

Contents

Experimental Details	2
Tauc plot method for determining absorption onset	2
Computational Methods	3
Synthesis of Cy_2PO_2H	3
Supporting discussion of the structure of **1**	4
Characterisation for **1.** $[Ti(O^iPr)_3(Cy_2PO_2)]_2$	4
Characterisation for **2.** $[Ti(O^iPr)_2(Ph_2PO_2)_2]_x$ (expected x = 6)	8
Characterisation for **3.** $Ti_3(O^iPr)_7(Ph_2PO_2)_5$	10
Characterisation for **4.** $[TiO(O^iPr)(Cy_2PO_2)]_4$	12
Characterisation for **5.** $[TiO(O^iPr)(Ph_2PO_2)]_4.C_6H_5Me$	14
UV Spectroscopy Data	17
DFT calculations for **4** and **5**	24
Photoirradiation Experiments (NMR, EPR and UV spectroscopy)	29
X-ray crystallography and DFT calculations for **6**	39
Characterisation of $\mathbf{5^{Bux}}$ compounds	41
Photoirradiation of $\mathbf{5^{Bu3}}$	44
Bond-valence sum calculations	45
Characterisation and DFT calculations for **7**, $[Ti_4O_4(Ph_2PO_2)_6]$ and **7-ox,** $[Ti_4O_4(Ph_2PO_2)_6][O_2]_2$	45
Characterisation and DFT calculations for **8,** $[Ti_4O_4(Cy_2PO_2)_5(O^iPr)_2]$ and **8-ox** $[Ti_4O_4(Cy_2PO_2)_5(O^iPr)_2][O_2]$	52
Crystallography information	57
Supporting notes. (1. *Refinement of co-crystal **5/6***; 2. *Alkoxide exchange of **5** with EtOH and photoreaction of products*; 3. *Photocatalysis*; 4. *Investigating paramagnetic NMR spectroscopy signals*	60
References	63

Experimental Details

NMR spectra were recorded on Bruker Avance III HD 400 or 500 MHz instruments and all chemical shifts reported in parts per million (ppm). Solid-state Fourier transform infrared spectra were recorded using a Perkin-Elmer Spectrum One FT-IR spectrometer or using an Agilent Technologies Cary 630 FT-IR spectrometer situated in a glovebox, both with an ATR Sampling Accessory. Solution ultraviolet spectroscopy was recorded using a PerkinElmer Lambda 750 spectrometer using a bespoke Young's tap cuvette for air free analysis. Diffuse reflectance UV spectroscopy was recorded on a Varian Cary 50 spectrophotometer using a Harrick Scientific Video Barrelino probe. All mass spectrometry measurements were performed using a Micromass Quattro LC spectrometer. Elemental Analysis was determined using a Perkin Elmer 240 Elemental Analyzer by the Microanalyis Laboratory at the Department of Chemistry, University of Cambridge. Powder EPR experiments were performed at room temperature using a Bruker EMX Micro EPR spectrometer operating at X-band (ca. 9.84 GHz). Frozen solution EPR experiments were performed with a Bruker EMX 300 EPR Spectrometer operating at X-band (ca 9.4 GHz) and variable temperatures, and equipped with an ESR 900 liquid He cryostat. EPR Data presented were modelled with the spin hamiltonian

$$H = g\beta B \cdot S + D\{S_Z^2 - S(S+1)/3\} + E(S_X^2 - S_Y^2)$$

where S=1, and D and E are the axial and rhombic zero-field splitting terms.

Photoirradiation was undertaken using a Model UVGL-25 mineralight lamp (output 366 nm, 4 W) or a Analytik Jena UVLM-26 EL series UV lamp (output 302 or 365 nm, 6 W). Experiments were conducted under a plastic cover to avoid exposure to UV. According to the manufacturers available data we anticipate the output for long wave UV irradiation to range from 325-400 nm, with a maximum output at 365/366 nm, and for the medium wave UV irradiation to range from 280-375 nm, with a maximum output at 302 nm. It is noteworthy that glass flasks begin to absorb photons <335 nm and will reduce the flux of any high energy photons from medium wave UV irradiation.

For in-situ analysis of photoreactions by NMR spectroscopy the starting material **4** or **5** (7 mg, ~5 µmol) and a sealed capilliary containing PPh_3 and CH_2Cl_2 dissolved in $CDCl_3$ was loaded into a Young's tap NMR tube inside a glovebox. Solvent (typically d^8-toluene, 0.5 mL) and 30 equiv. of additive (e.g. ^{i}PrOH, 12.5 µL) were added to the tube via syringe and micropipette respectively under a flow of N_2 using Schlenk line apparatus. The tube was sealed and then cooled in to −78°C in a dry ice acetone bath before exposure to vacuum, this cooling/vacuum proceedure was repeated once more to ensure an air free environment before photoirradiation. The capilliary was tipped to the top of the tube and the tube laid in front of the UV lamp so that the solution (but not the capilliary) was in the path of UV light, ~2 cm from the lamp. A similar proceedure was used for in-situ UV spectroscopic analysis of photoreactions (conc of **4** or **5** = ~ 1.8 mM).

Tauc plot method for determining absorption onset

UV spectra were collected over a range of concentrations ([Ti] = 1.75-0.05 mM, Figs. S26-31) with a spectrum for pure solvent subtracted to give an accurate baseline. All results obey Beer-Lambert behaviour. The two strongest concentrations were used to calculate the absorption onset using the Tauc plot method.[1] The absorption onset is described by:

$$A = \frac{B(hv - E_g)^n}{hv}$$

Where A is absorption, B is the absorption constant for the transition, E_g is the energy gap in eV and $h\nu$ is the photon energy. Absorption was most accurately modelled with a cubic dependence on increasing energy (Fig. S32), in keeping with previous studies of titanium-oxo clusters,[2] and therefore the exponent n is given a value of 3 (for bulk semiconductors this is considered a forbidden direct transition). A plot of $(Ah\nu)^{1/3}$ vs $h\nu$ gives a straight line (Figs S33-38). The point of absorption onset (in eV) was derived at the x-value of the intercept of this straight line with a straight line describing the baseline (to avoid any error from extrapolating through an imperfect baseline). The onsets determined from both concentrations were in excellent agreement, generally within 0.01 eV.

Computational Methods

Geometries of all computational model clusters were optimized without imposing any symmetrical constraints at the B97-3c[3] level of theory as implemented in ORCA (version 4.0.1.2).[4] This method is based on the B97 GGA functional[5] including a D3 with thee-body contribution[6, 7], a short-range bond length correction, and a modified def2-mTZVP basis set of triple-ζ quality. Initial coordinates were extracted from the corresponding X-ray crystallographic data, where available. Optimized stationary points were checked to correspond to minima by numerical harmonic vibrational analysis at the same level of theory. Subsequent single-point calculations on optimized geometries utilized the B3LYP[8-10] hybrid functional in conjunction with the recontracted def2-TZVP(–f) basis set[11] for Ti, O, N and P centres along with the recontracted def2-SV(P) basis[12] on the remaining C and H centres. Scalar relativistic effects were incorporated by the all-electron zeroth-order regular approximation (ZORA).[13] The validity of the B97-3c approach for geometry optimizations was tested by comparing optimized bond parameters of **4** and **5** against those obtained with the ZORA-B86-D3[8,14]/def2-TZVP(-f)/def2-SVP level of theory. The FlipSpin feature of ORCA was used to generate the initial guess for the broken-symmetry[15] calculation. The RIJCOSX[16, 17] approximation was used along with a def2/J auxiliary basis set[18] to speed up calculations. The electronic structure was analysed by means of canonical and corresponding molecular orbitals and population analysis based on the Löwdin orbital partitioning scheme.[19] Excited states were evaluated by means of time-dependent density functional theory (TDDFT),[20] performed on optimised ground state geometries. A range of functionals were tested (B3LYP, CAM-B3LYP[21], ωB97xD3[22], M06[23], PBE[24], PBE0[25]). Bulk electrostatic interactions due to the presence of a solvent were modelled using the conductor-like polarizable continuum model (CPCM), while short-range interactions between solute and solvent molecules in the first solvation shell contributions (CDS terms) were added through the SMD model.[26] Relevant parameters corresponded to those of toluene. In order to facilitate the analysis of the TDDFT results, Natural Transition Orbitals (NTOs) were generated.[27] Commonly electronic excitations are dominated by only a few NTO transitions, and these provide an easier means for understanding transitions than the excitation amplitudes in the canonical molecular orbital basis. According to the weight to which they contribute to an excitation, NTOs reflect the main character of excited states originating from promotion of an electron between a donor/acceptor orbital pairs.

Synthesis of Cy_2PO_2H

The synthesis of Cy_2PO_2H was conducted using an amended literature preparation.[28] 6g (56.6 mmol) of sodium hypophosphite monohydrate and 14.4 mL (141.6 mmol) of cyclohexene were dissolved in 50 mL acetic acid and to this 1.3 mL (7.07 mmol) tert-butyl peroxide was added. The solution was split into 5x 23 mL autoclaves, sealed and heated to 135°C for 2.5 days. After cooling the solutions

were recombined and acidified with 10% H_2SO_4(aq), the flask was then left in a fumehood to allow volatile organics to evaporate, leaving a crystalline white precipitate in the aqueous phase. The solid was washed with further acid and then water (twice) before dissolving in hot hexane and further washing with water. Finally, the solid may be recrystallised from hot hexane until it appears white and pure by analysis. Yield = 4.4 g (34%).

Supporting discussion of the structure of 1.

In the solid-state structure of **1** the phosphinate ligand adopts a bridging coordination mode, with one P−O bond slightly longer than the other (P1−O1, 1.505(2) Å; P1−O2, 1.526(2)) indicating a greater double bond character remains in one bond.[29] The longer P−O− bond forms a slightly shorter O−Ti bond, which occupies an axial site.

1 exhibits a single ^{31}P NMR signal (45.5 ppm) in d_8-toluene (Figs. S1 & S2), however, when dissolved in $CDCl_3$ a more complex NMR spectrum is observed, which shows a series of broad, temperature-dependent signals, and may suggest oligomerisation occurs under these conditions (Figs. S3-S7). The single ^{31}P environment is re-established on dissolving the same sample once more in toluene. Similar dimer-oligomer solution behaviour has been noted for carboxylate analogue [Ti(O_2CCMe_3)(OCH_2CMe_3)].[30]

Characterisation for 1. [Ti(O^iPr)$_3$(Cy_2PO_2)]$_2$

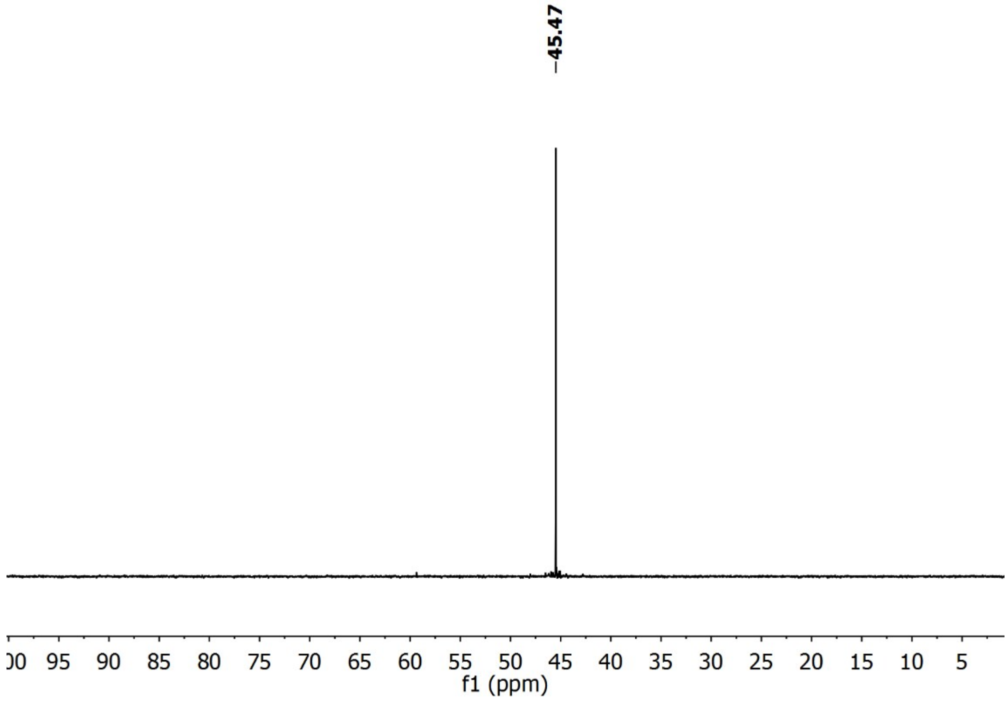

Figure S1. ^{31}P {^{1}H} NMR spectrum for **1** (d^8-toluene, 162 MHz)

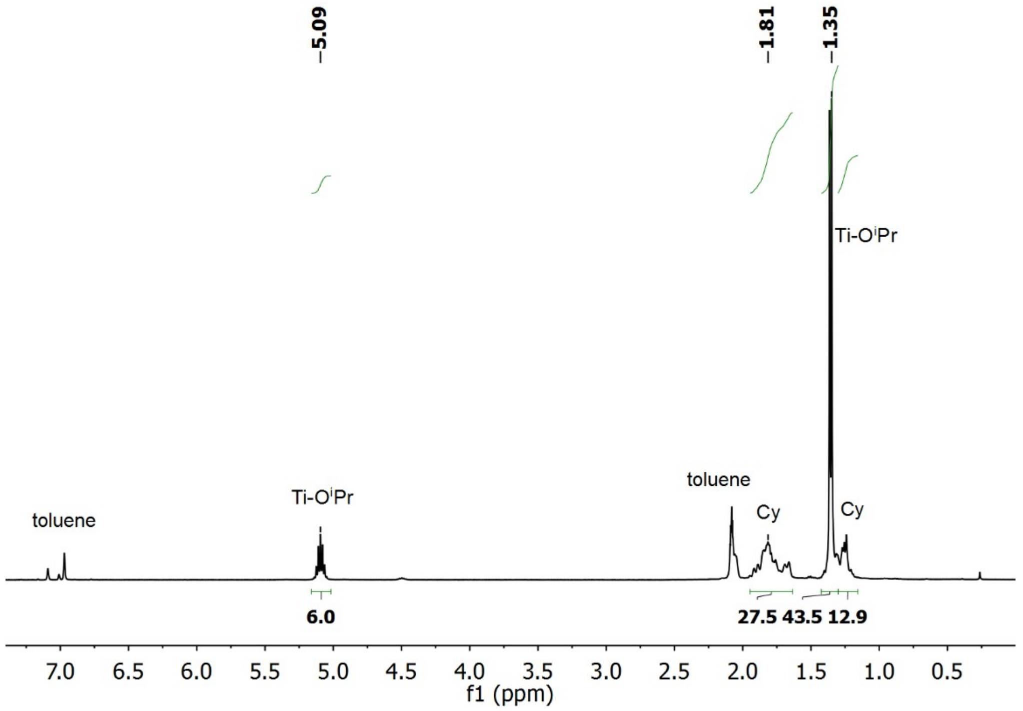

Figure S2. ^{1}H NMR spectrum for **1** (d^8-toluene, 400 MHz)

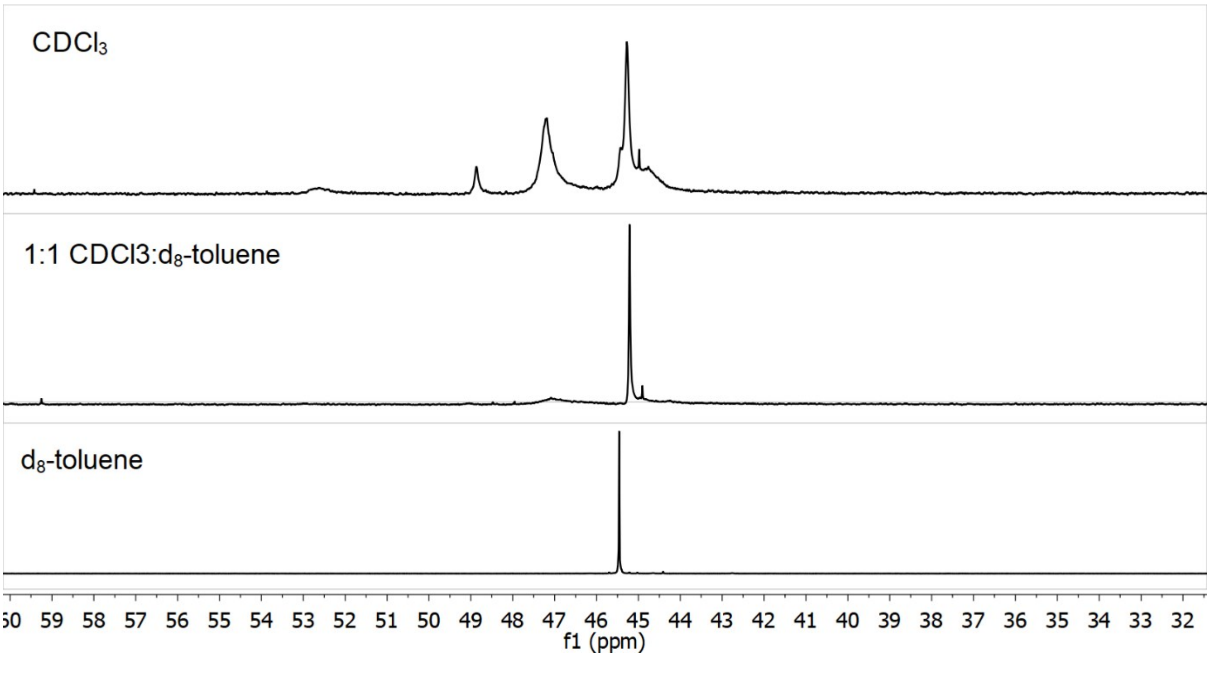

Figure S3. ^{31}P {^{1}H} NMR spectra for **1** in different solvents. A sample of **1** was first dissolved in $CDCl_3$, then the solvent was removed under vacuum and replaced by d_8-toluene, finally $CDCl_3$ was added to create a 1:1 mixture of the solvents.

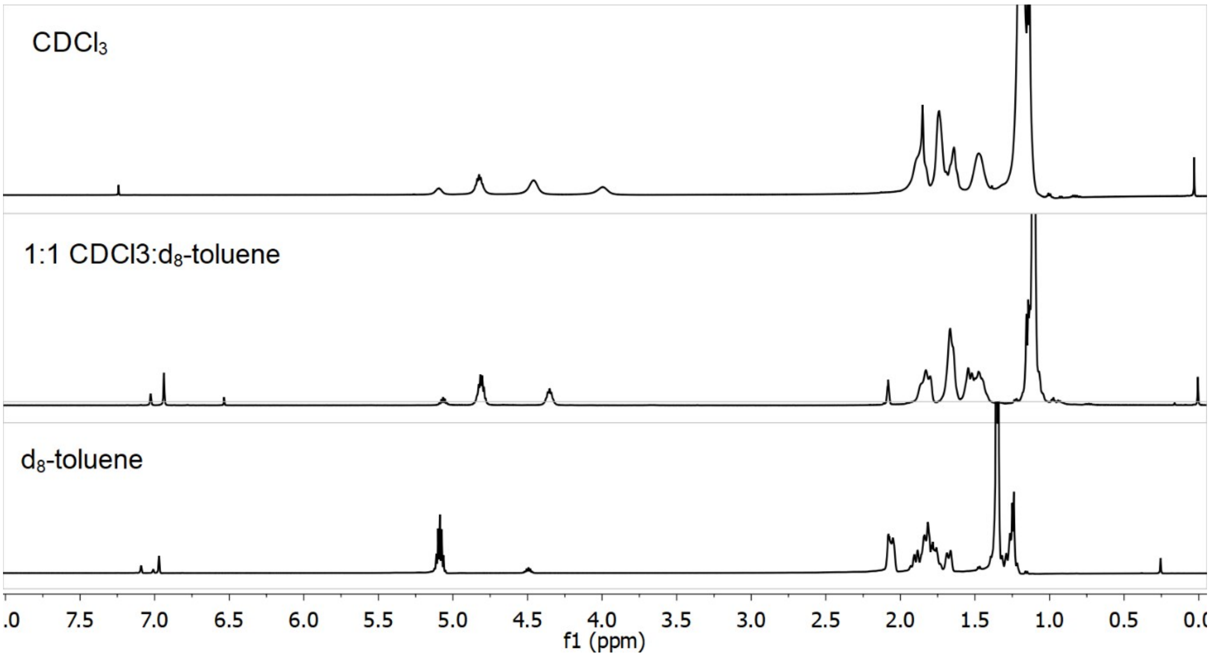

Figure S4. ^{1}H NMR spectra for **1** in different solvents. A sample of **1** was first dissolved in $CDCl_3$, then the solvent was removed under vacuum and replaced by d_8-toluene, finally $CDCl_3$ was added to create a 1:1 mixture of the solvents. Mixed solvent spectra locked and referenced to toluene.

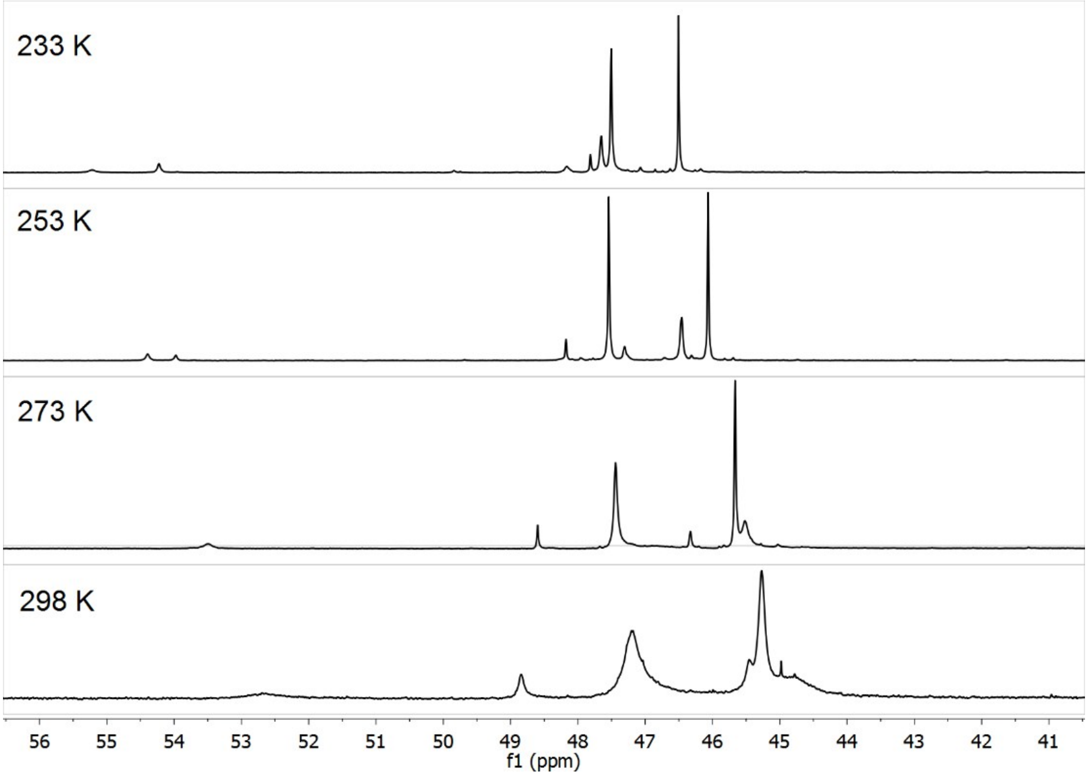

Figure S5. Variable temperature ^{31}P {^{1}H} NMR spectrum for **1** ($CDCl_3$, 162 MHz)

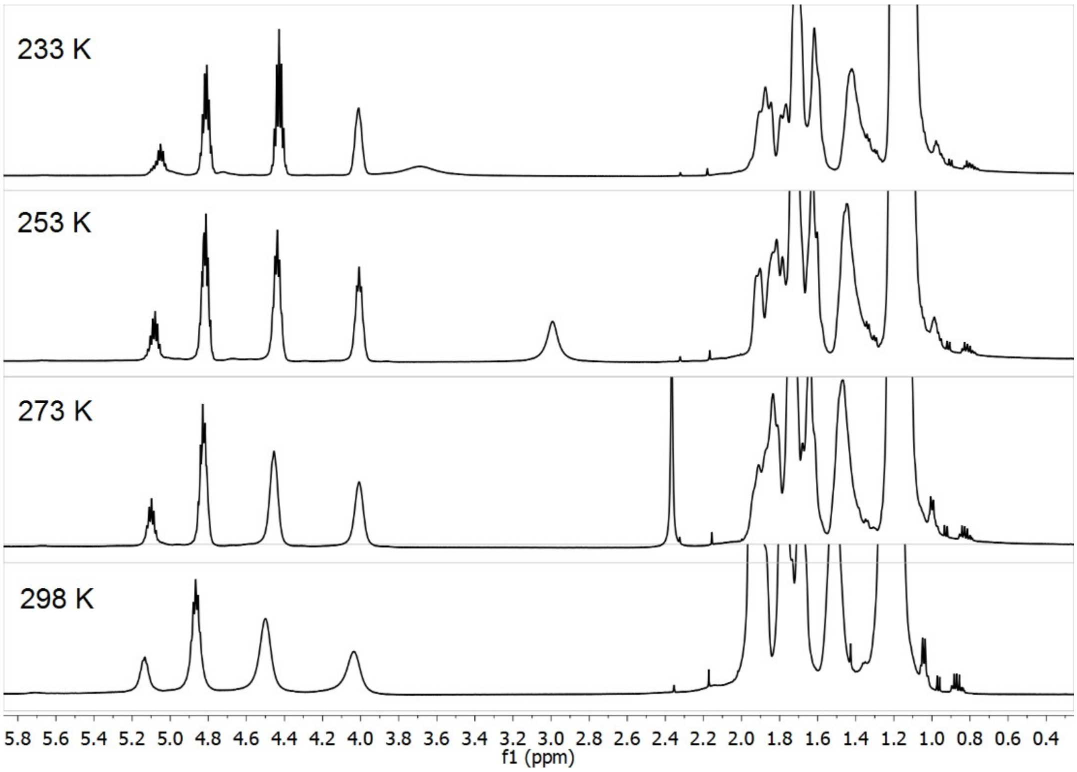

Figure S6. Variable temperature ^{1}H NMR spectrum for **1** ($CDCl_3$, 400 MHz)

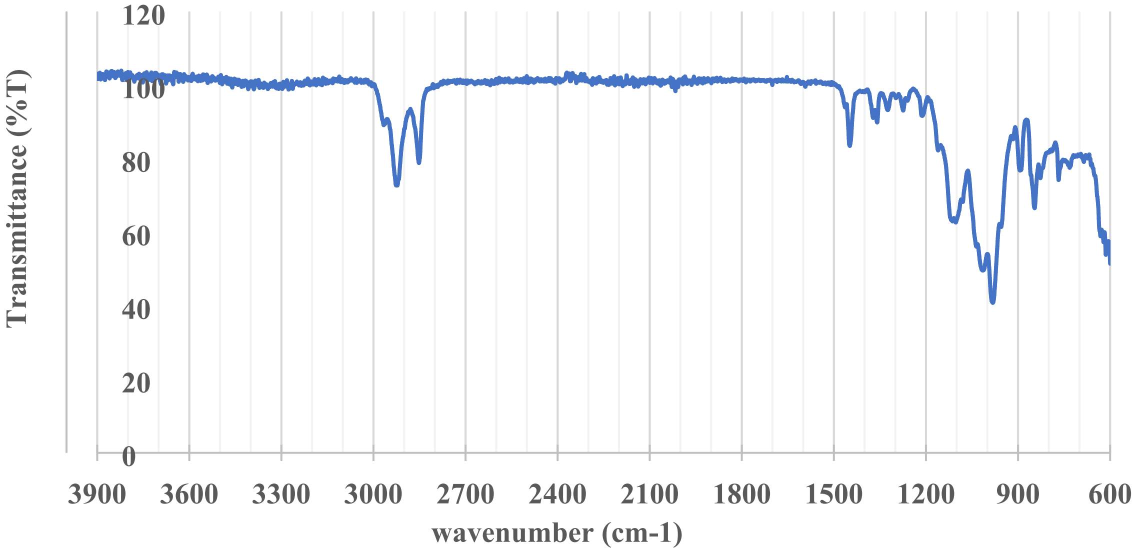

Figure S7. ATR-FTIR spectrum for **1**.

Characterisation for 2. $[Ti(O^iPr)_2(Ph_2PO_2)_2]_x$ (expected x = 6)

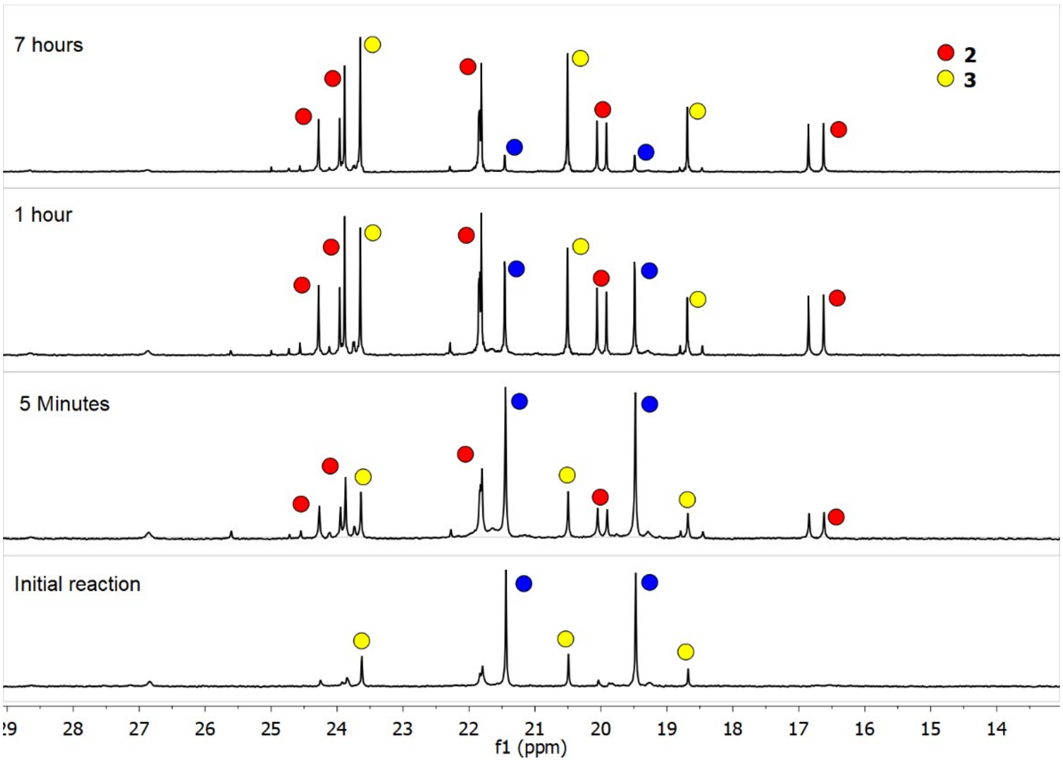

Figure S8. $^{31}P\ \{^1H\}$ NMR spectrum (d_8-toluene) of the reaction of 1:1 reaction of TiO^iPr_4 + Ph_2PO_2H over time. The reaction initially forms a species which shows two equivalent signals in its ^{31}P NMR spectrum (at 19.5 and 21.5 ppm), before evolving into **2** (and impurity **3**). Blue dots represent initial species that forms, red dots represent complex **2**. Yellow dots represent complex **3** as an impurity.

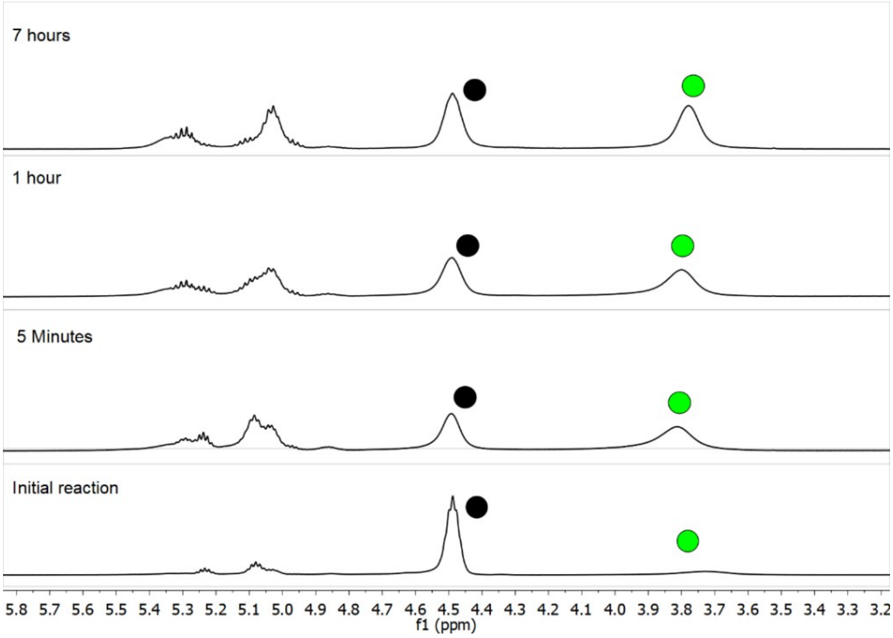

Figure S9. ^1H NMR (d_8-toluene) spectrum of the reaction of 1:1 reaction of TiO^iPr_4 + Ph_2PO_2H over time. Black dots represent starting material TiO^iPr_4, green dots represent released HO^iPr. The other species between 5.0-5.5 ppm are $Ti\text{-}O^iPr$ groups on **2** (and impurity **3**)

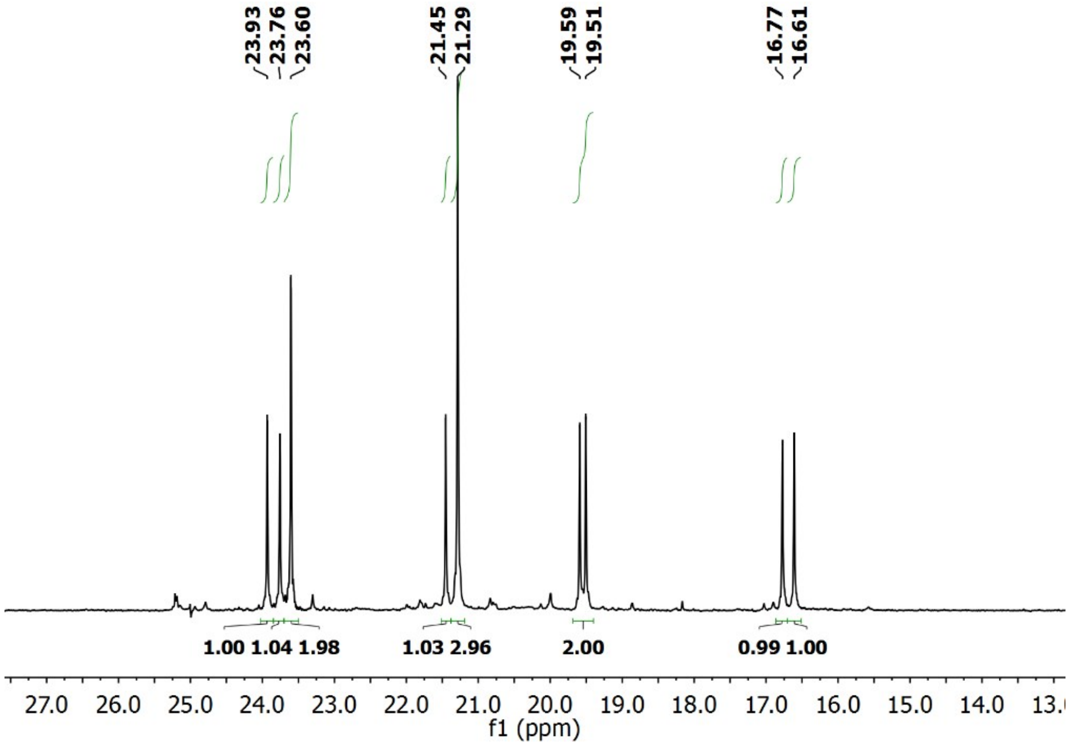

Figure S10. $^{31}P\{^1H\}$ NMR spectrum of **2** ($CDCl_3$, 162 MHz)

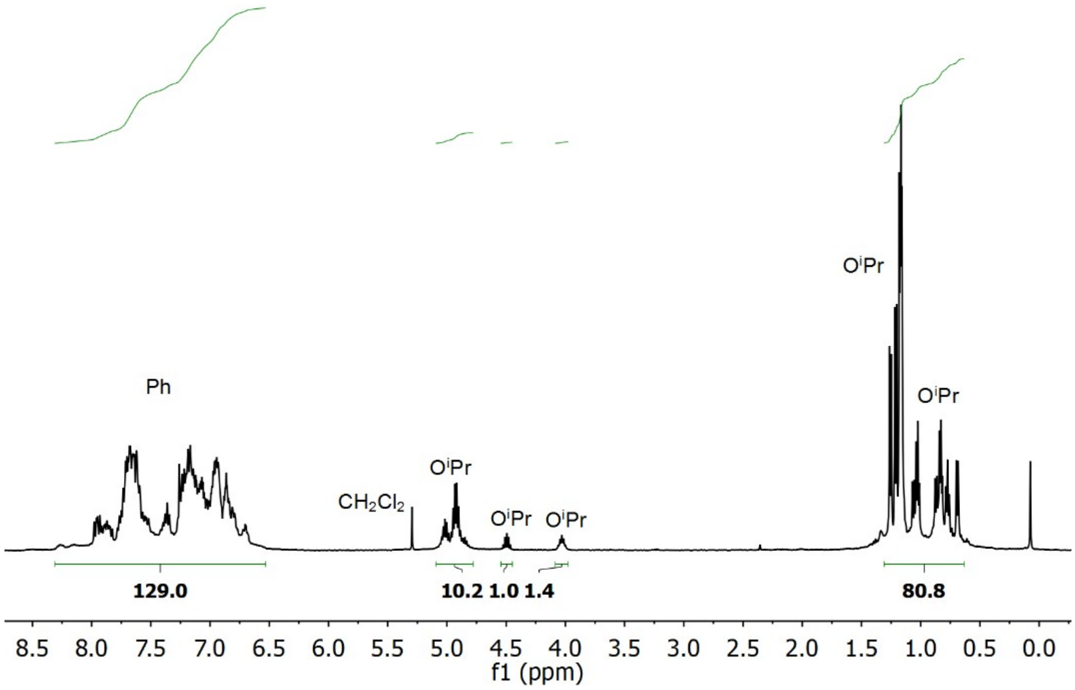

Figure S11. ^1H NMR spectrum of **2** ($CDCl_3$, 400 MHz)

Analysis of ^1H COSY NMR spectra reveal that three OC**H**Me_2 protons (4.85. 4.49 & 4.03) couple to symmetrical Me groups (1.25, 1.21, 0.69), three OC**H**Me_2 protons (5.02) couple to asymmetrical Me groups (1.08-1, 0.89-0.81), the remaining six OC**H**Me_2 protons (4.92) couple to signals at both 1.19-1.14 and 0.79-0.75 likely a combination of symmetrical and asymmetrical Me environments.

Characterisation for 3. $Ti_3(O^iPr)_7(Ph_2PO_2)_5$

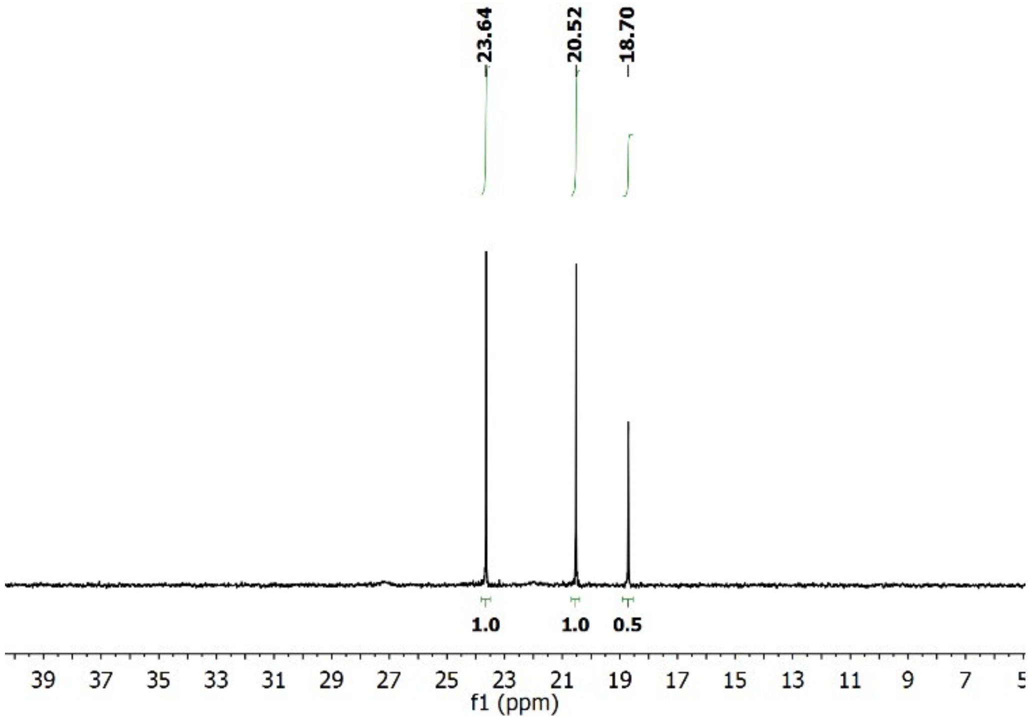

Figure S12. $^{31}P\ \{^1H\}$ NMR spectrum of **3** (d^8-toluene, 162 MHz)

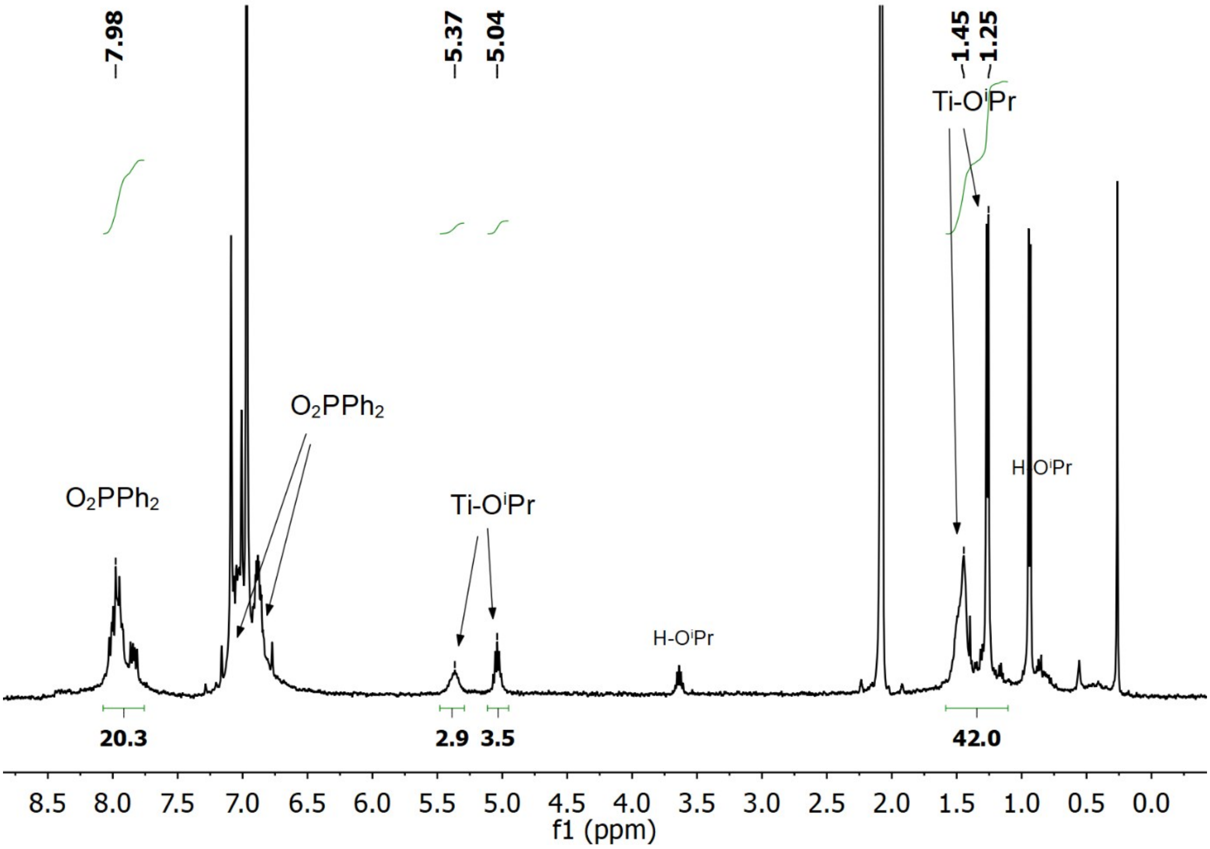

Figure S13. ^1H NMR spectrum of **3** (d^8-toluene, 400 MHz)

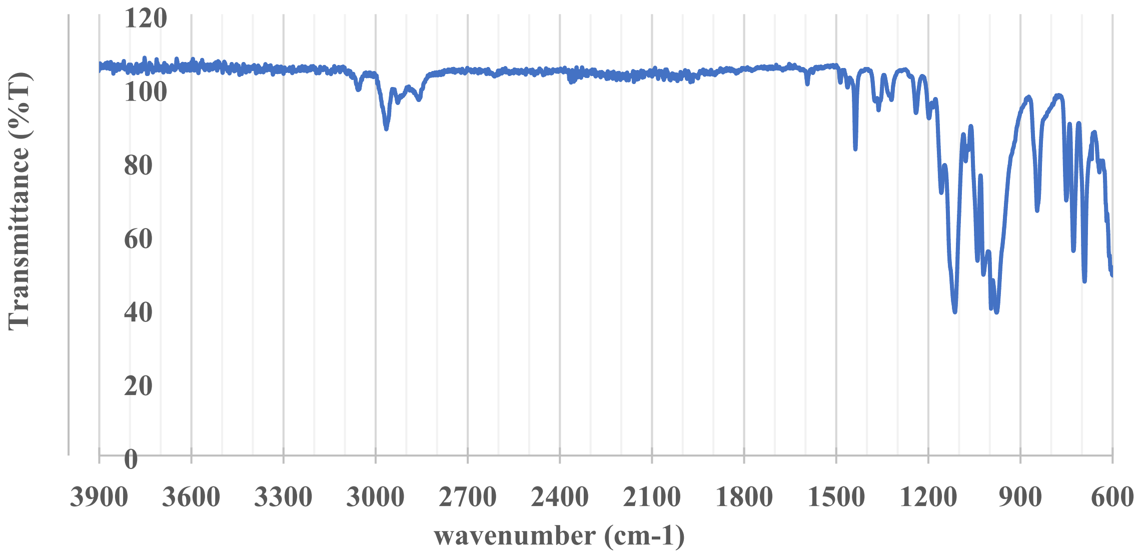

Figure S14. ATR-FTIR spectrum for **3**.

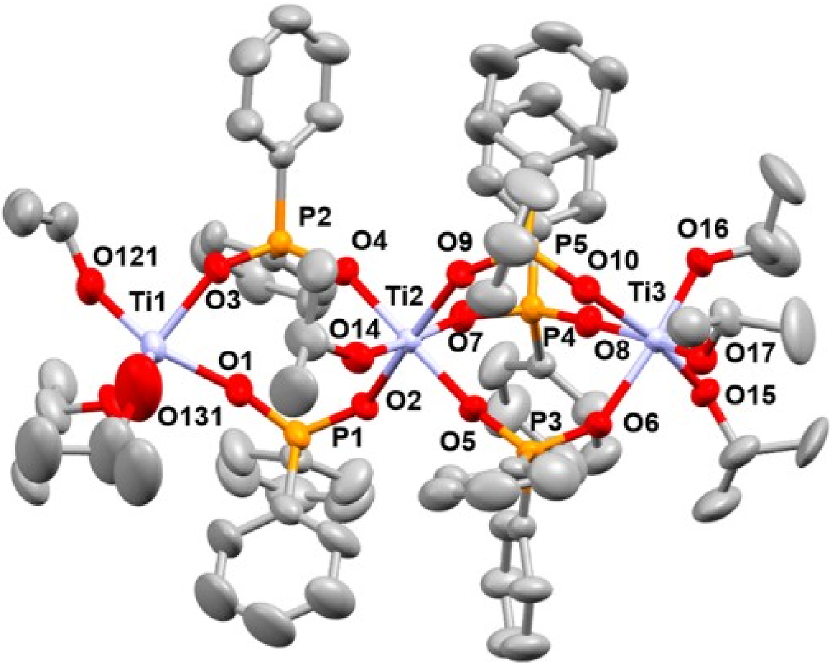

Figure S15. Solid state structure of **3**, hydrogen atoms omitted for clarity. Ellipsoids displayed at 50% probability. Selected bond lengths (Å) and angles (°): Ti–OPOPh$_2$ range, 1.924(4)-2.107(4); Ti2–O14, 1.762(4); Ti3-O^{i}Pr, 1.826(4)-1.832(4); P–O range, 1.491(4)-1.518(4); O121-Ti1-O1, 150.4(3); O1-Ti1-O3, 80.62(16), O4-Ti2-O14, 90.32(17), O10-Ti3-O8, 81.92(15), O6-Ti3-O16, 174.54(17)

Characterisation for 4. [TiO(O^{i}Pr)(Cy_2PO_2)]$_4$

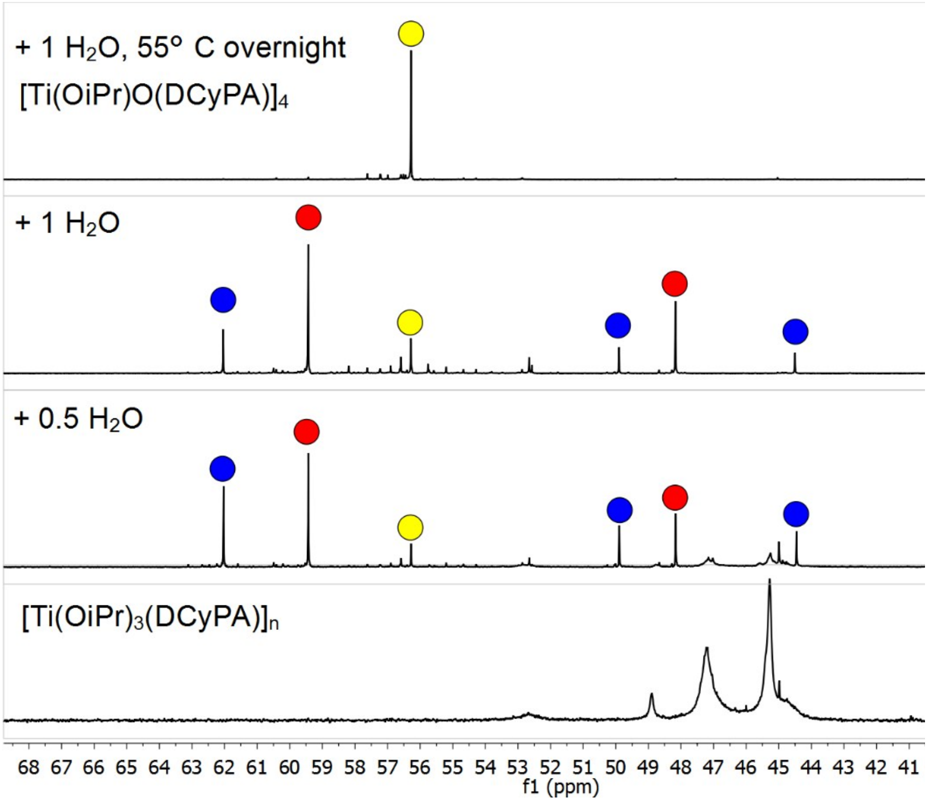

Figure S16. ^{31}P {^1H} NMR spectra ($CDCl_3$) of the reaction of **1** with water, followed by heating at 55°C overnight. The reaction of **1** with water was followed by in-situ ^{31}P NMR spectroscopy revealing a variety of ^{31}P signals from partially hydrolysed complexes. The blue and red dots are for unknown intermediary species, the yellow dots are for complex **4**. Note that the initially broad peaks for **1** are consistent with a dimer-oligomer mixture, which is observed in $CDCl_3$, see Figure S3.

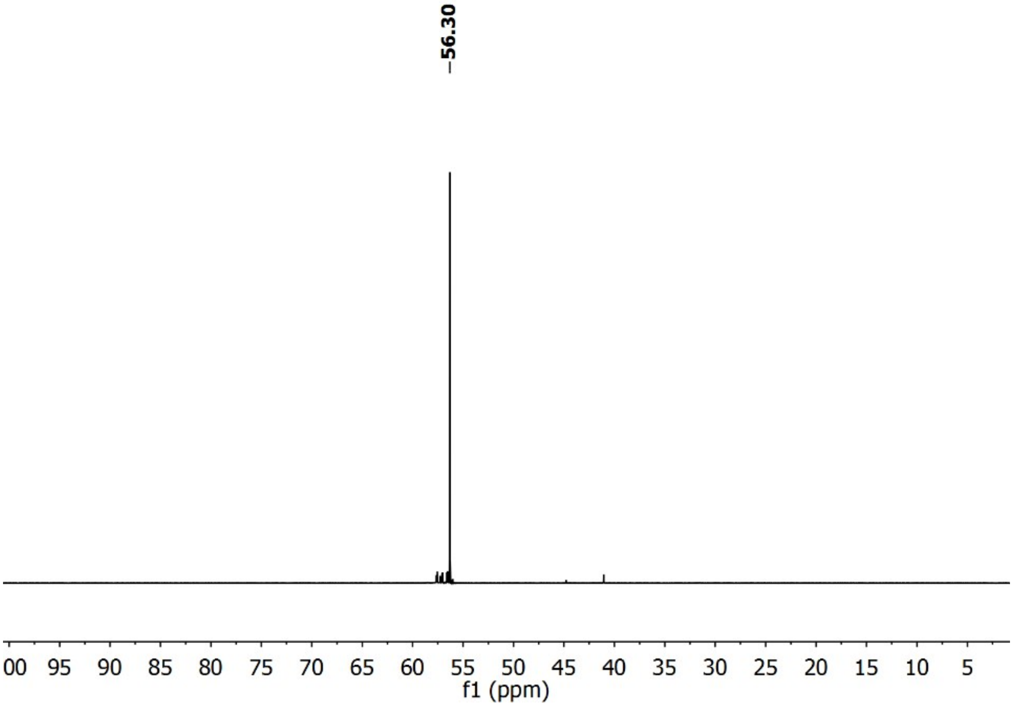

Figure S17. ^{31}P {^1H} NMR spectrum of **4** ($CDCl_3$, 162 MHz)

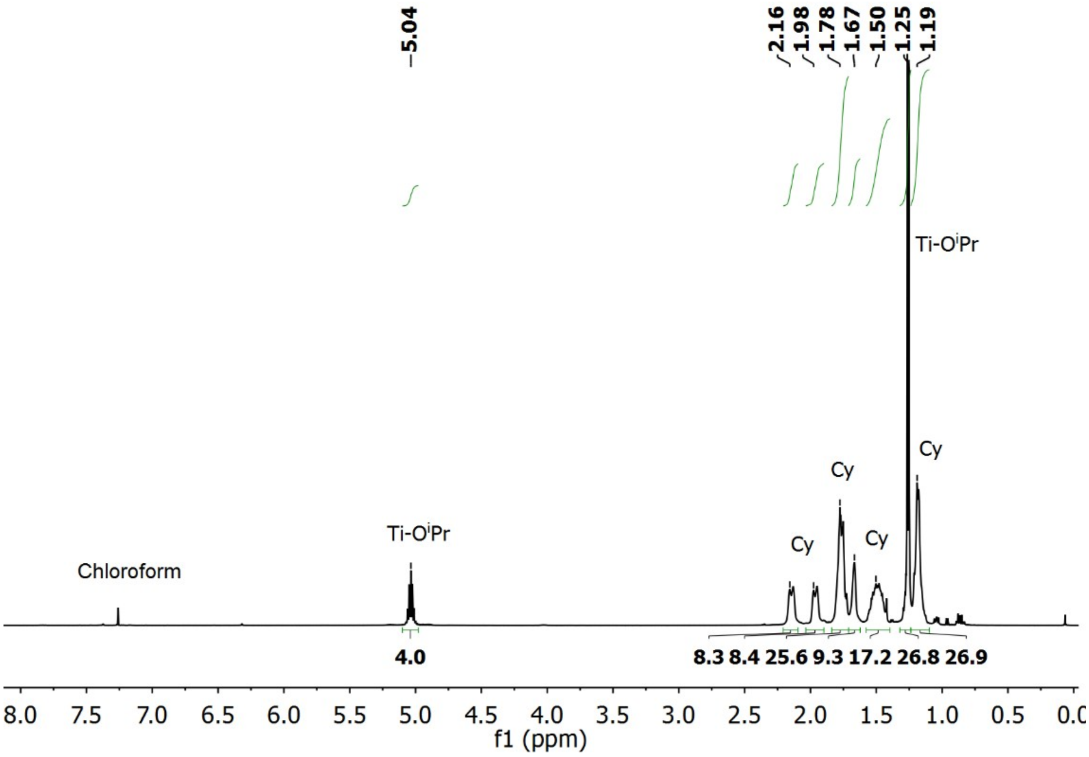

Figure S18. ^{1}H NMR spectrum of **4** ($CDCl_3$, 400 MHz)

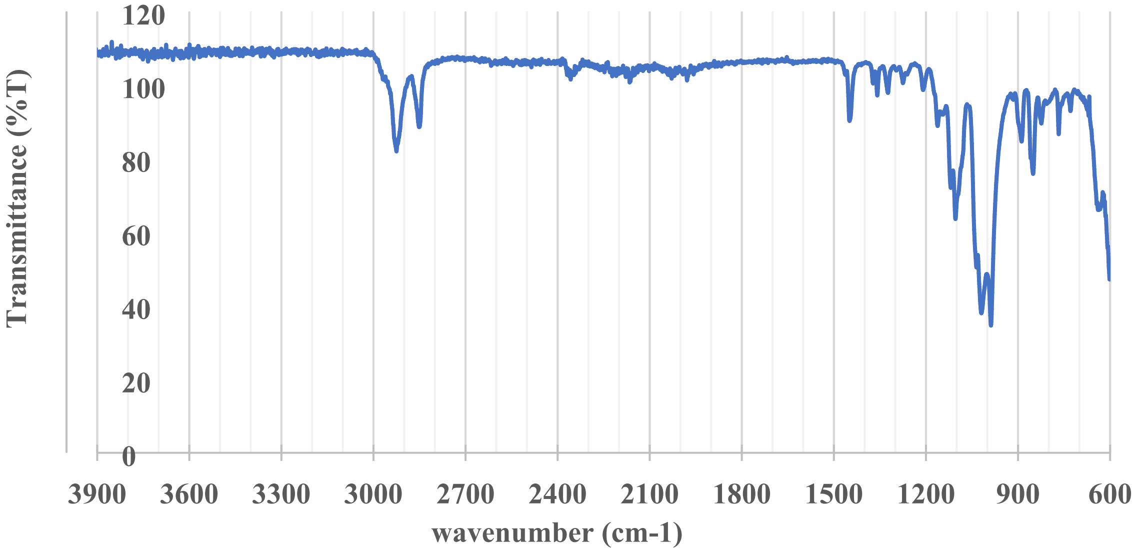

Figure S19. ATR-FTIR spectrum for **4**.

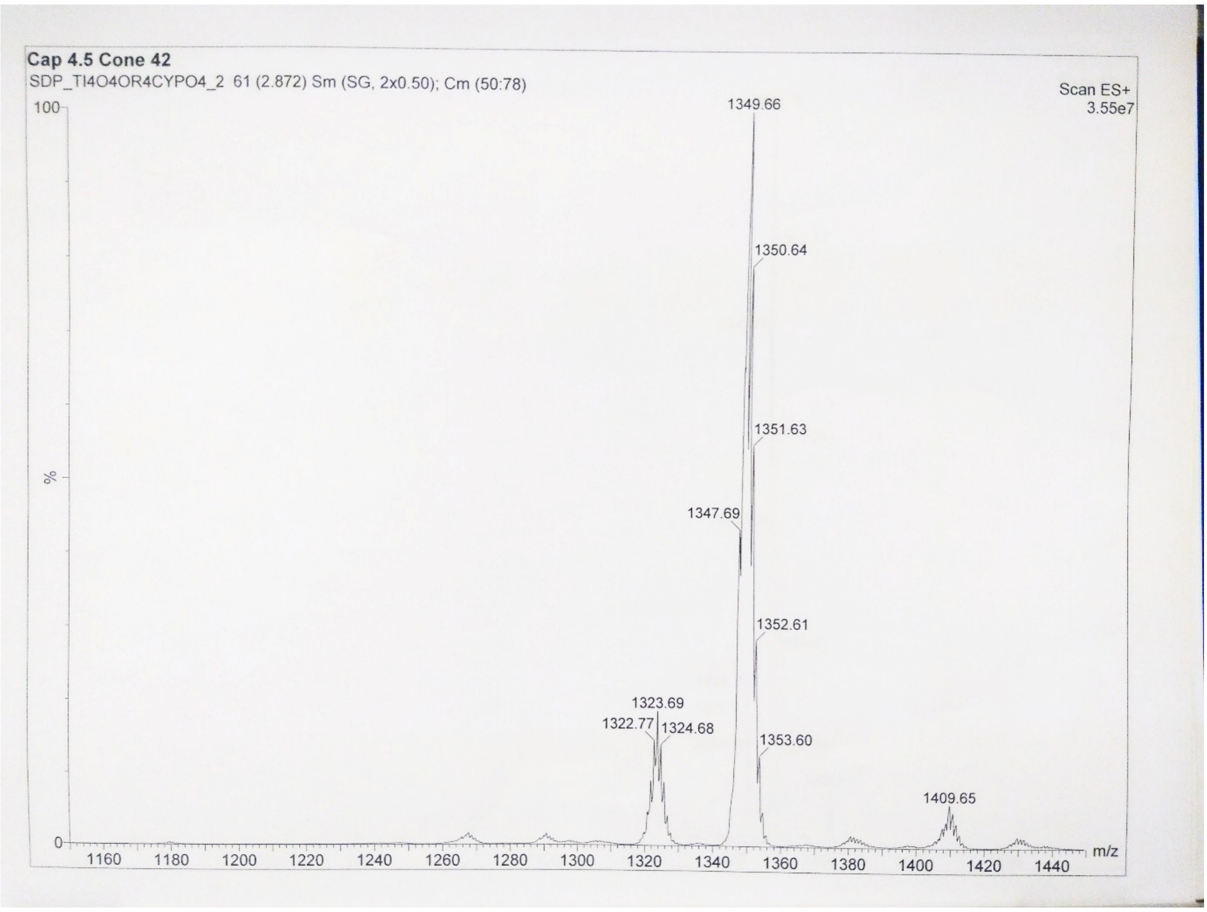

Figure S20. ESI Mass spectrum of **4** (CH_2Cl_2). Main features for [**4**-H]$^+$ and for $[Ti_4O_4(O^iPr)_3(Cy_2PO_2)_4]^+$

Characterisation for 5. $[TiO(O^iPr)(Ph_2PO_2)]_4.C_6H_5Me$

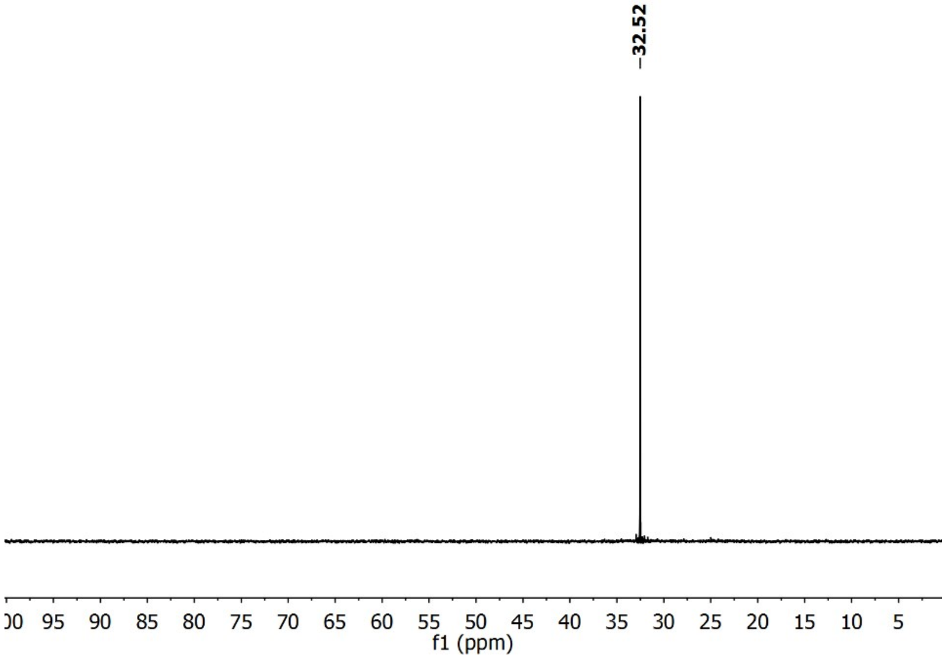

Figure S21. $^{31}P\{^1H\}$ NMR spectrum of **5** (d^8-toluene, 162 MHz)

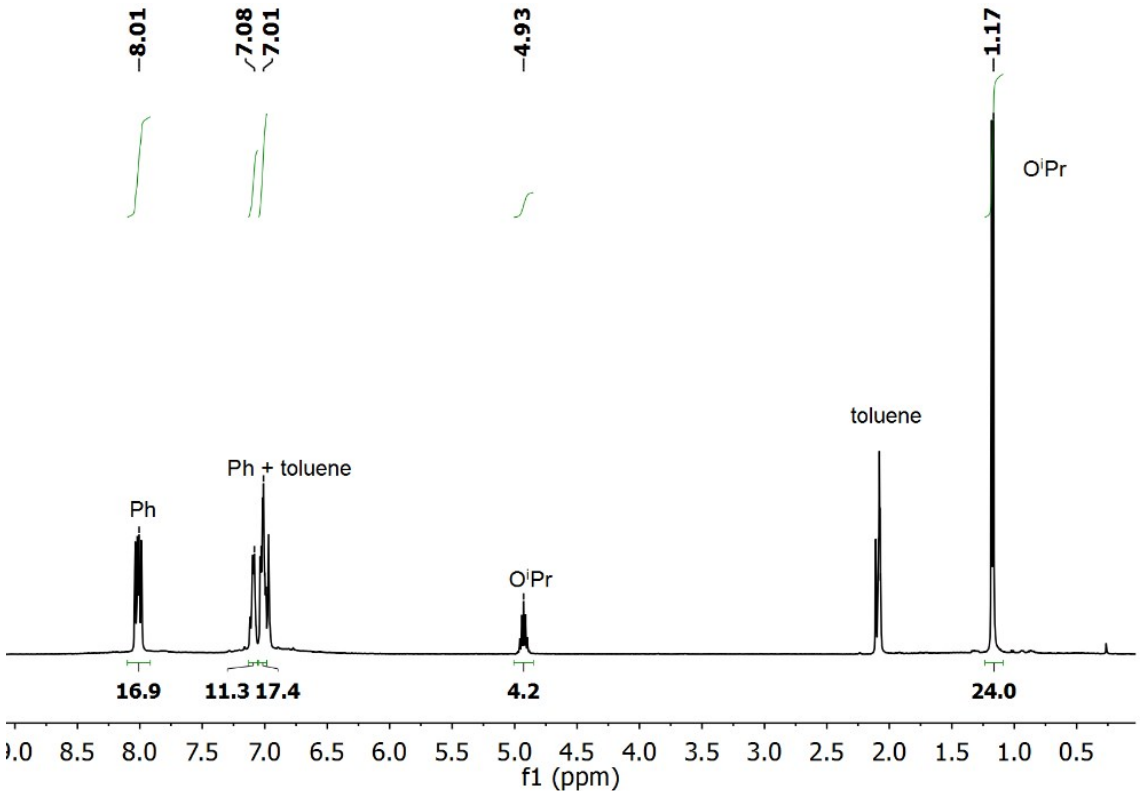

Figure S22. ^{1}H NMR spectrum of **5** (d^8-toluene, 400 MHz)

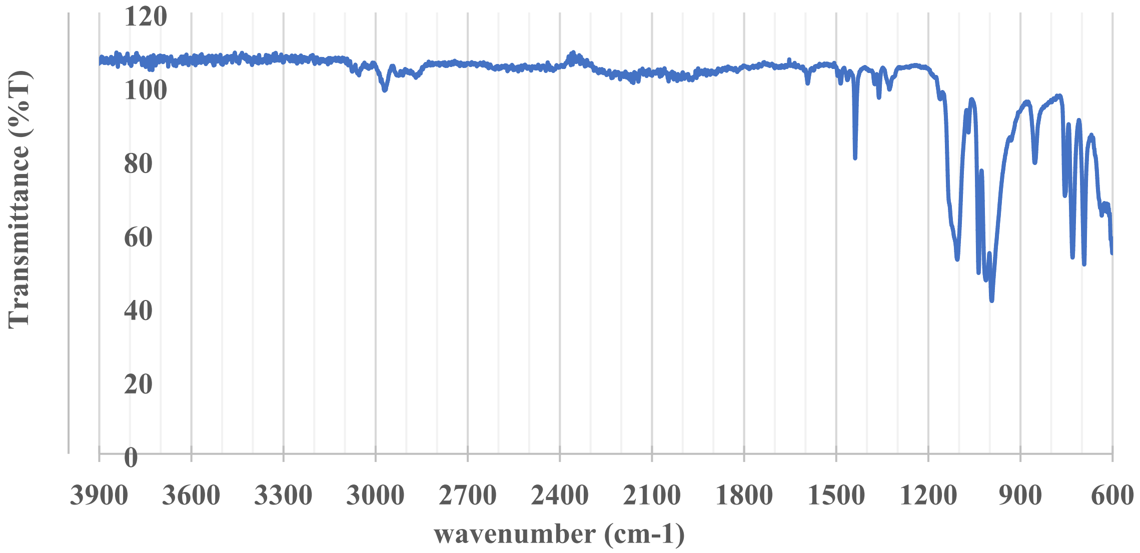

Figure S23. ATR-FTIR spectrum for **5**.

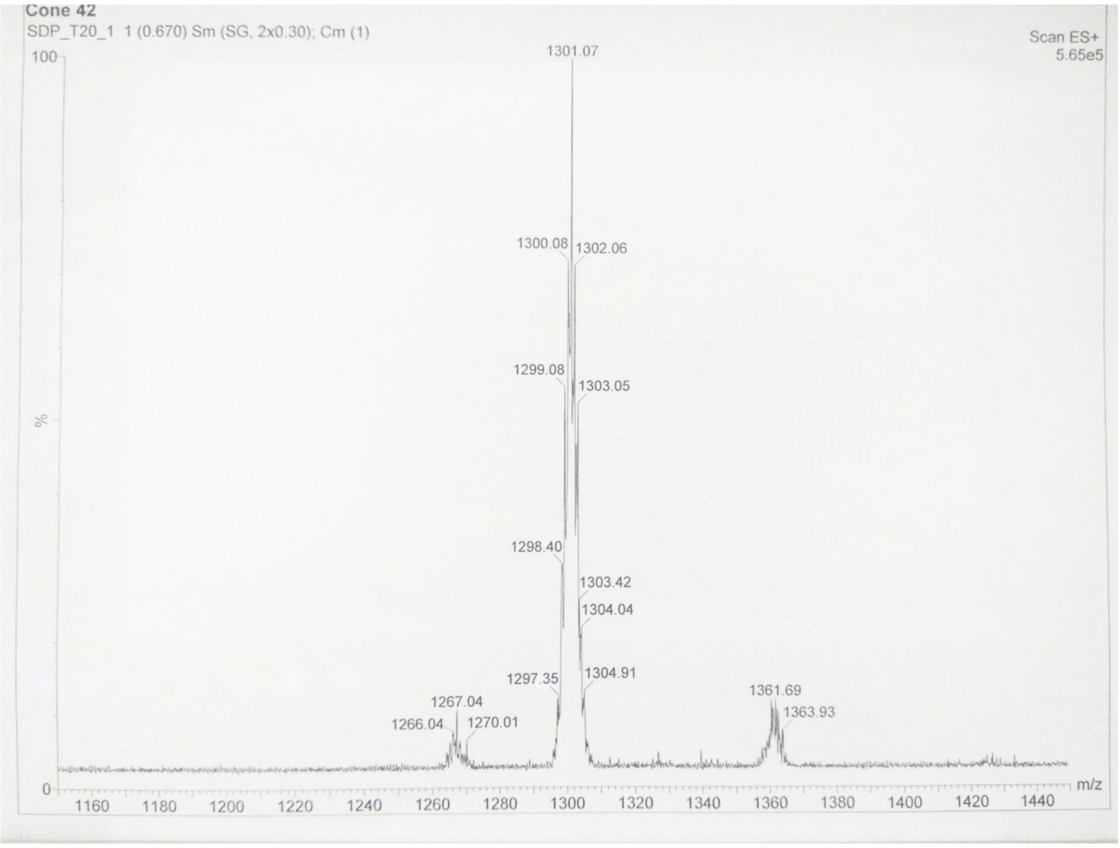

Figure S24. ESI Mass spectrum of **5** (CH_2Cl_2). Main features for [**5**-H]$^+$ and for $[Ti_4O_4(O^iPr)_3(Cy_2PO_2)_4]^+$

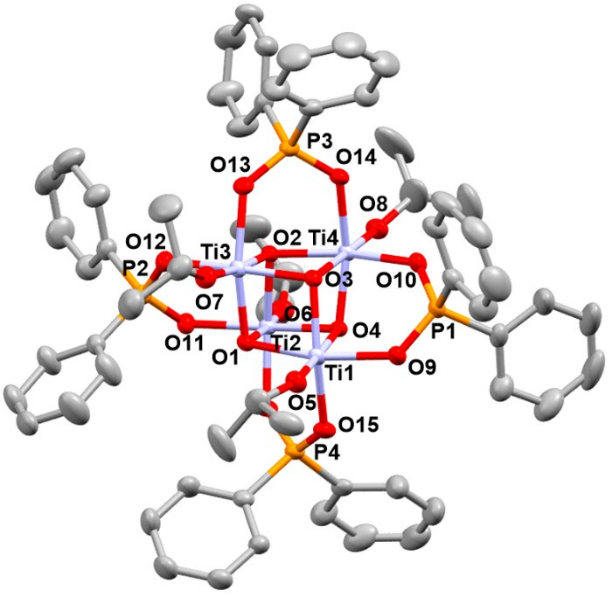

Figure S25. Solid state structure of **5**·toluene, toluene molecule and hydrogen atoms omitted for clarity. Ellipsoids displayed at 50% probability. Selected bond lengths (Å) and angles (°): Ti−Oxo, each Ti forms two shorter bonds to oxo, range 1.9083(19)-1.9448(18), and one longer bond, range 2.121(2)-2.159(2); Ti−$OPOPh_2$ range, 2.014(2)-2.057(2); Ti-O^iPr range, 1.773(2)-1.794(2); P−O range 1.522(2)-1.529(2); Ti1···Ti3, 2.9139(7); Ti2···Ti4, 2.9116(7); O-P-O range, 114.69(11)-115.19(11); O1-Ti1-O3, 80.59(8); O1-Ti1-O4, 79.86(8); O1-Ti1-O5, 97.63(9); O1-Ti1-O9, 164.54(8), O1-Ti1-O15, 94.28(8)

UV Spectroscopy data

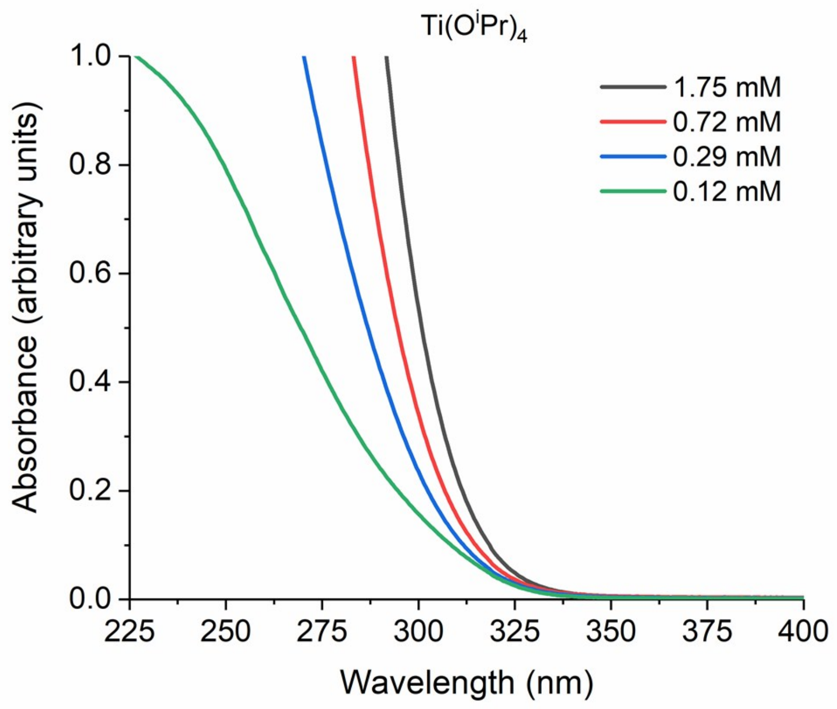

Figure S26. UV spectra of Ti(O^{i}Pr)$_4$ in pentane at different concentrations. A pentane only background was subtracted from the data.

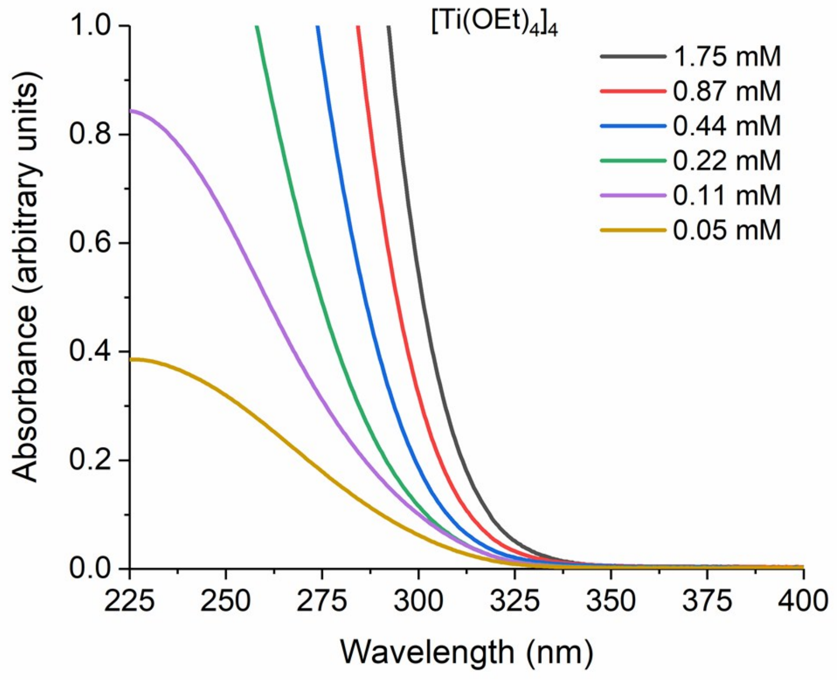

Figure S27. UV spectra of [Ti(OEt)$_4$]$_4$ in pentane at different concentrations. A pentane only background was subtracted from the data.

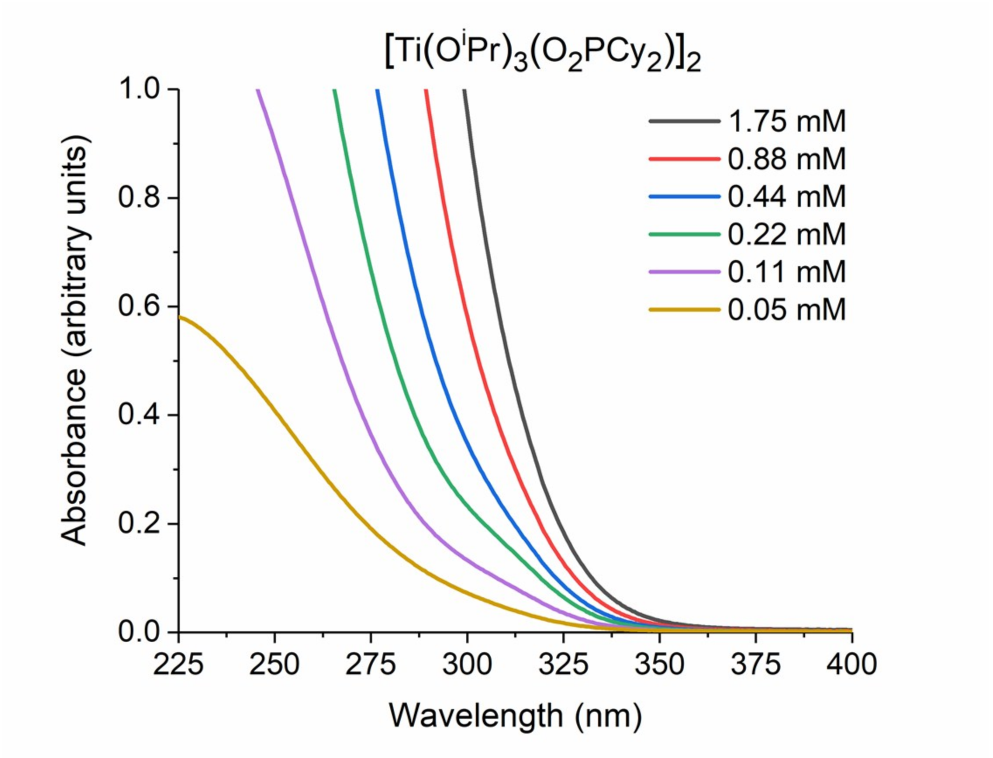

Figure S28. UV spectra of **1** in pentane at different concentrations. A pentane only background was subtracted from the data.

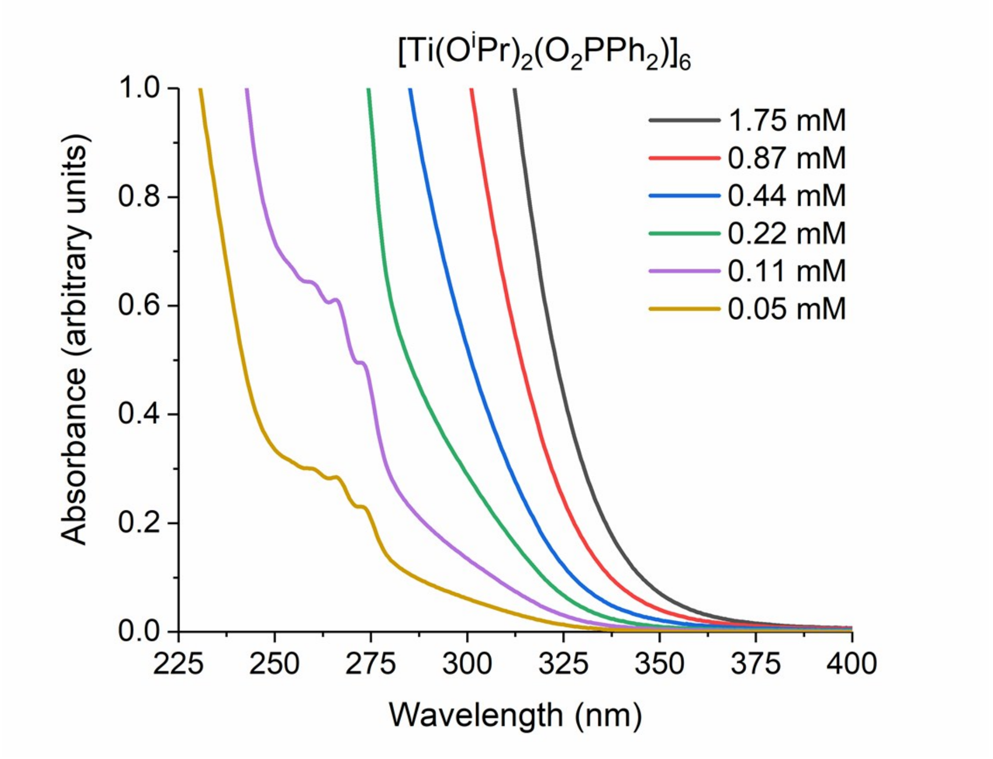

Figure S29. UV spectra of **2** in CH_2Cl_2 at different concentrations. A CH_2Cl_2 only background was subtracted from the data.

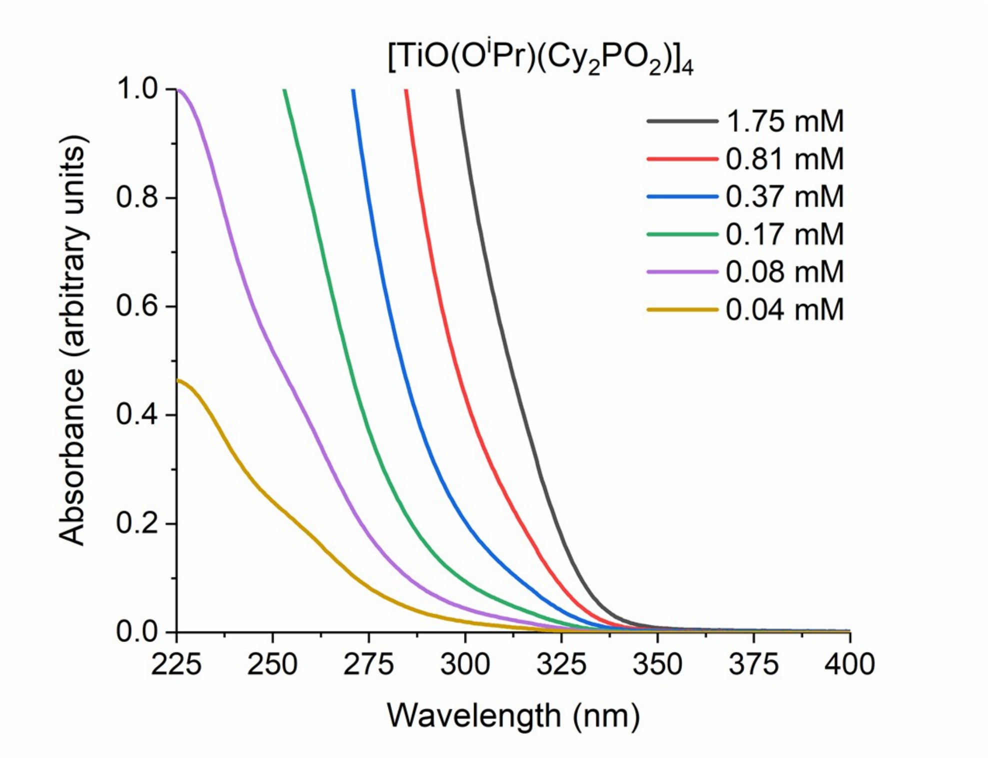

Figure S30. UV spectra of **4** in pentane at different concentrations. A pentane only background was subtracted from the data.

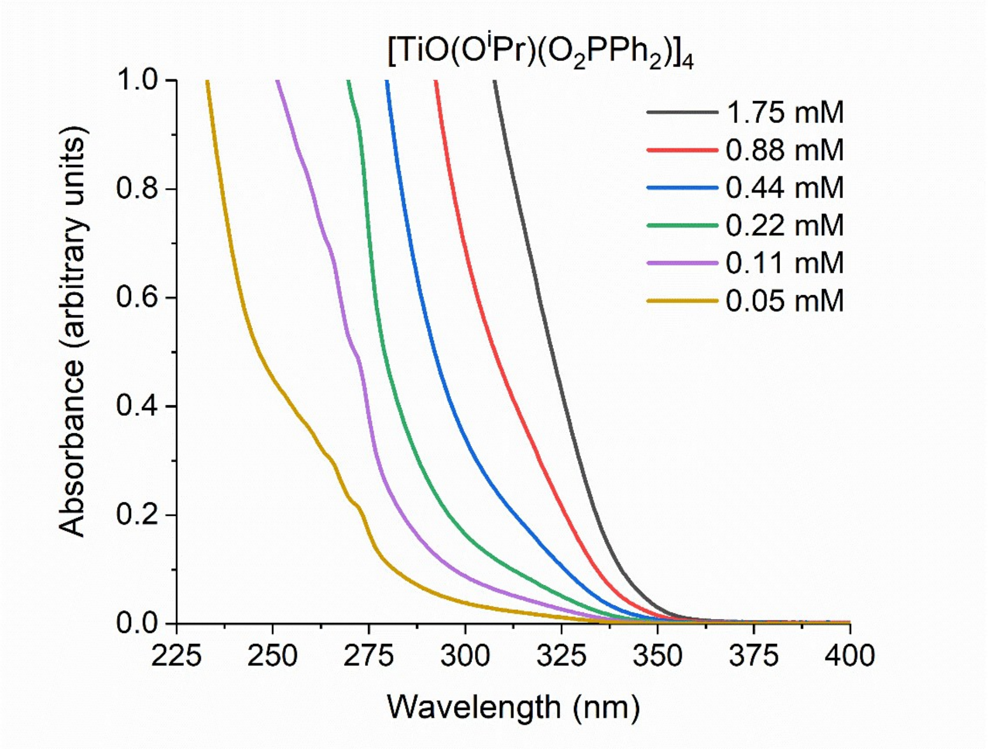

Figure S31. UV spectra of **5** in CH_2Cl_2 at different concentrations. A CH_2Cl_2 only background was subtracted from the data.

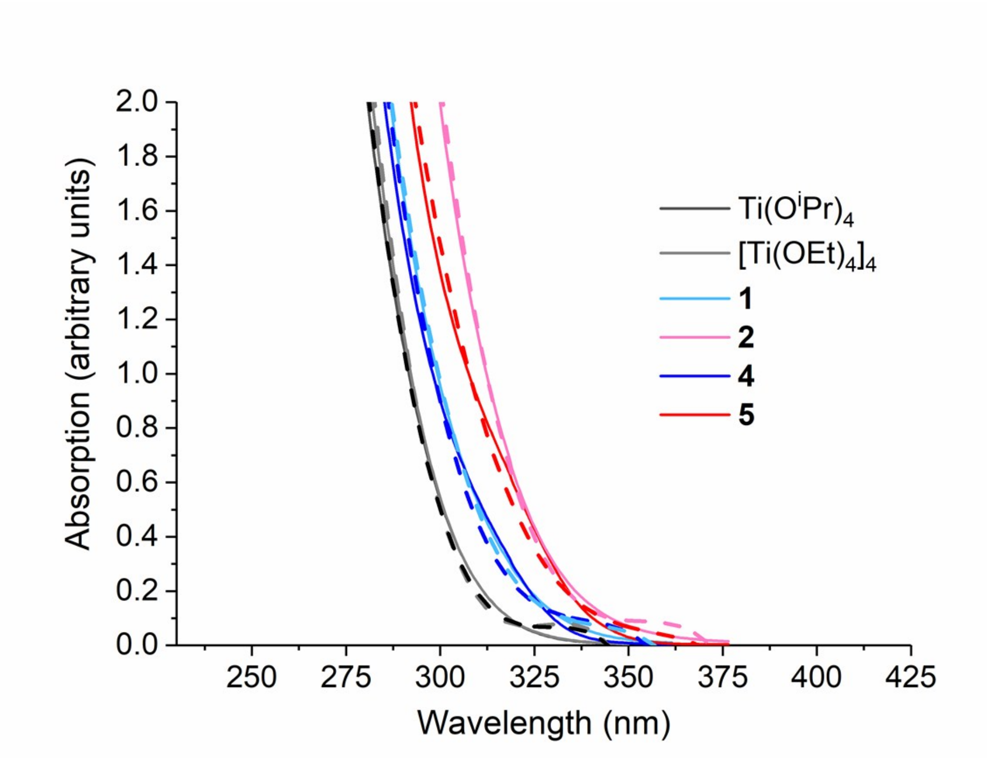

Figure 32. Solid lines, UV spectra at [Ti] = 1.75 mM; dashed lines, cubic fits. All fits give $r^2 > 0.99$ and are a much better match than quadratic fits.

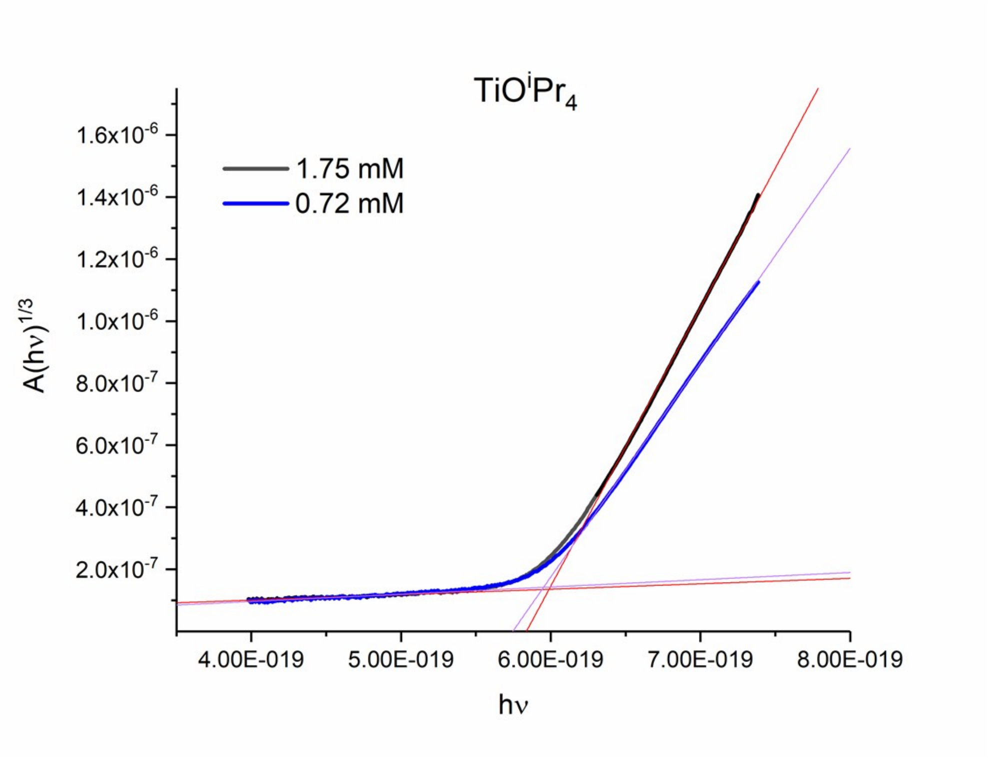

Figure S33. Tauc plot of absorption onset for Ti(O^{i}Pr)$_4$ at two different concentrations. X-value of intercept of linear section with linear baseline extrapolation used for value of absorption onset. Intercepts correspond to energies of 3.74 and 3.72 eV.

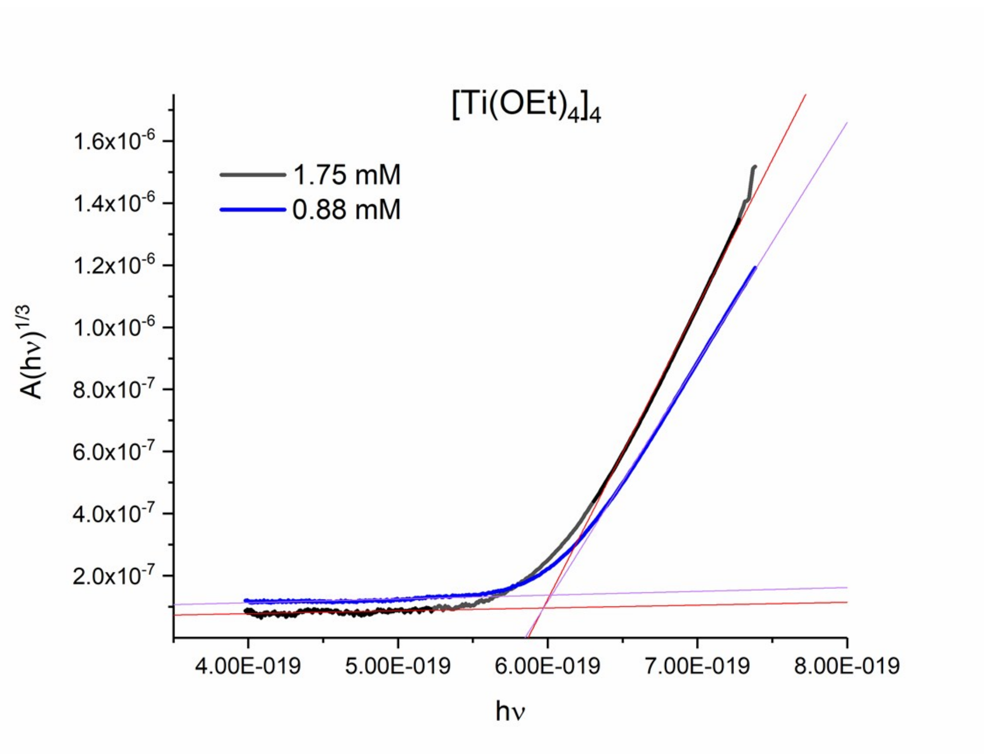

Figure S34. Tauc plot of absorption onset for $[Ti(OEt)_4]_4$ at two different concentrations. X-value of intercept of linear section with linear baseline extrapolation used for value of absorption onset. Intercepts correspond to energies of 3.73 and 3.76 eV.

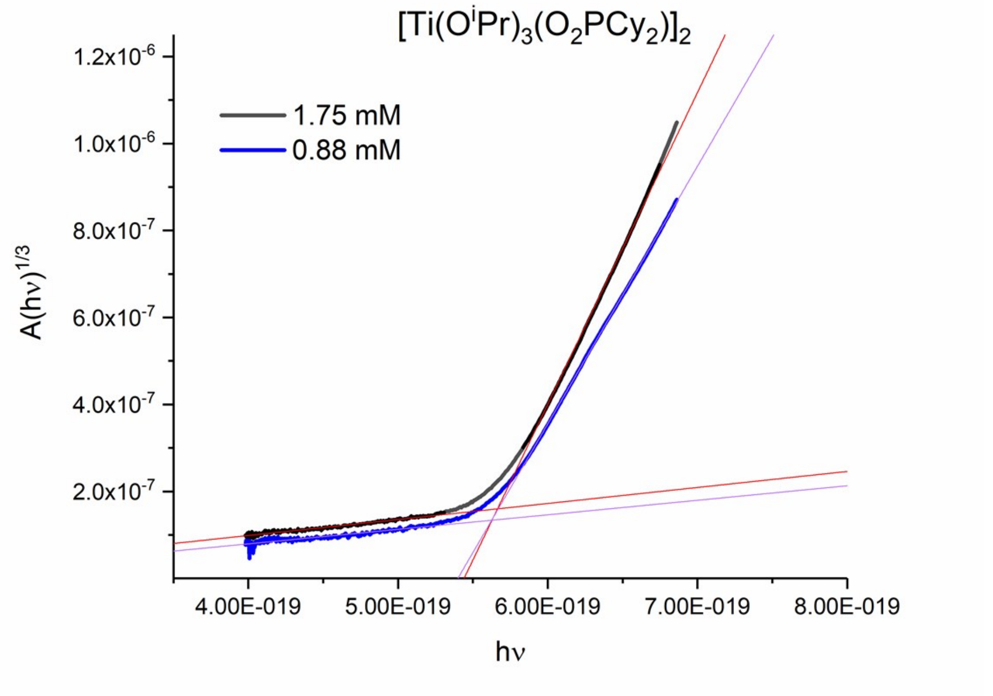

Figure S35. Tauc plot of absorption onset for **1** at two different concentrations. X-value of intercept of linear section with linear baseline extrapolation used for value of absorption onset. Intercepts correspond to energies of 3.53 and 3.52 eV.

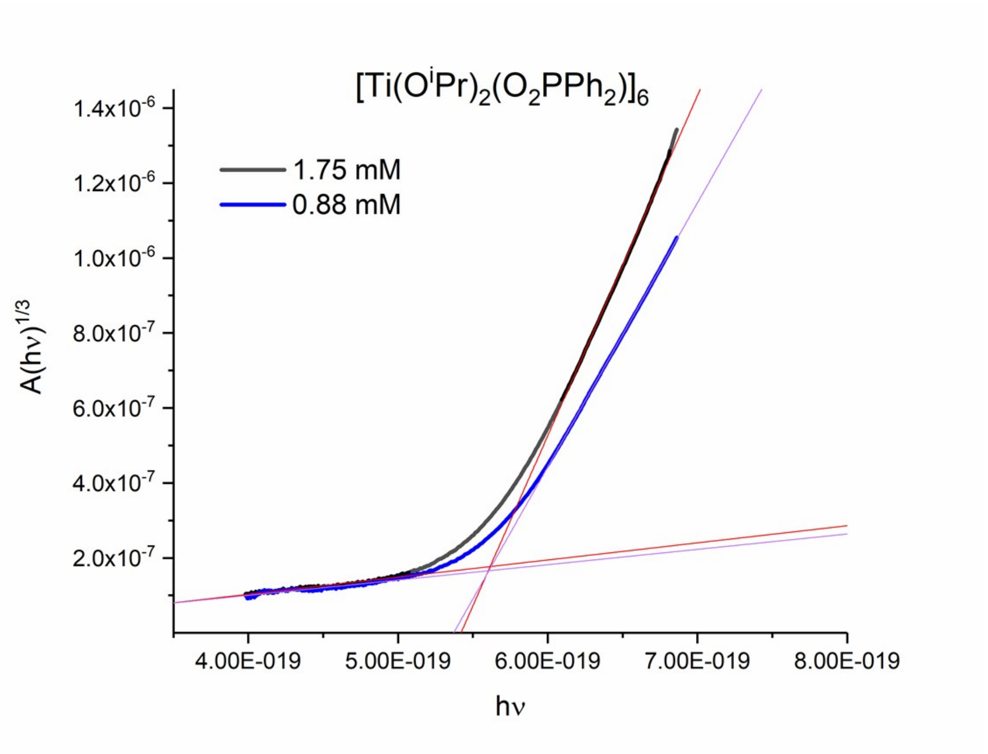

Figure S36. Tauc plot of absorption onset for **2** at two different concentrations. X-value of intercept of linear section with linear baseline extrapolation used for value of absorption onset. Intercepts correspond to energies of 3.50 and 3.49 eV.

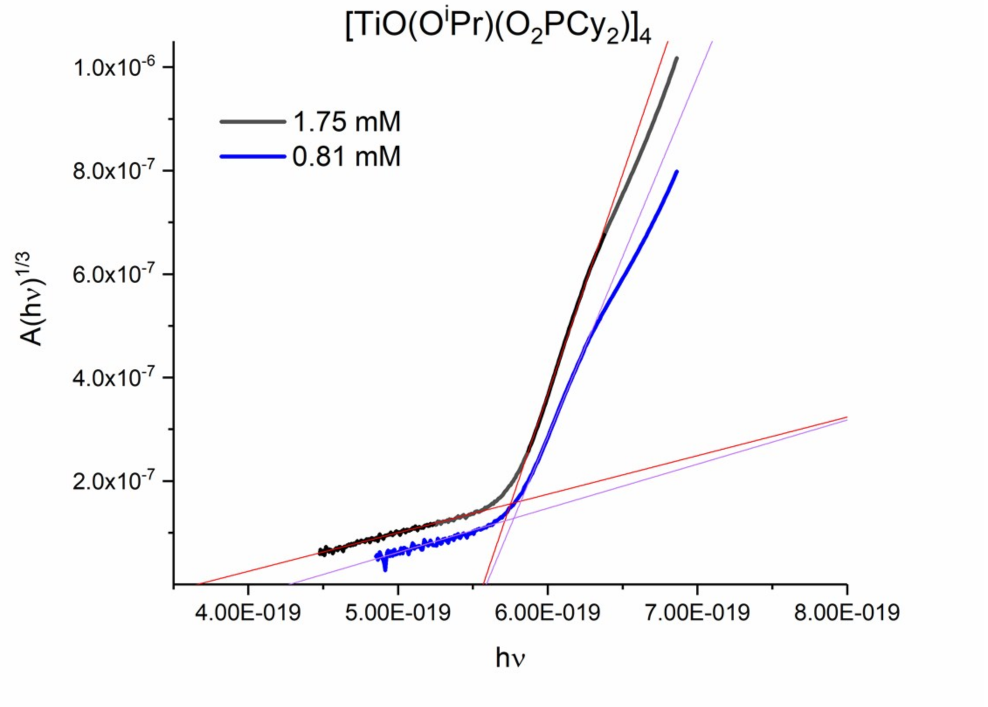

Figure S37. Tauc plot of absorption onset for **4** at two different concentrations. X-value of intercept of linear section with linear baseline extrapolation used for value of absorption onset. Intercepts correspond to energies of 3.59 and 3.60 eV.

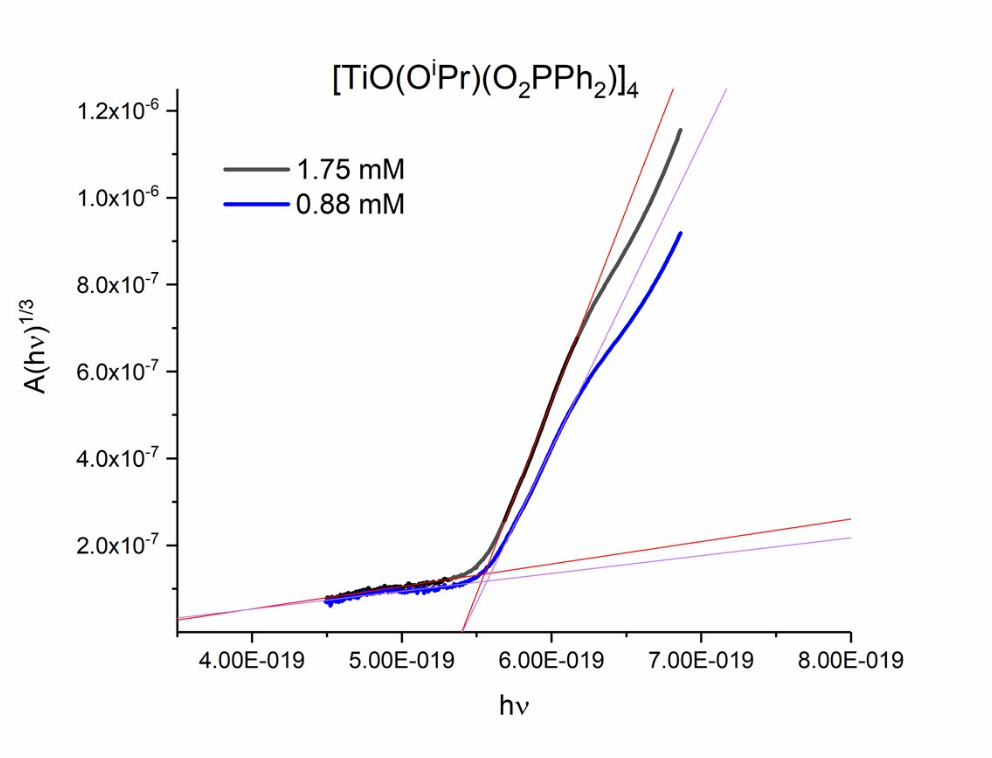

Figure S38. Tauc plot of absorption onset for **5** at two different concentrations. X-value of intercept of linear section with linear baseline extrapolation used for value of absorption onset. Intercepts correspond to energies of 3.47 and 3.48 eV.

DFT calculations for 4 and 5

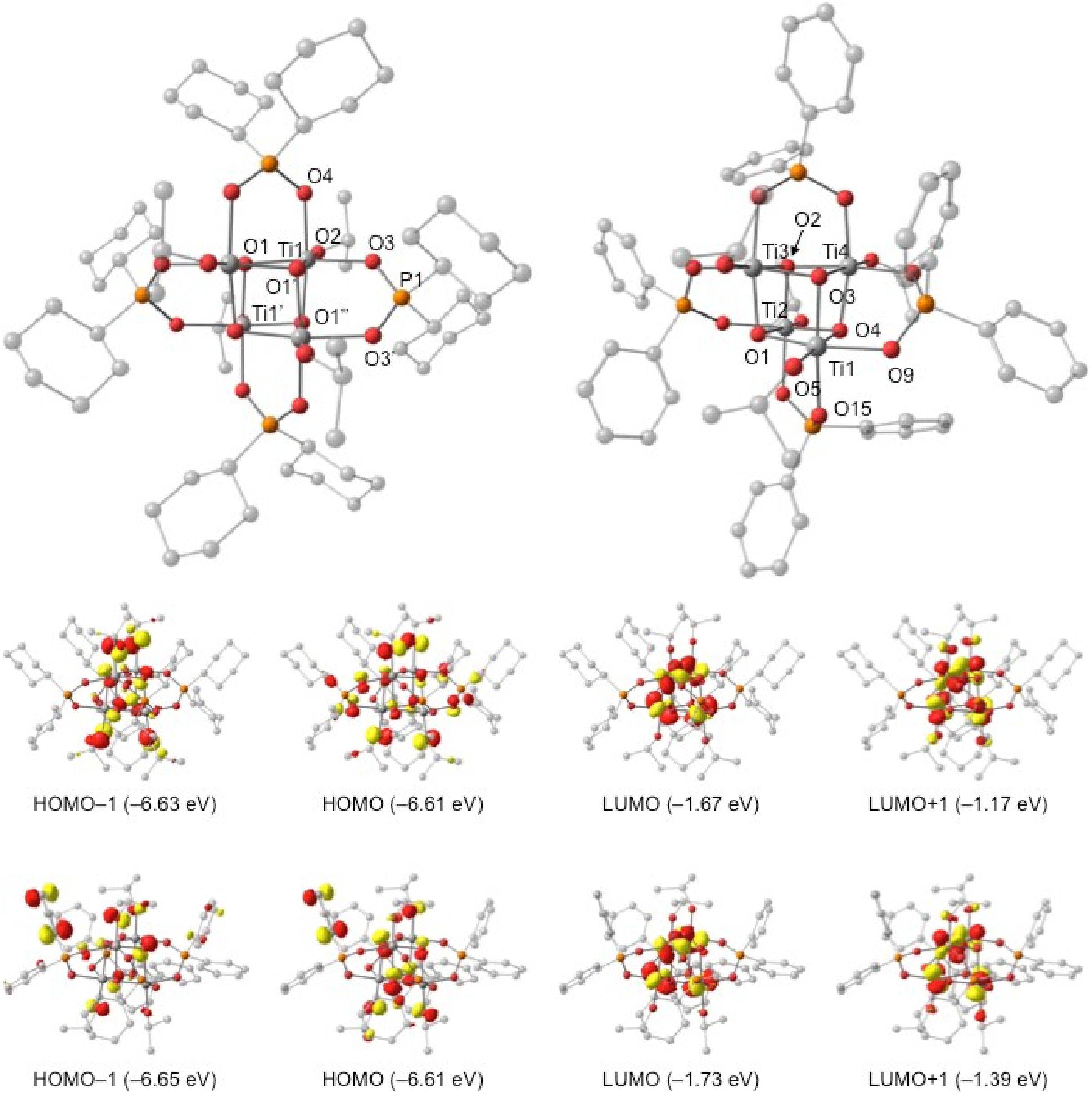

Figure S39. DFT-optimized geometries (B97-3c) of clusters **4** (top left) and **5** (top right) in their singlet (S = 0) ground states. Hydrogen atoms omitted for clarity. Below) Isosurface plots (cutoff = 0.05) of canonical Kohn-Sham frontier orbitals for **4** (middle row) and **5** (bottom row).

Table S1. Key DFT-optimized bond parameters (Å, deg) for clusters **4** and **5**.

	B97-3c	BP86-D3/def2-TZVP(-f)	X-ray
$[TiO(O^iPr)(Cy_2PO_2)]_4$ (**4**)			
Ti1–O1 (oxo)	1.892	1.904	1.937(2)
Ti1–O1′ (oxo)	2.156	2.157	2.121(2)
Ti1–O1′′ (oxo)	1.933	1.946	1.912(2)
Ti1–O2 (isopropoxide)	1.796	1.804	1.790(2)
Ti1–O3 (eq. phosphinate)	2.030	2.025	2.011(3)
Ti1–O4 (ax. phosphinate)	2.018	2.014	2.021(3)
P1–O3	1.546	1.544	1.522(3)
P2–O4	1.546	1.544	1.518(3)
Ti1···Ti1′	2.862	2.876	2.911(12)
Ti1–O1–Ti1′	96.84	96.64	98.25(10)–99.16(10)
O3–P1–O3′	115.85	116.49	114.2(2)
O4–P2–O4′	115.87	116.75	114.9(2)
$[TiO(O^iPr)(Ph_2PO_2)]_4$ (**5**)			
Ti–O (short)	1.893–1.929	1.899–1.954	1.908(19)–1.945(18)
Ti–O (long)	2.169–2.173	2.156–2.179	2.121(2)–2.159(2)
Ti–OPOPh$_2$	2.013–2.048	2.008–2.040	2.014(2)–2.057(2)
Ti–O^{i}Pr	1.778–1.790	1.790–1.801	1.773(2)–1.794(2)
P–O	1.540–1.545	1.539–1.545	1.522(2)–1.529(2)
Ti1···Ti3	2.843	2.855	2.914(7)
Ti2···Ti4	2.840	2.855	2.912(7)
O–P–O	115.44–118.05	115.59–119.05	114.69(11)–115.19(11)
O1–Ti1–O3	82.91	82.10	80.59(8)
O1–Ti1–O4	79.92	79.57	79.86(8)
O1–Ti1–O5	100.19	94.16	97.63(9)
O1–Ti1–O9	163.90	164.50	164.54(8)
O1–Ti1–O15	93.092	92.25	94.25(8)

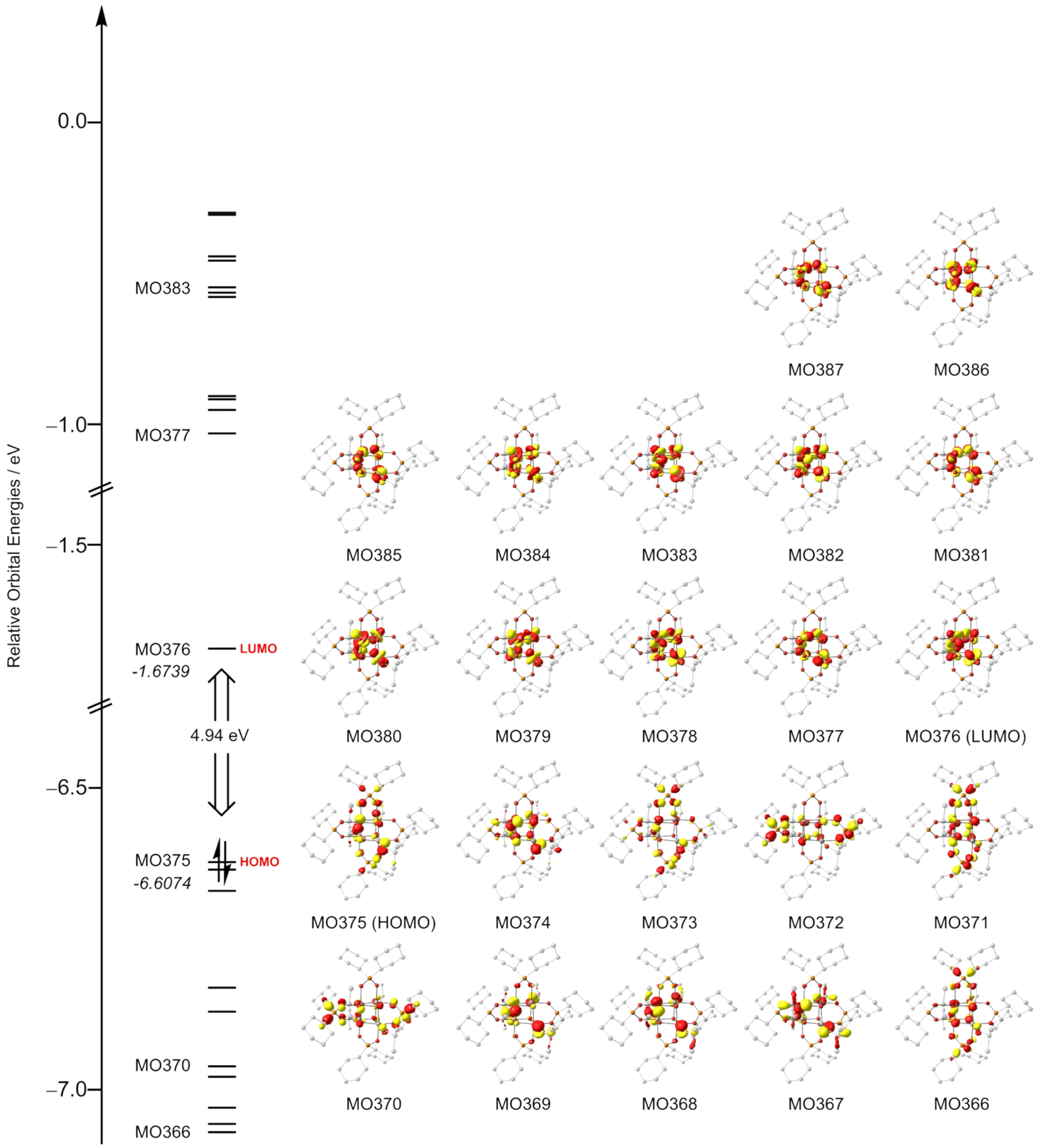

Figure S40. Canonical Kohn-Sham molecular orbital diagram (B3LYP/def2-TZVP(–f)/def2-SV(P)) of **4**.

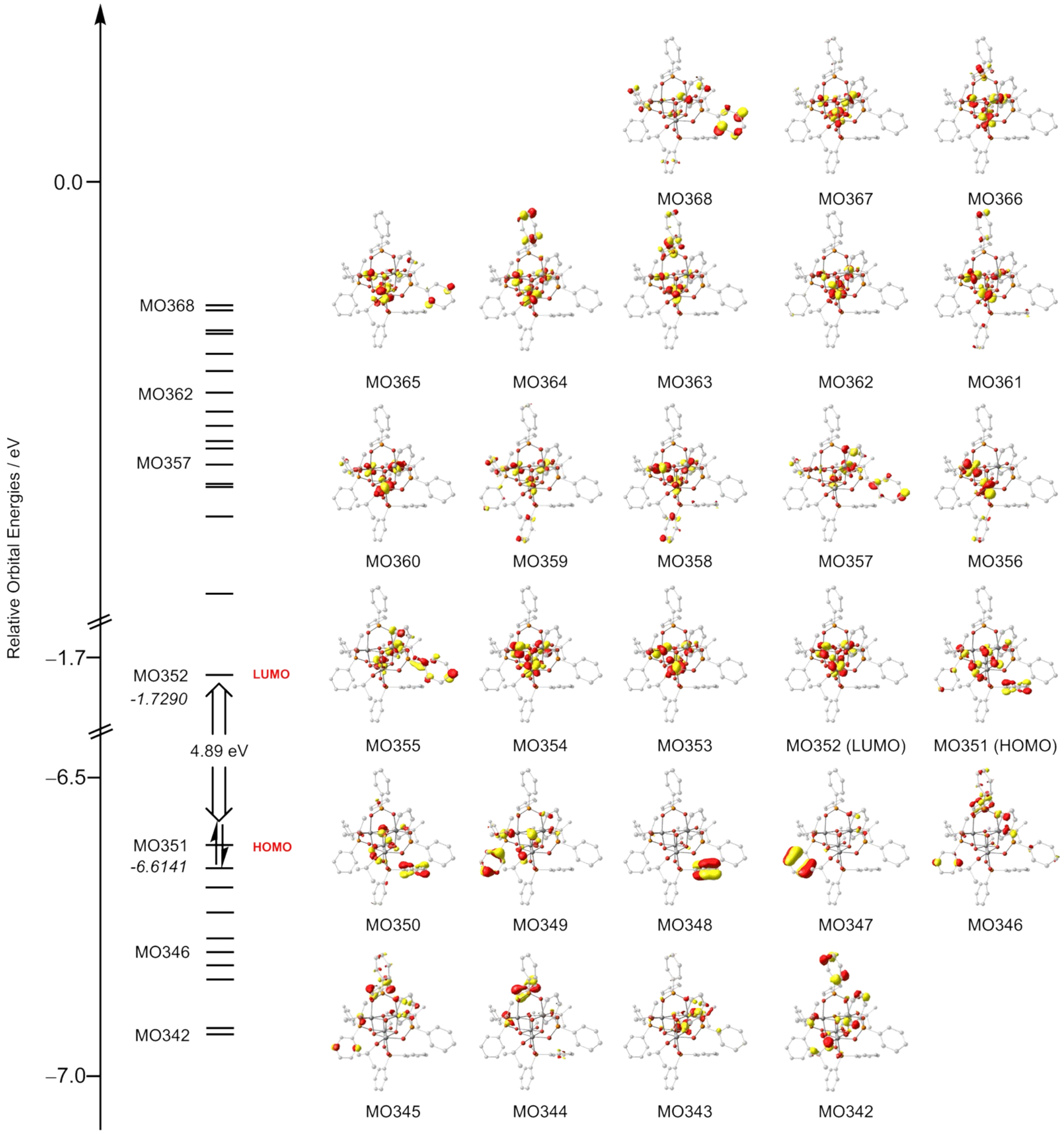

Figure S41. Canonical Kohn-Sham molecular orbital diagram (B3LYP/def2-TZVP(–f)/def2-SV(P)) of **5**.

Table S2. Contributions from Ti and O centres to key canonical Kohn-Sham orbitals in the frontier orbital region of **4** and **5**.

$[TiO(O^iPr)(Cy_2PO_2)]_4$ (**4**)	Oxo (O)	Alkoxide (O)	Phosphinate (O)	Ti centres
HOMO–4 (MO371)	30.1%	9.0%	20.0%	2.6%
HOMO–3 (MO372)	18.6%	2.8%	25.6%	1.2%
HOMO–2 (MO373)	3.6%	22.9%	25.6%	1.5%
HOMO–1 (MO374)	22.4%	44.1%	5.8%	2.0%
HOMO (MO375)	10.8%	36.9%	15.7%	3.1%
LUMO (MO376)	4.4%	2.0%	4.9%	85.3%
LUMO+1 (MO377)	3.6%	9.9%	6.8%	78.4%
LUMO+2 (MO378)	10.6%	0.4%	2.8%	81.5%
LUMO+3 (MO379)	1.2%	3.8%	4.2%	87.1%
LUMO+4 (MO380)	0.8%	4.4%	1.4%	87.8%
$[TiO(O^iPr)(Ph_2PO_2)]_4$ (**5**)				
HOMO–4 (MO347)	3.6%	3.0%	1.7%	0.3%
HOMO–3 (MO348)	2.3%	1.8%	1.3%	–
HOMO–2 (MO349)	13.2%	15.8%	4.8%	0.8%
HOMO–1 (MO350)	15.0%	15.1%	4.9%	0.7%
HOMO (MO351)	23.6%	17.6%	2.0%	1.3%
LUMO (MO352)	4.8%	2.5%	4.4%	82.2%
LUMO+1 (MO353)	3.6%	6.8%	2.7%	75.6%
LUMO+2 (MO354)	8.9%	1.2%	2.8%	79.8%
LUMO+3 (MO355)	0.7%	1.5%	2.9%	41.5%
LUMO+4 (MO356)	1.0%	2.8%	1.7%	67.9%

Table S3. Dependence of TDDFT first excitation energies (in eV) for **4** and **5** on level of theory.

	**4**	**5**
B3LYP	3.97	3.93
PBE0	4.19	4.14
CAM-B3LYP	4.49	4.48
ωB97xD	4.56	4.55
M06	4.08	4.02
PBE	3.13	3.12

Photoirradiation Experiments

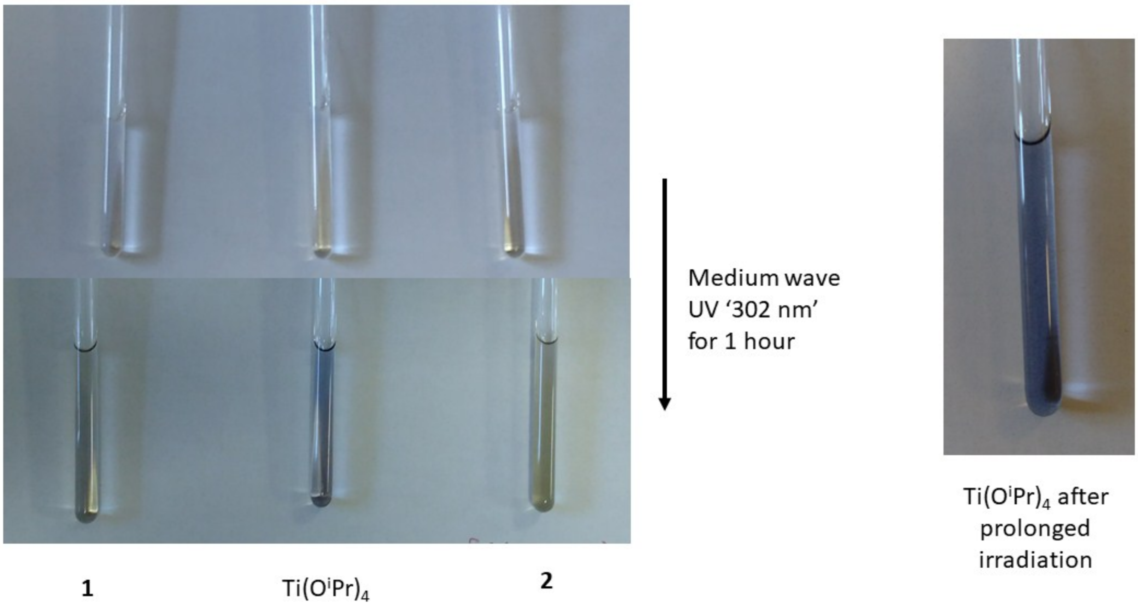

Figure S42. Photographs of d^8-toluene solutions of **1**, **2** and Ti(O^{i}Pr)$_4$ all with excess ^{i}PrOH before and after irradiation with medium wave UV light for 1 hour.

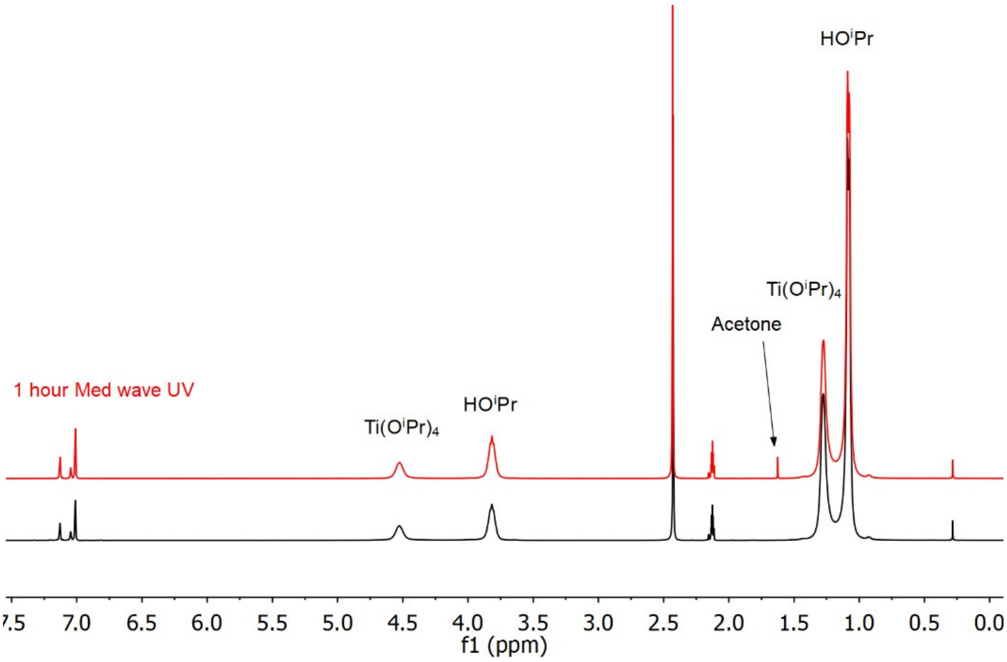

Figure S43. ^{1}H NMR spectra of Ti(O^{i}Pr)$_4$ + excess ^{i}PrOH in d^8-toluene before and after 1 hour irradiation with medium wave UV light

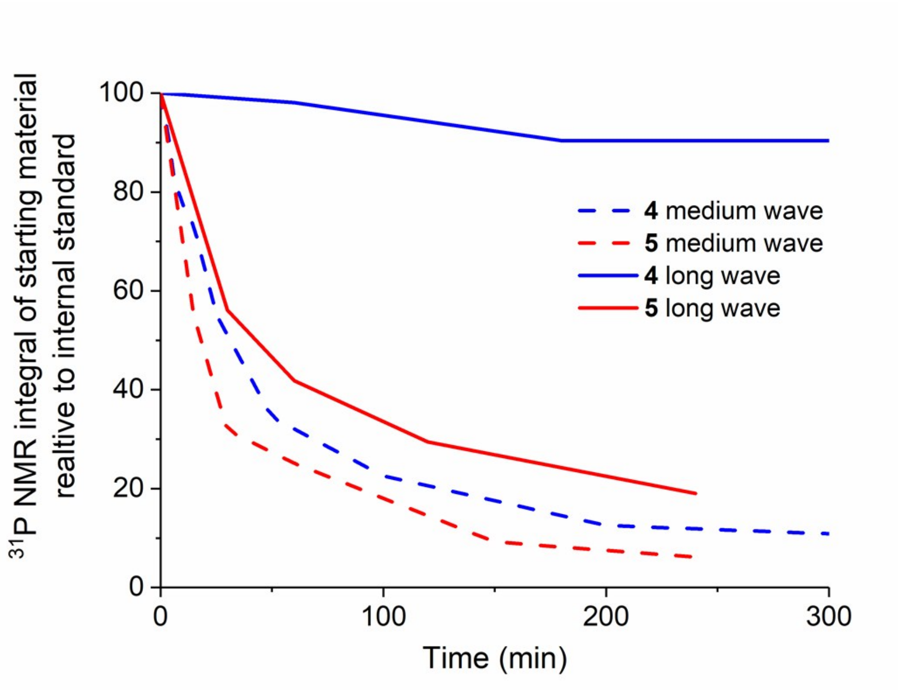

Figure S44. Consumption of starting material (**4** or **5**) during photoreduction of a solution of **4** or **5** with 30 equiv. HO^{i}Pr in d^8-toluene with either long or medium wave UV irradiation over time. Measured by integral of the ^{31}P NMR signal relative to internal standard (PPh_3 capilliary).

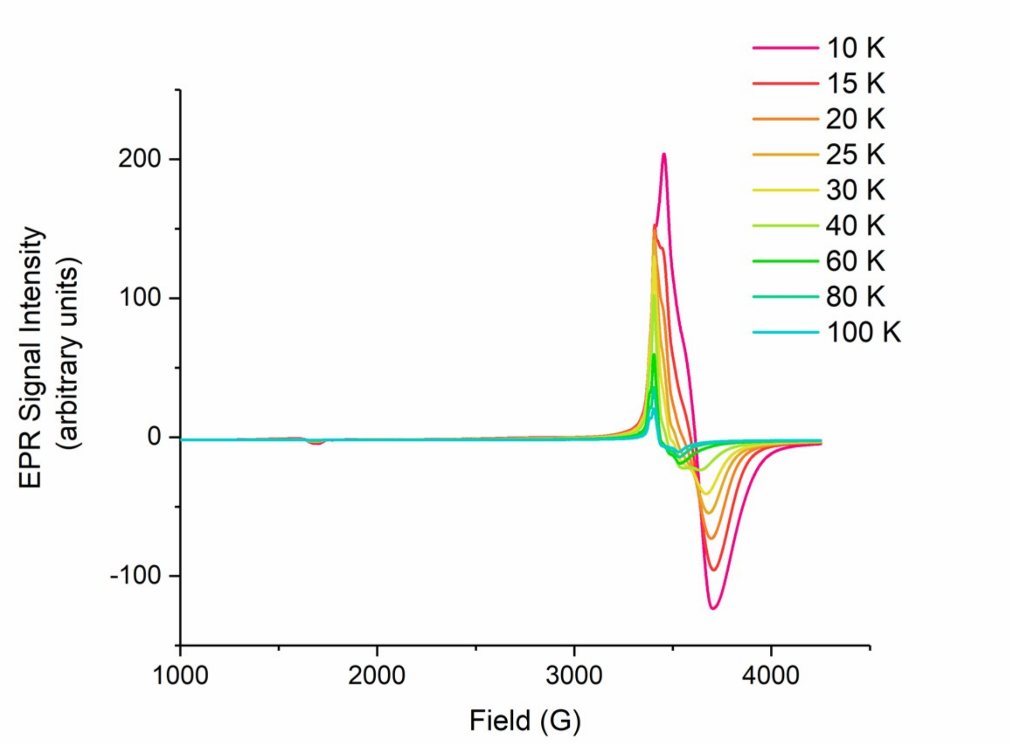

Figure S45. Variable temperature EPR spectra of **4** after photoreduction with MW-UV (7.5 h, [**4**] = 10 mM, 9:1 toluene:^{i}PrOH). N.B. It was not possible to accurately determine the concentration of paramagnetic centres using EPR spectroscopy due to the complexity of the system (containing S = 1 molecules), however, the intensity of the EPR signals are far too strong to be associated with a trace impurity signal and the double integral (DI) value for the major component of the EPR spectra is proportional with the molar concentration of the solutions measured.

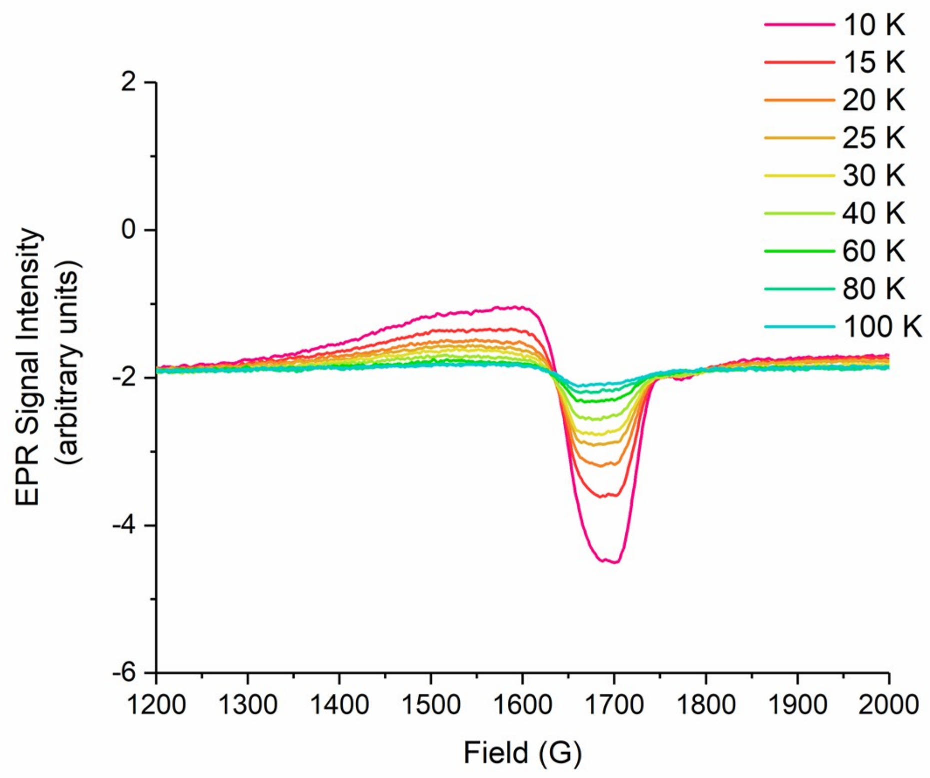

Figure S46. Variable temperature EPR spectra of **4** after photoreduction with MW-UV (7.5 h, [**4**] = 10 mM, 9:1 toluene:^{i}PrOH) showing signal at 1650 G.

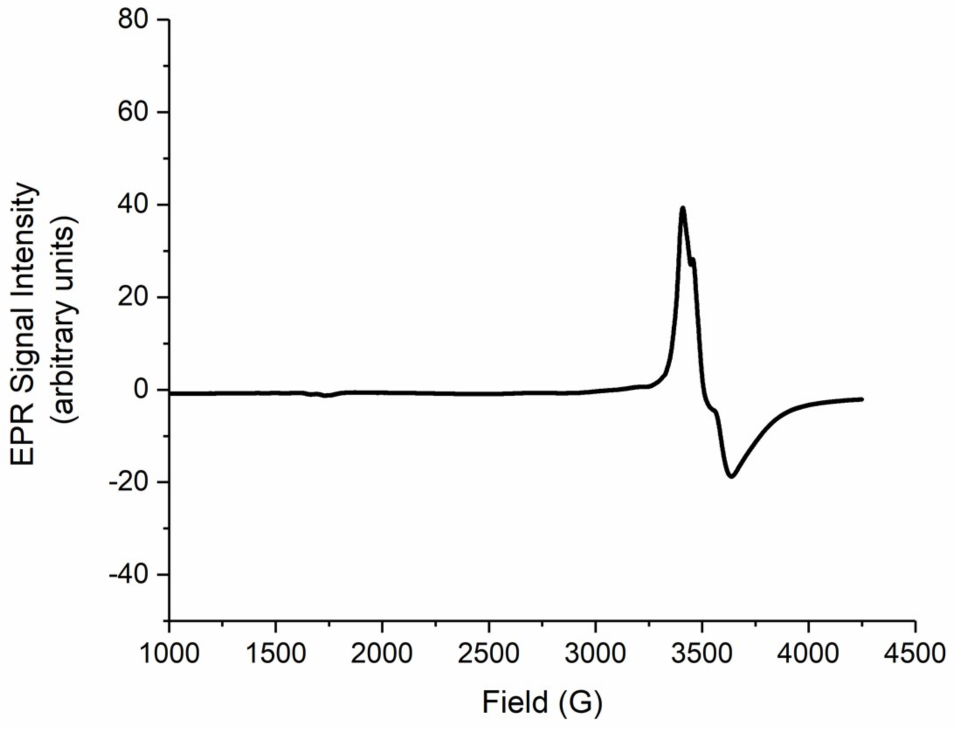

Figure S47. EPR spectra (10 K) of **5** after photoreduction with MW-UV (3 h, [**5**] = 4 mM, 9:1 toluene:^{i}PrOH).

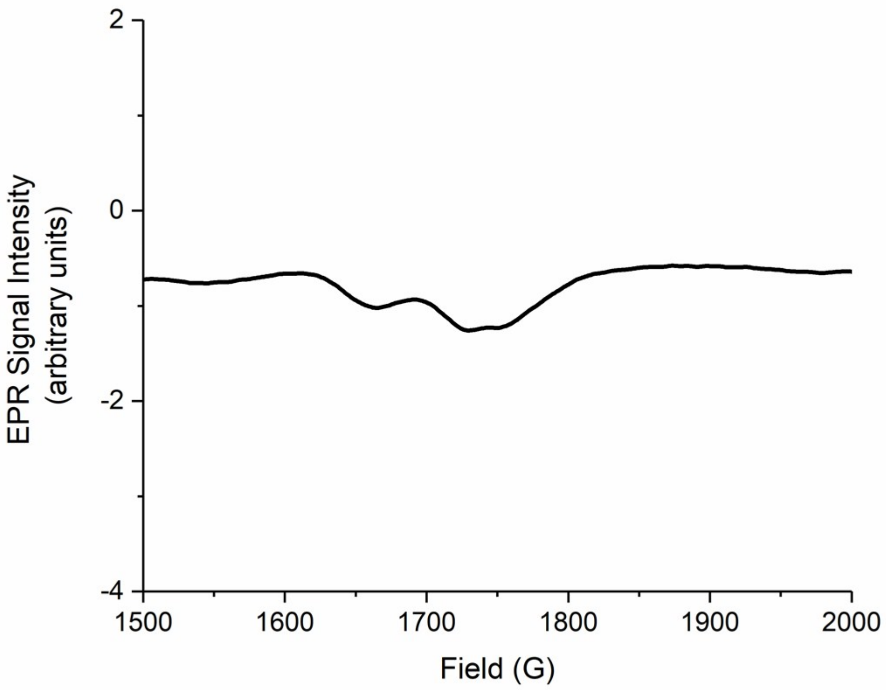

Figure S48. EPR spectra (10 K) of **5** after photoreduction with MW-UV (3 h, [**5**] = 4 mM, 9:1 toluene:^{i}PrOH) showing weak signal at ~1700 G.

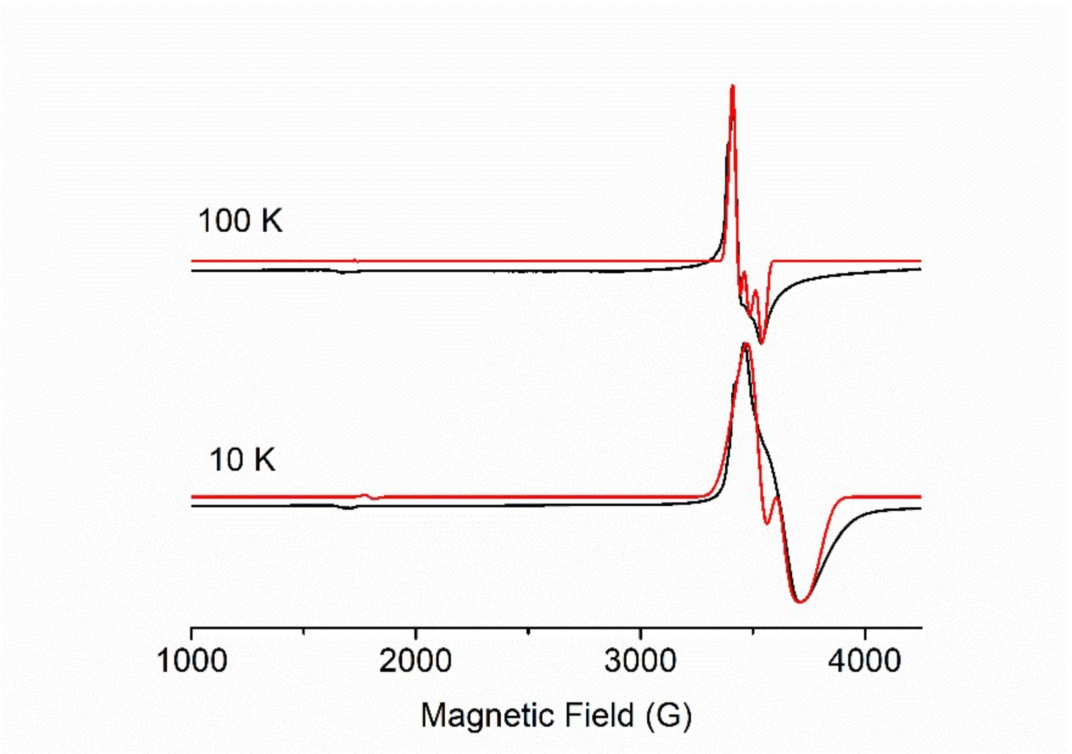

Figure S49. Experimental (black) and simulated (red) X-band EPR spectrum at 100 K and 10 K of photoreduced **4**. At 100 K modelled with S = 1; g_{xy} = 1.926, g_z=1.938; D = 0.0075 cm^{-1} and E = 0.0013 cm^{-1}. At 10 K modelled with modelled with S = 1; g_{xy} = 1.860, g_z=1.868; D = 0.0165 cm^{-1} and E = 0.0023 cm^{-1}

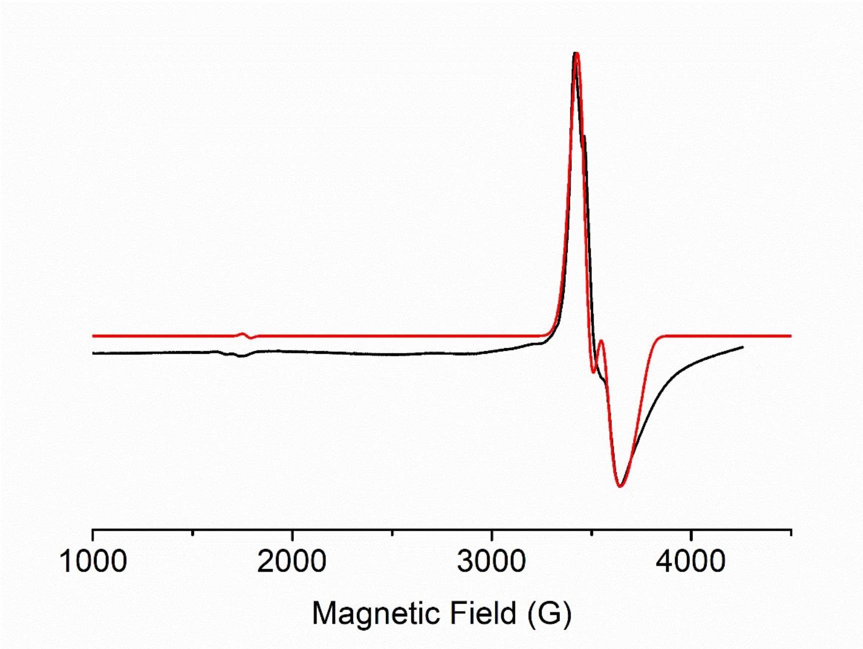

Figure S50. Experimental (black) and simulated (red) X-band EPR spectra of photoreduced **5**, at 10 K modelled with S = 1; g_{xy}= 1.880; g_z = 1.905; D = 0.015 cm^{-1} and E = 0.0012 cm^{-1}

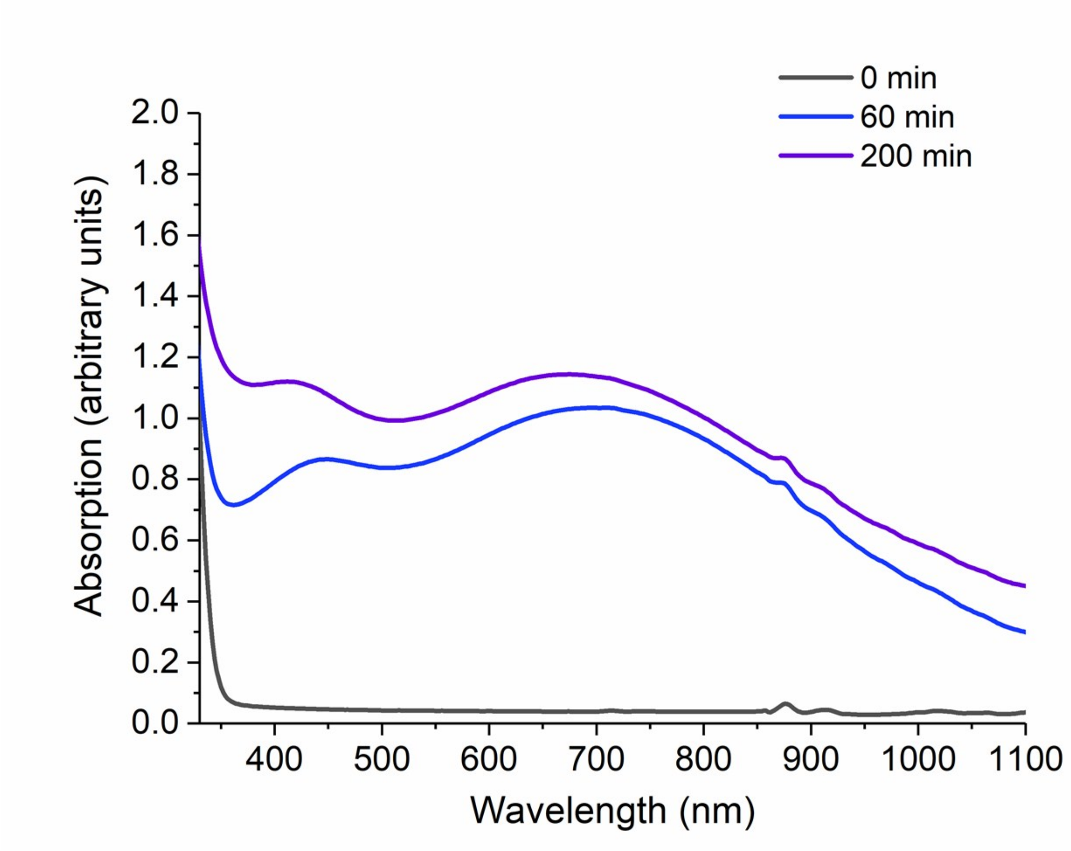

Figure S51. UV/visible spectra of a toluene solution of **5** ([Ti] = 7.3 mM) before and after irradiation with long wave UV.

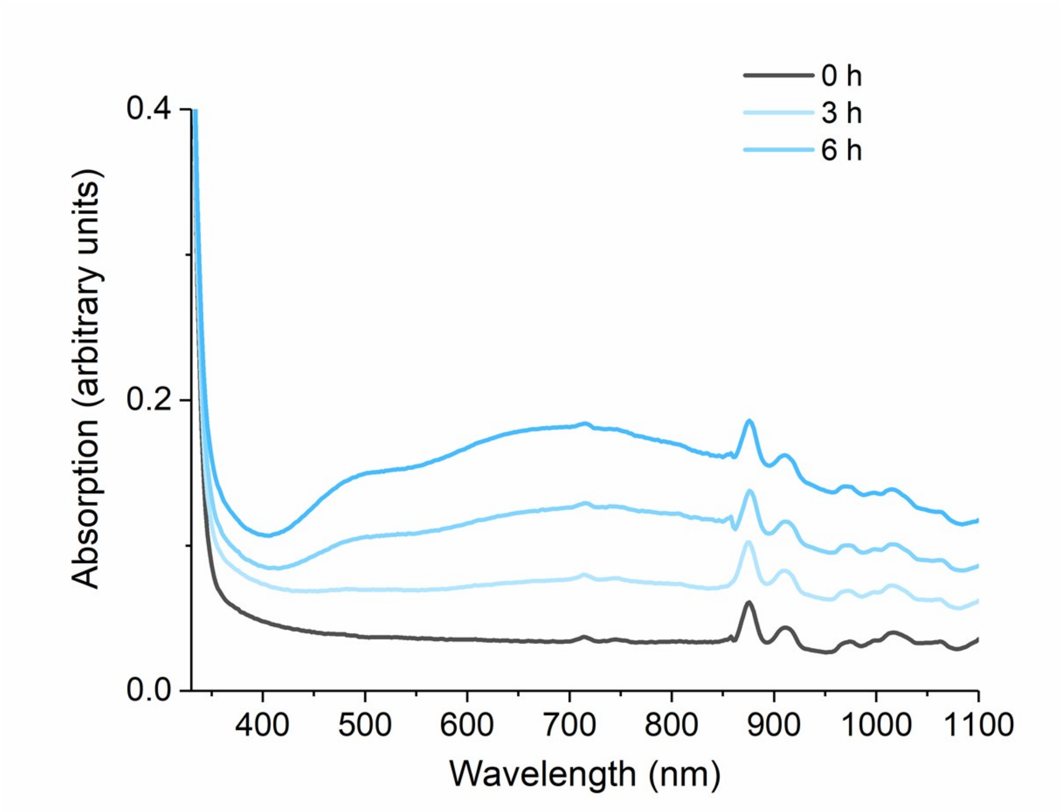

Figure S52. UV/visible spectra of a toluene solution of **4** ([Ti] = 7.3 mM) before and after irradiation with long wave UV.

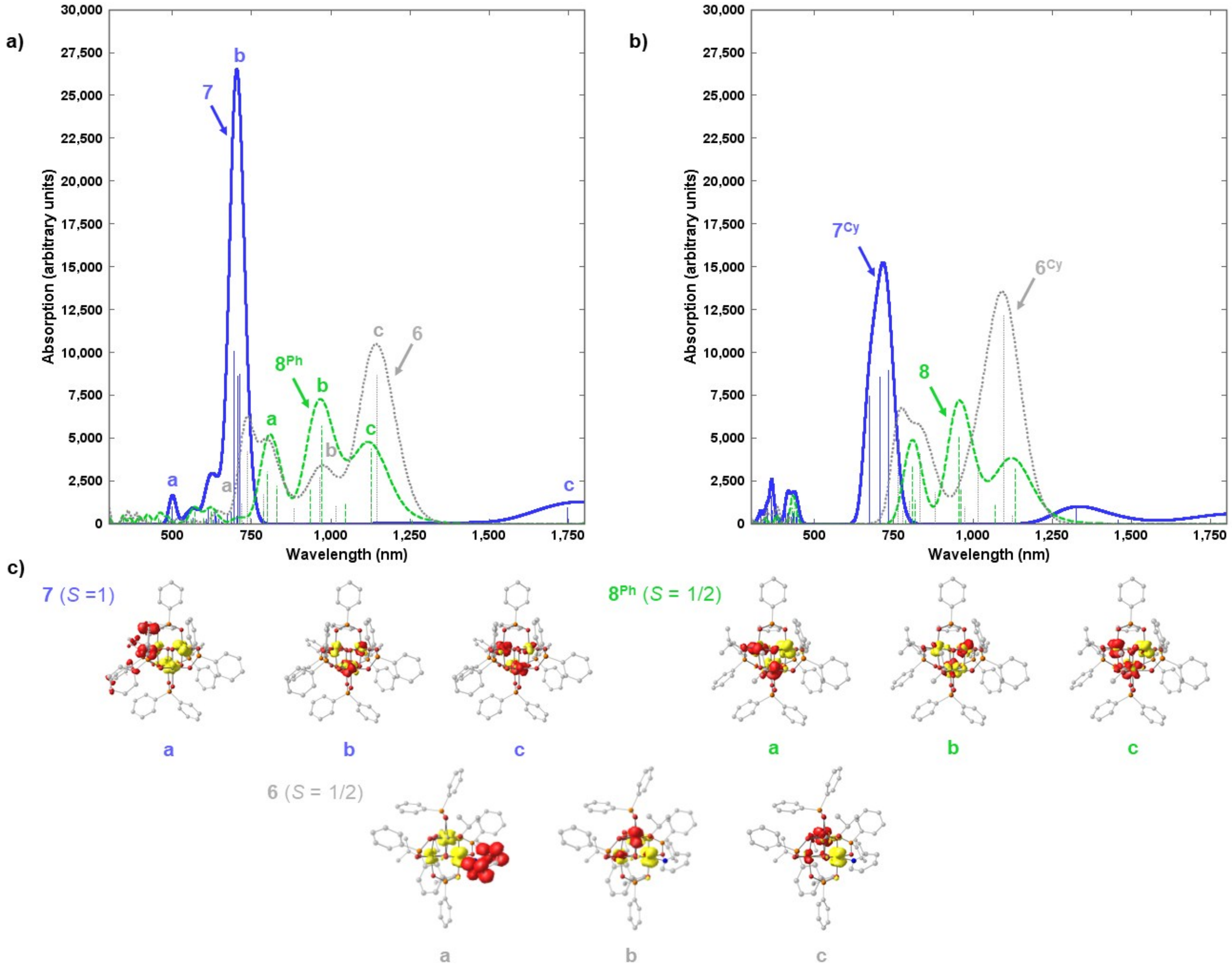

Figure S53. Calculated TDDFT absorption spectra in toluene solvent for reduced cluster based on the a) diphenylphosphinate $[O_2PPh_2]^-$ and b) dicyclophosphinate $[O_2PCy_2]^-$ ligands. c) Isosurfaces of the electron difference densities (contoured at 0.003 au) between selected excited and ground states for clusters **6**, **7** and **8^{Ph}** as representative examples. Yellow regions correspond to the depletion of the electron density during the transition while red regions correspond to the accumulation of electrons. ZORA-B3LYP/def2-TZVP(-f)/def2-SV(P). **7**b, **7**c, **8**a, **8**b, **8**c and **6**b and **6**c correspond to cluster d-d transitions and/or intervalence charge transfer, **7**a and **6**a refer to metal to ligand charge transfer e.g. 3d to π*, where the π* is located on Ph_2PO_2 ligand in **7** and on the pyridine ligand in **6**.

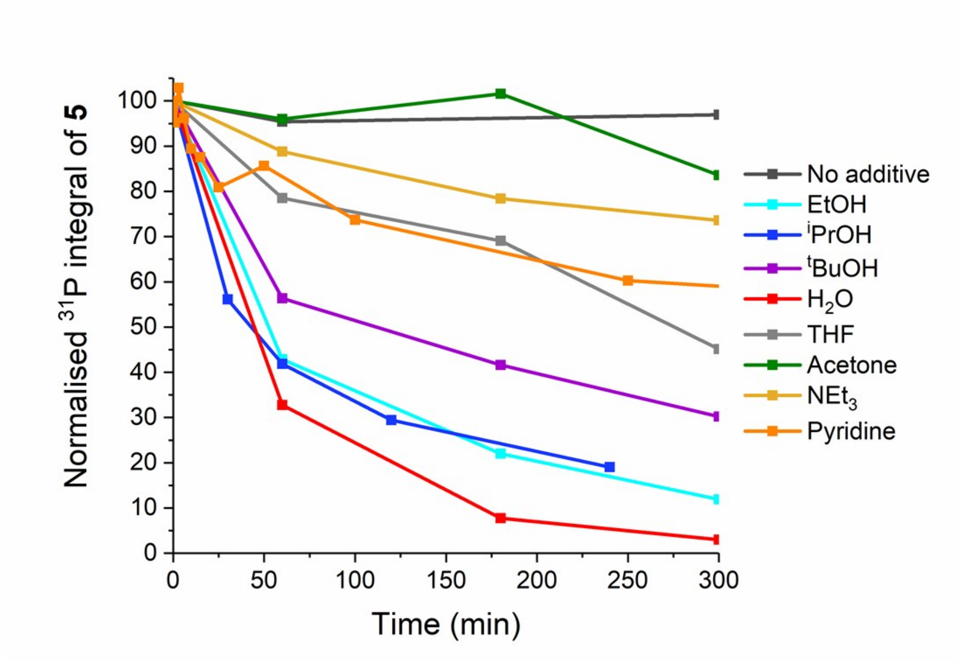

Figure S54. Consumption of **5** during irradiation with LW-UV over time. Integrals taken from ^{31}P NMR spectrum and normalised to 100 at start of experiment. Partial alkoxide exchange expected with EtOH (see supporting note 2), ^{t}BuOH or H_2O during photoreduction. When H_2O is added, replacement of O^{i}Pr with OH may lead to further condensation and a dark blue precipitate is formed during this reaction, 97% of **5** is consumed after 5 hours irradiation either as a paramagnetic complex and/or precipitate, an acetone signal indicative of 23% conversion of Ti−O^{i}Pr to acetone is also observed in the ^{1}H NMR spectrum.

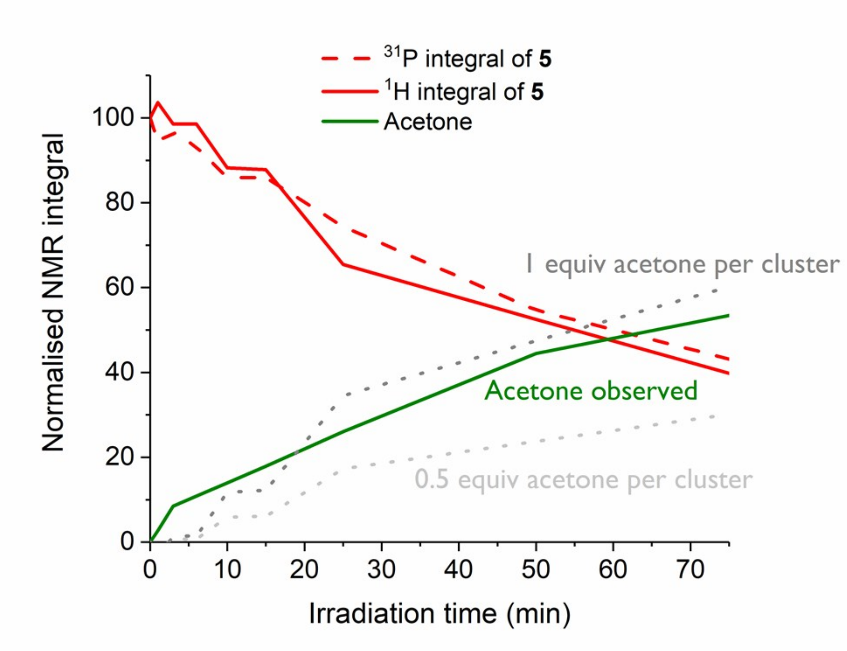

Figure S55. Normalised integrals from ^{1}H and ^{31}P NMR spectroscopy showing consumption of **5** during LW-UV irradiation in the presence of ^{i}PrOH and production of acetone. Dotted lines show the expected amount of acetone that will be produced by a 1-electron redox process (0.5 equiv per cluster) or a 2-electron redox process (1 equiv per cluster) estimated from the consumption of ^{1}H integrals of **5**. N.B. ^{i}PrOH is also anticipated to form in the same quantity as acetone.

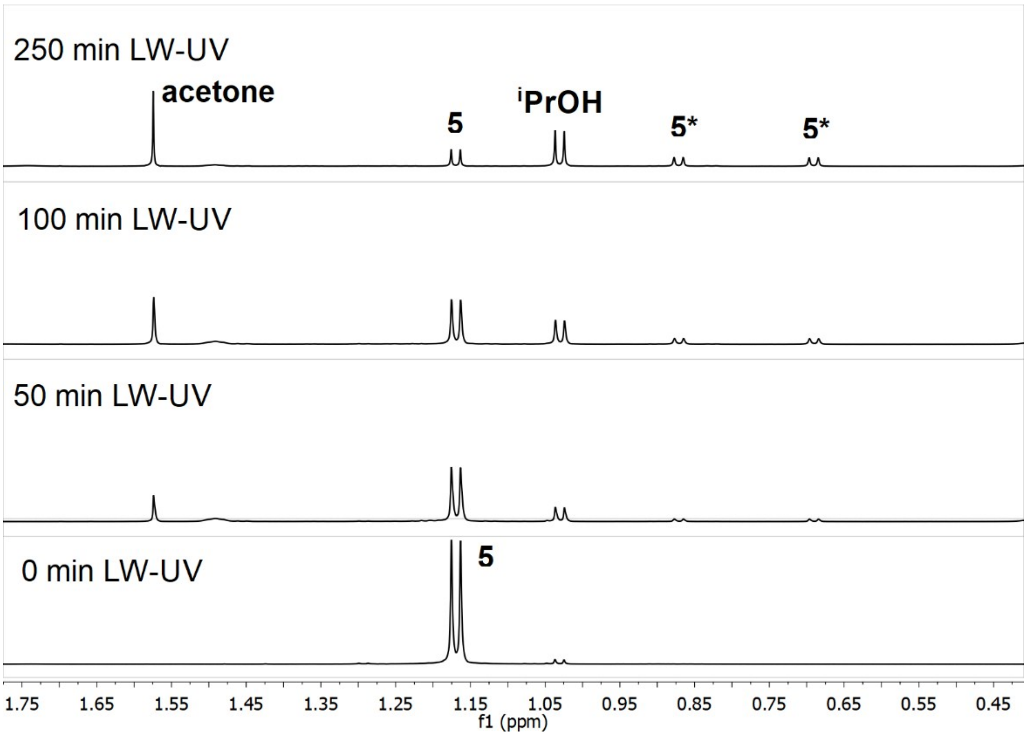

Figure S56. ^{1}H NMR spectra showing the consumption of **5** under LW-UV irradiation in the presence of pyridine. Acetone and ^{i}PrOH grow in at the same rate and a trace of unidentified **5*** is also formed.

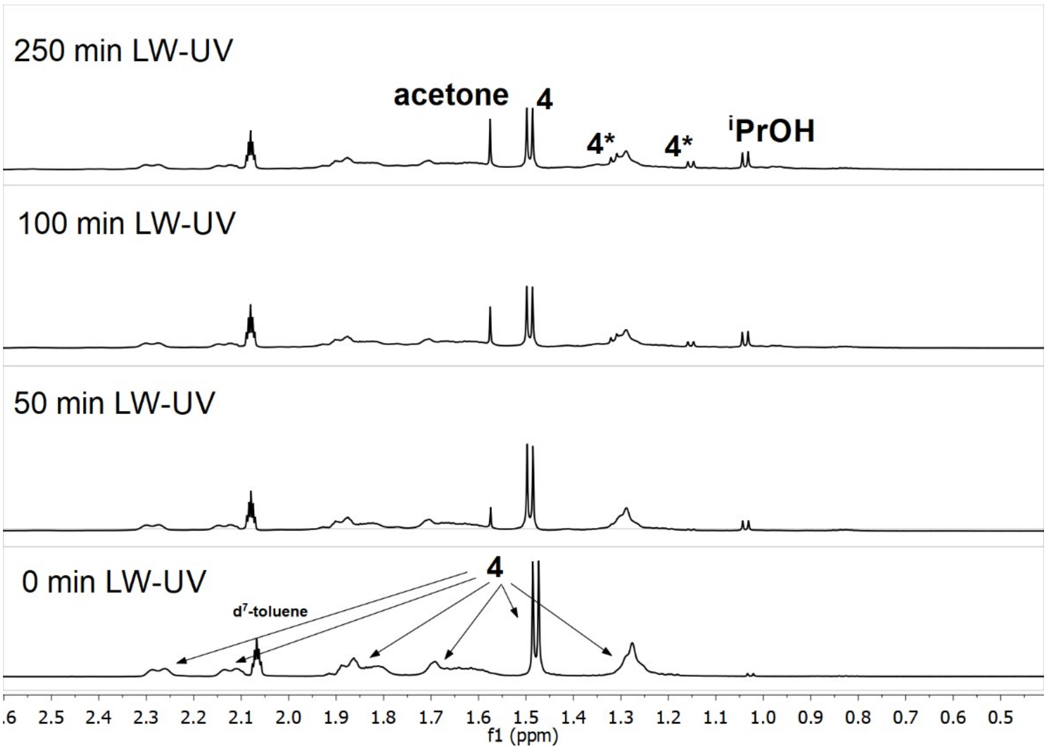

Figure S57. ^{1}H NMR spectra showing the consumption of **4** under MW-UV irradiation in the presence of pyridine. Acetone and ^{i}PrOH grow in at the same rate and a trace of unidentified **4*** is also formed.

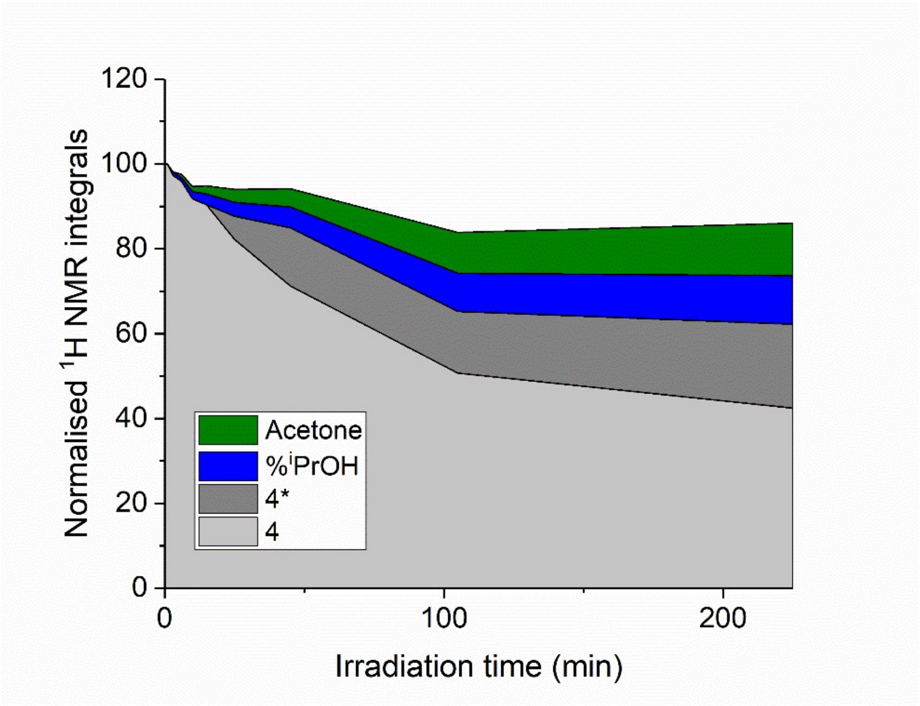

Figure S58. Area graph showing ^{1}H NMR spectroscopy Integrals (Me groups of ^{i}Pr signals) of starting material **4** during MW-UV irradiation in the presence of pyridine, unknown complex **4*** and organic products acetone and ^{i}PrOH. The loss of signal (sum below 100%) consistent with O^{i}Pr groups coordinated to paramagnetic complexes which were not located by ^{1}H NMR spectroscopy.

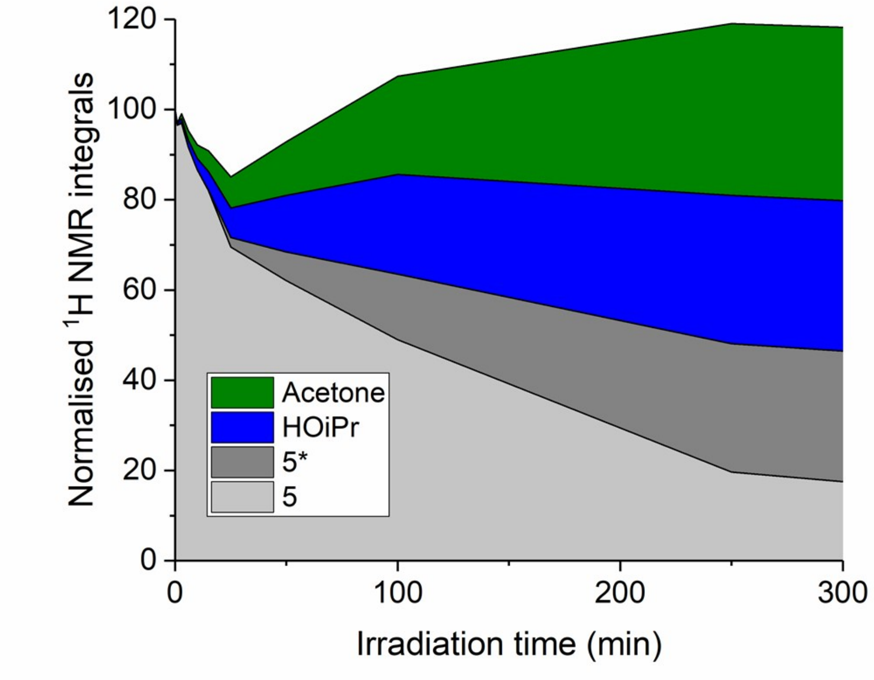

Figure S59. Area graph showing ^{1}H NMR spectroscopy Integrals (Me groups of ^{i}Pr signals) of starting material **5**, unknown complex **5*** and organic products acetone and ^{i}PrOH during LW-UV irradiation in the presence of pyridine. Loss of signal over initial period consistent with O^{i}Pr groups coordinated to paramagnetic complexes which were not located by ^{1}H NMR spectroscopy. Later growth of signal (sum ~100%) suggests further preferential reaction of the paramagnetic species with prolonged irradiation.

X-ray crystallography and DFT calculations for 6

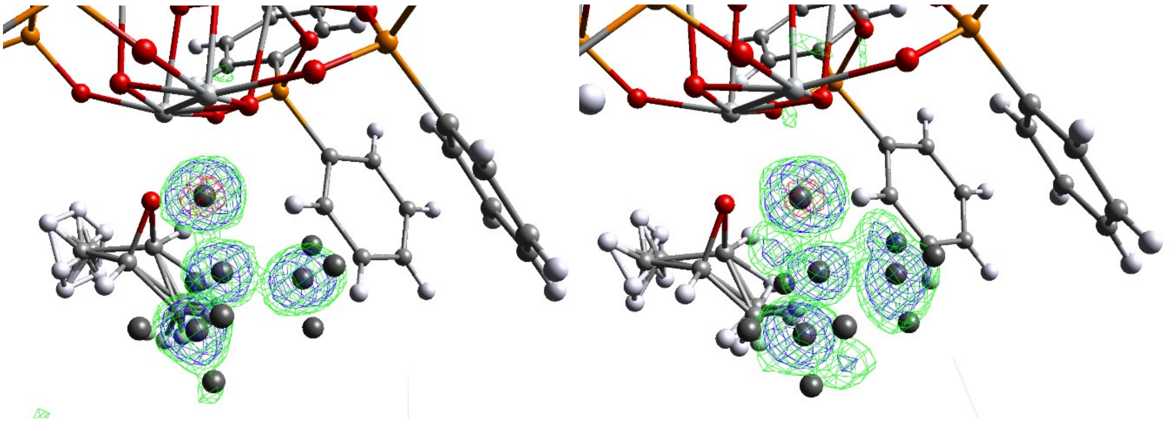

Figure S60. Electron density map generated using MCE[31] of O^iPr group connected to Ti(1) (O(5)-C(49)-C(50)-C(51)) in solid-state structure of **5** (left) and again in data from deep blue crystals containing co-crystallisation of **5** (72%) and **6** (28%), black spheres represent location of O, C and H atoms in refined model (pyridine locations in **6** removed for clarity). Extra electron density freely refines to a hexagonal shaped disorder component, consistent with a pyridine occupying the coordination site. In the reported structure of **6** (Figure 6e), symmetry restraints are added to help model the pyridine component. No extra electron density was located at the other three O^iPr sites, however two of these sites are disordered over two O^iPr positions and so could mask any further minor co-crystallised species.

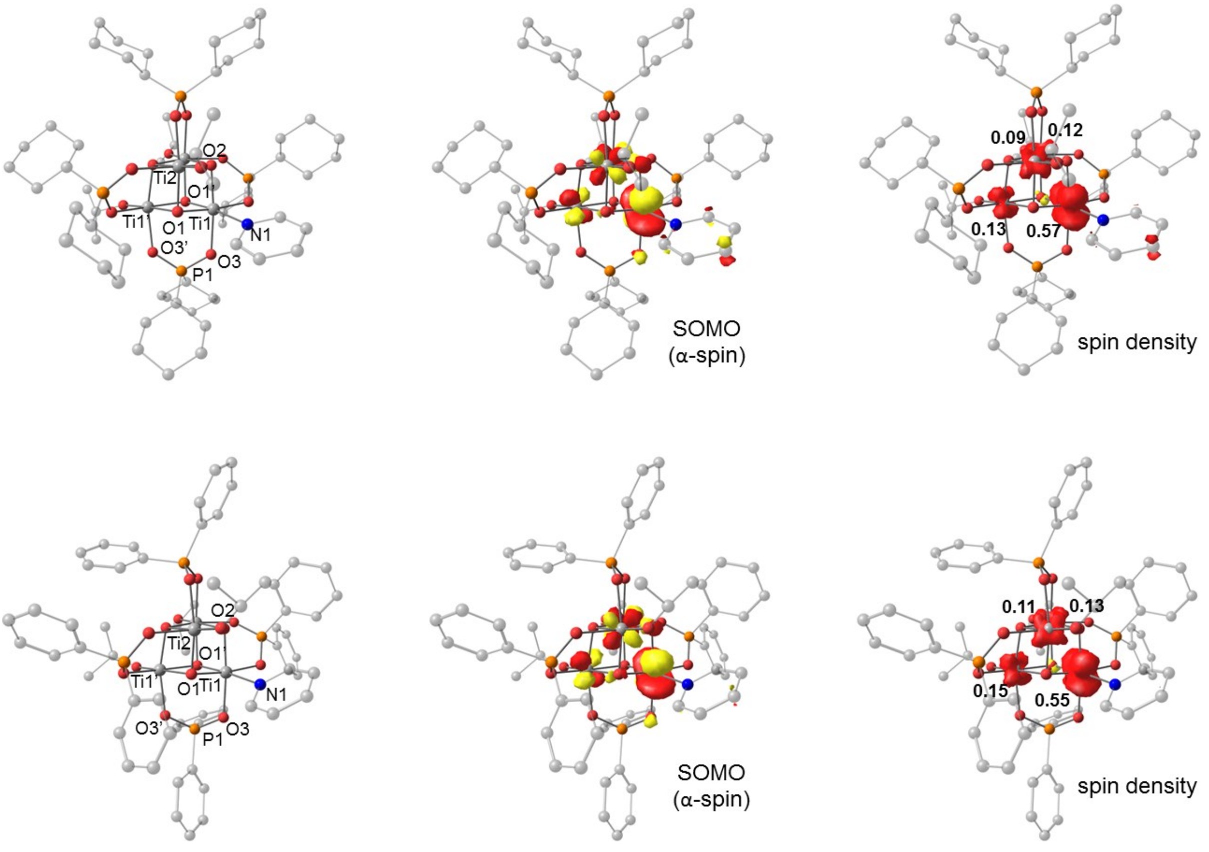

Figure S61. DFT-optimized geometries (B97-3c) of clusters $\mathbf{6}^{Cy}$ (left, R = Cy) and **6** (right, R = Ph) in their doublet (S = 1/2) ground states. Hydrogen atoms omitted for clarity. Singly-occupied α-spin molecular orbitals are shown along with spin density plots and Loewdin atomic spin populations.

Table S4. Key DFT-optimized bond parameters (Å, deg) for clusters **6**^{Cy} (R = Cy) and **6** (R = Ph).

	**6**^{Cy} (R = Cy)	**6** (R = Ph)
	S = 1/2	*S* = 1/2
Ti1–O1 (oxo)	1.885	1.893
Ti1–O1′ (oxo)	1.945	1.927
Ti1′–O1 (oxo)	2.141	2.175
Ti1′–O1′ (oxo)	1.929	1.940
Ti2–O2 (iso-propylate)	1.814	1.799
Ti2–O1 (oxo)	1.941	1.918
Ti1–N1	2.214	2.211
Ti–$OPOPh_2$	2.016–2.078	2.042–2.089
P–O	1.543–1.556	1.534–1.553
Ti1···Ti1′	2.969	2.981
Ti2···T2′	3.063	3.089
Ti1···T2	2.833	2.812
Ti1···T2′	3.125	3.153
Ti1–O1–Ti1′	94.79	94.00
O–P–O	116.15–116.72	115.84–118.78

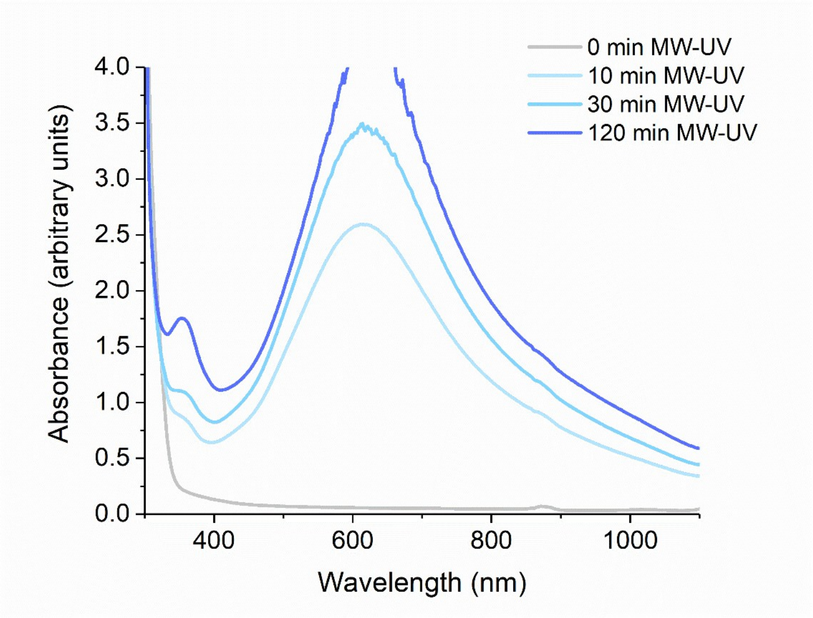

Figure S62. UV/vis. spectra of a neat pyridine solution of **4** under MW-UV irradiation, [**4**] = 1.95 x10^{-3}.

Characterisation of 5^{Bux} compounds

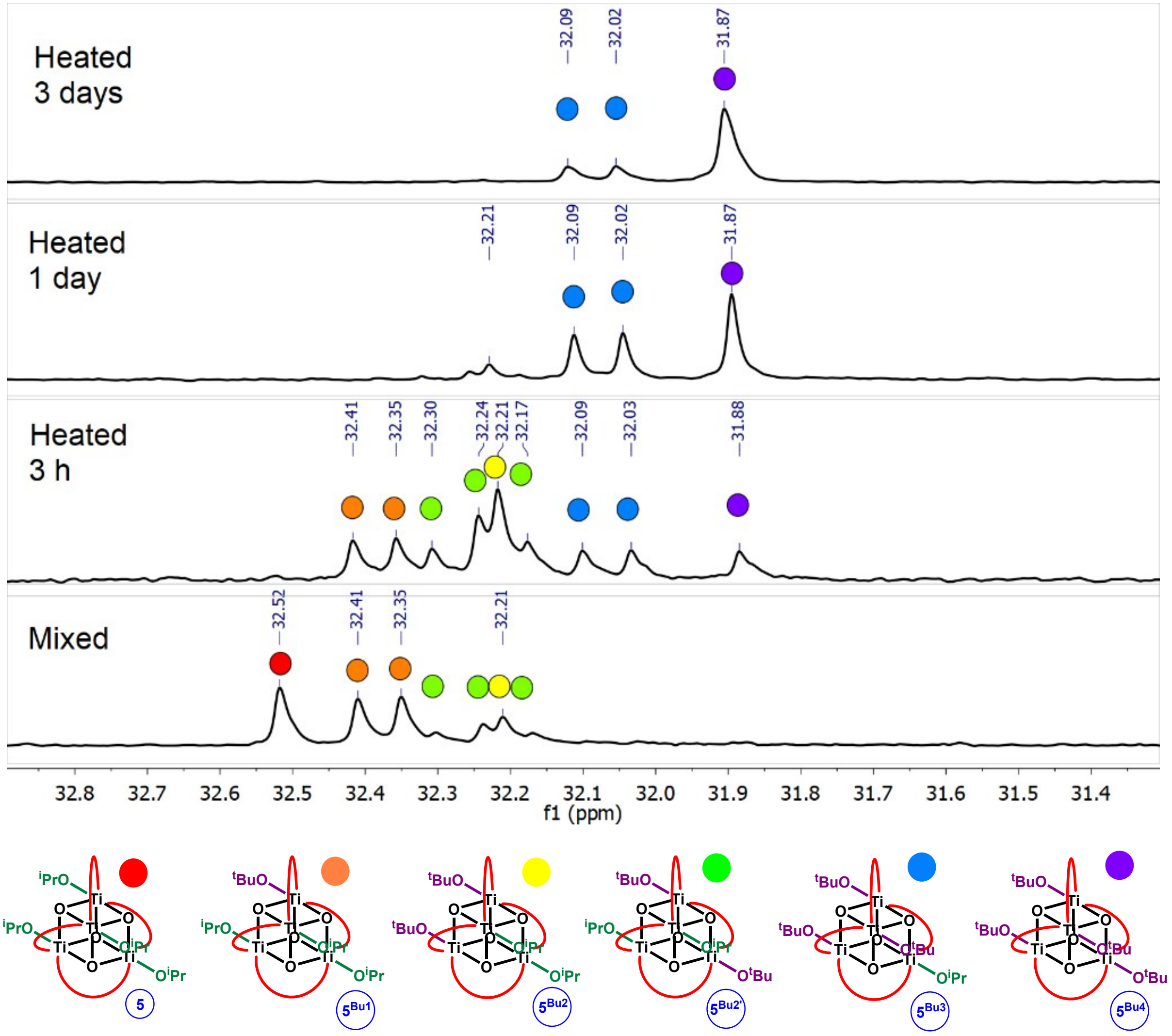

Figure S63. $^{31}P\ \{^{1}H\}$ NMR spectra of **5** + 300 equiv. ^{t}BuOH in toluene after heating to 70°C for various time periods. Compounds **5** (red, single signal), $\mathbf{5^{Bu1}}$ (orange, two signals, 1:1 ratio), $\mathbf{5^{Bu2}}$ (yellow, single signal), $\mathbf{5^{Bu2'}}$ (green, three signals, 1:2:1 ratio), $\mathbf{5^{Bu3}}$ (blue, two signals, 1:1 ratio) and $\mathbf{5^{Bu4}}$ (purple, single signal) are all identified and consistent with the expected P environments in the alkoxide exchange products (shown below, red curves represent $[O_2PPh_2]^-$).

5$^{\mathbf{Bu4}}$. [TiO(O^{t}Bu)(Ph_2PO_2)]$_4$.

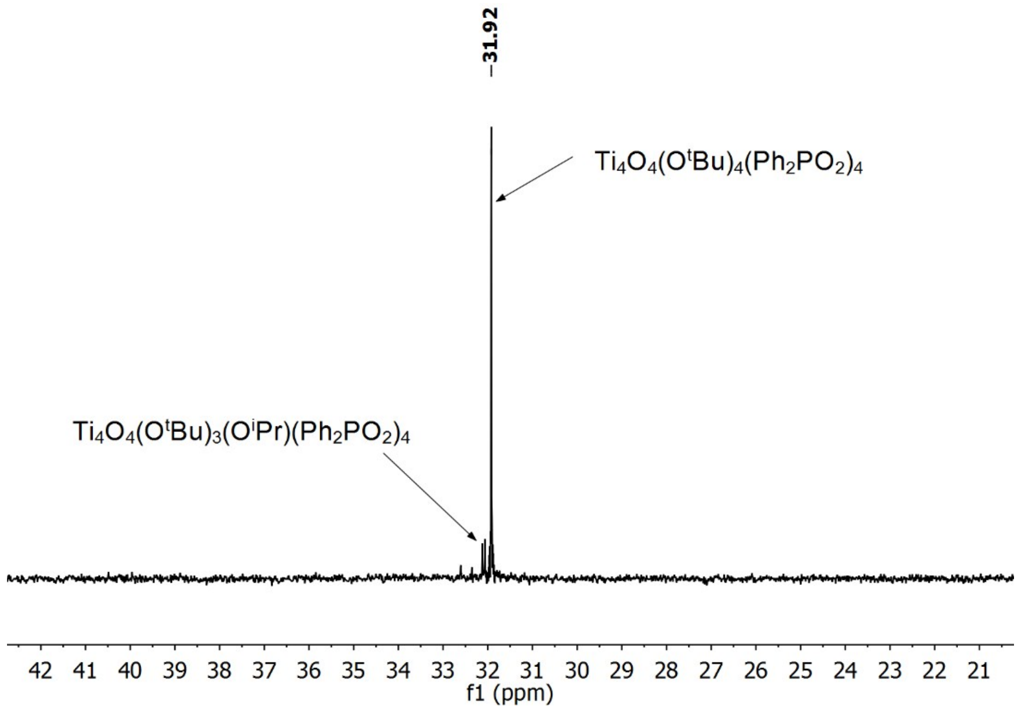

Figure S64. $^{31}P\{^1H\}$ NMR spectrum of **5**$^{\mathbf{Bu4}}$ (d^8-toluene, 162 MHz). N.B. a trace of **5**$^{\mathbf{Bu3}}$ is observed as a two minor signals.

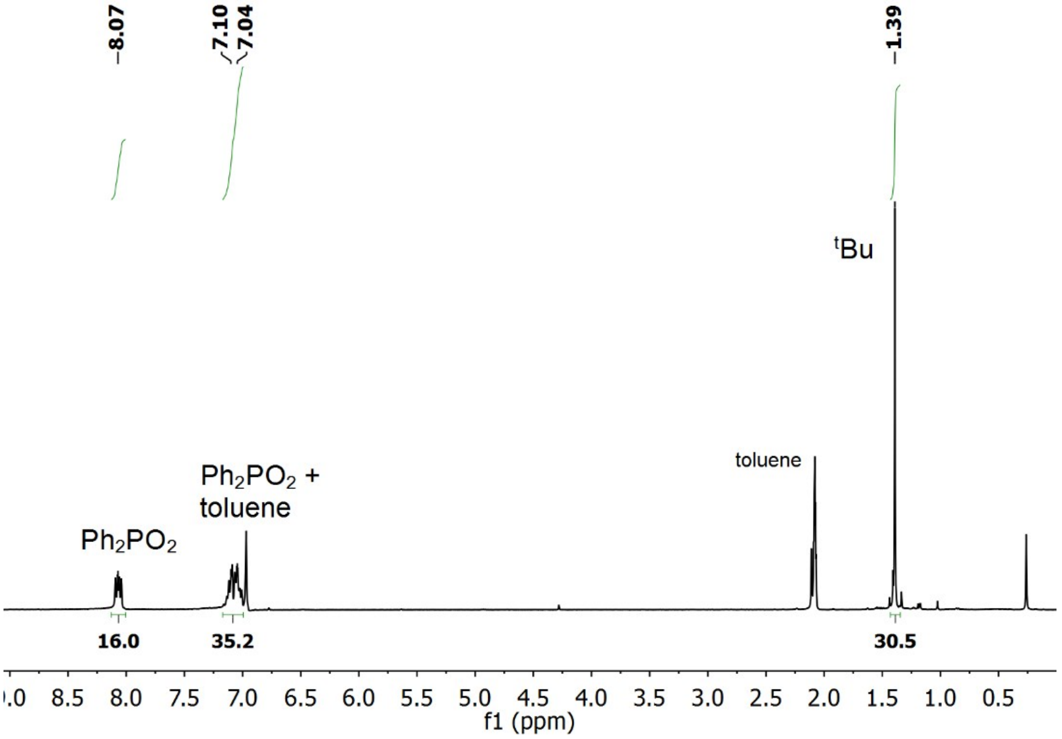

Figure S65. ^1H NMR spectrum of **5**$^{\mathbf{Bu4}}$ (d^8-toluene, 400 MHz). N.B. a trace of **5**$^{\mathbf{Bu3}}$ results in extra very minor O^{t}Bu and O^{i}Pr signals.

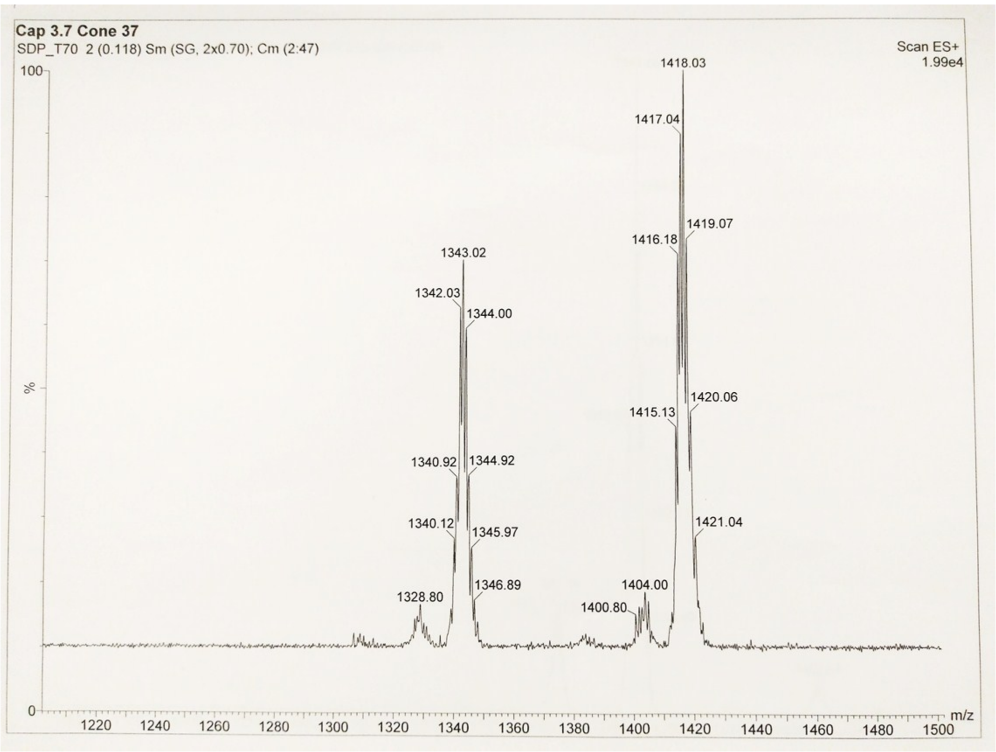

Figure S66. ESI Mass Spectrum (CH_2Cl_2) of **5^{Bu4}** (with trace of **5^{Bu3}**): {[TiO(O^{t}Bu)(Ph_2PO_2)]$_4$H}$^+$ found 1417.0, predicted 1417.2, [Ti_4O_4(O^{t}Bu)$_3$(Ph_2PO_2)$_4$]$^+$ found 1343.0, predicted 1343.1. Also minor signals for {[Ti_4O_4(O^{t}Bu)$_3$(O^{i}Pr)(Ph_2PO_2)$_4$]H}$^+$ and [Ti_4O_4(O^{t}Bu)$_2$(O^{i}Pr)$_1$(Ph_2PO_2)$_4$]$^+$ at 1404.0 and 1328.8 respectively (from **5^{Bu3}**)

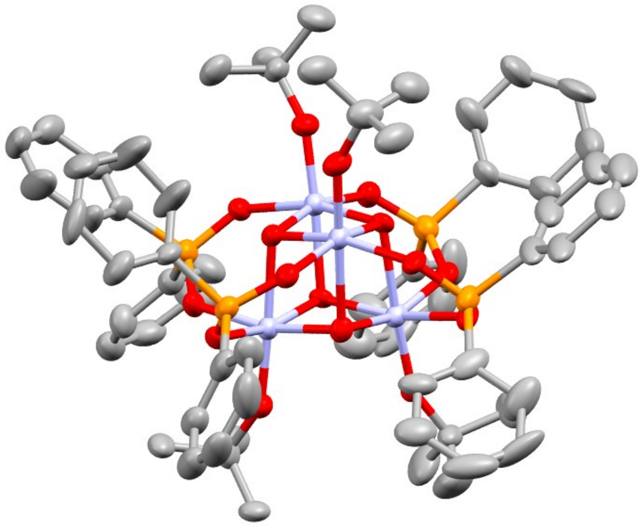

Figure S67. Solid-state structure of **5^{Bu4}**. Crystallographic analysis of **5^{Bu4}** proved difficult and data was collected at a synchrotron facility. This dataset was solved to confirm the expected connectivity of the complex. Two molecules were located in the asymmetric unit, so that 191 heavy atoms were present. Disorder was found in several ^{t}Bu, Ph and toluene groups which were modelled over two sites and restrained where possible and if sensible solutions obtained. A twin law of -1, 0, 0, 0.564, 0. 0.13, 0, 0, -1 was found to improve the refinement with the twin having a volume fraction of 28%. Due to the difficulties, we suggest that the structure confirms the expected connectivity, but should not be used for accurate bond lengths and angles.

Photoirradiation of 5^{Bu3}

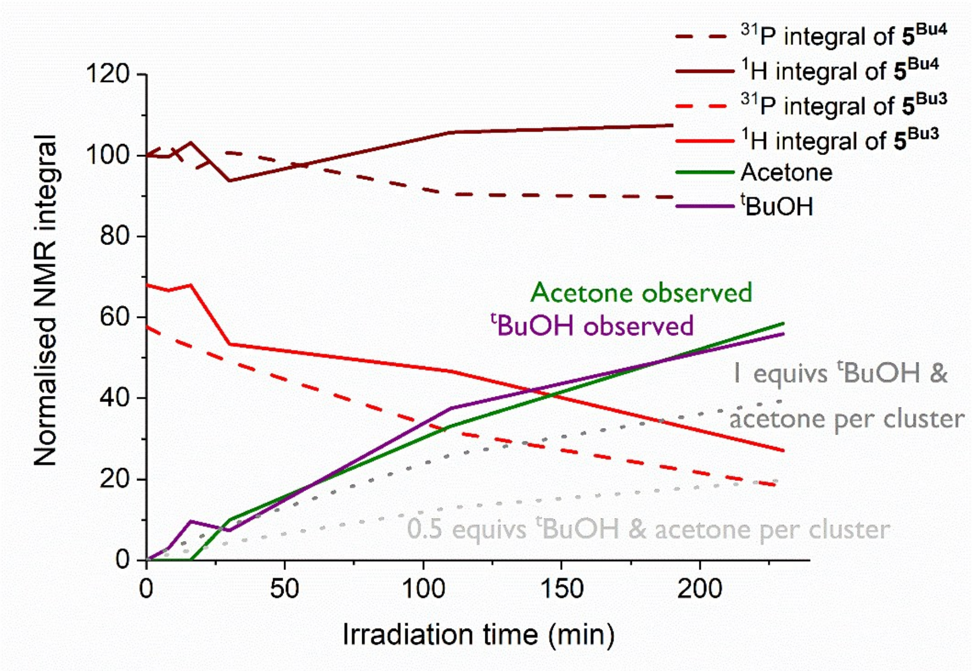

Figure S68. Normalised integrals from ^{1}H and ^{31}P NMR spectroscopy showing consumption of $\mathbf{5^{Bu3}}$ during LW-UV irradiation in the presence of pyridine and production of ^{t}BuOH and acetone. Dotted lines show the expected amount of acetone that will be produced by a 1-electron redox process (0.5 equiv per cluster), or a 2-electron redox process (1 equiv per cluster) estimated from the consumption of ^{1}H integrals of $\mathbf{5^{Bu3}}$. N.B. $\mathbf{5^{Bu4}}$ acts as a spectator and is not consumed during the reaction. This reaction was repeated with a mixture of $\mathbf{4^{Bu3}}$ and $\mathbf{4^{Bu4}}$ with MW-UV irradiation, similarly acetone and ^{t}BuOH were produced as $\mathbf{4^{Bu3}}$ is consumed. In both cases no ^{i}PrOH was observed.

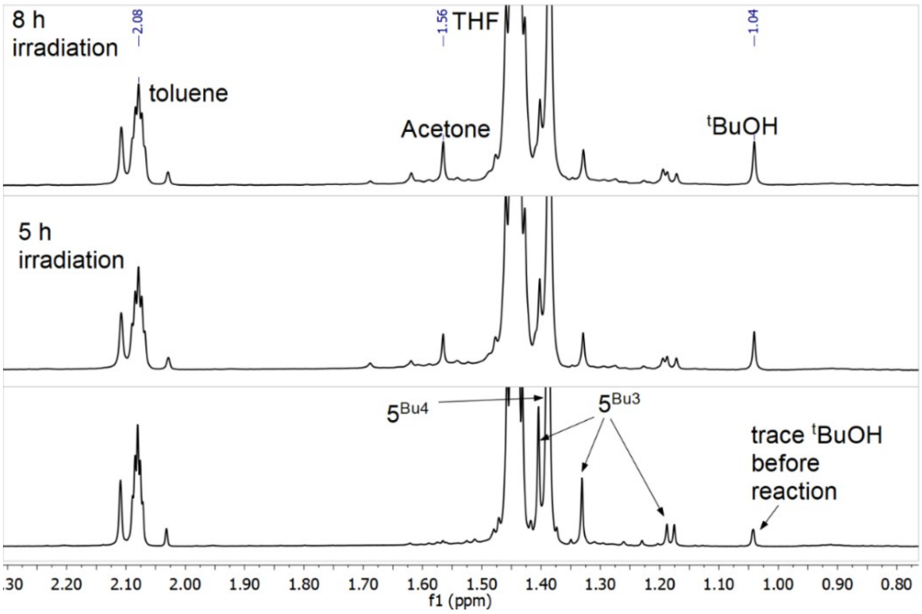

Figure S69. ^{1}H NMR spectrum of a d^{8}-toluene solution of $\mathbf{5^{Bu3}}$ (25%) and $\mathbf{5^{Bu4}}$ (75%) with 30 equiv. THF before and after irradiation with LW-UV. Signals for acetone and ^{t}BuOH appear but no ^{i}PrOH is observed (expected 1.03 ppm (d) and 3.77 ppm (septet)). The experiment was repeated with 30 equiv. pyridine (instead of THF) to yield similar results.

Bond-valence sum calculations

Table S5. Bond-valence sum calculations and indication of formal oxidation state of the Ti site.

Compound	Atom	Bond-valence sum (using parameters for Ti(iv), r_o = 1.815)[32]	Bond-valence sum (using parameters for Ti(iii) r_o = 1.791)[33]	Formal Oxidation State
**1**	Ti(1)	4.37	4.10	4
	Ti(2)	4.28	4.01	4
**3**	Ti(1)	4.54	4.25	4
	Ti(2)	4.33	4.06	4
	Ti(3)	4.45	4.17	4
**4**	Ti(1)	4.16	3.90	4
**5**	Ti(1)	4.09	3.83	4
	Ti(2)	4.06	3.81	4
	Ti(3)	4.04	3.78	4
	Ti(4)	4.10	3.84	4
**7**	Ti(1)	3.67	3.44	3.5
**7-ox**	Ti(1)	4.15	3.88	4
**8**	Ti(1)	4.13	3.87	4
	Ti(2)	3.62	3.40	3.5

Characterisation and DFT calculations for 7. $[Ti_4O_4(Ph_2PO_2)_6]$ and 7-ox $[Ti_4O_4(Ph_2PO_2)_6][O_2]_2$

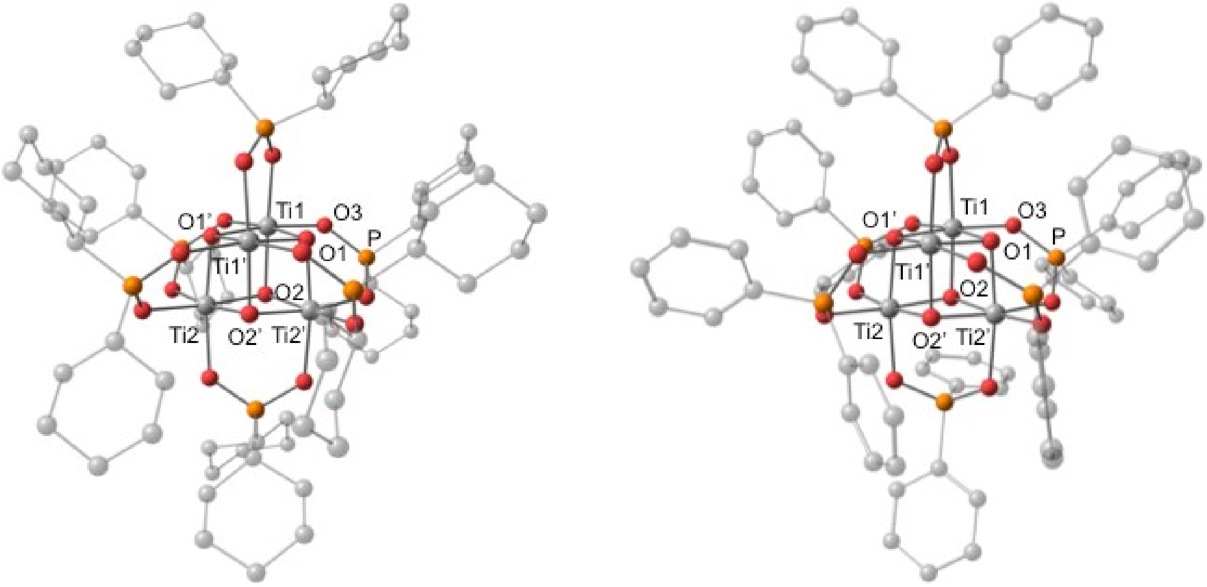

Figure S70. DFT-optimized geometries (B97-3c) of clusters 7^{Cy} (left, R = Cy) and **7** (right, R = Ph) in their triplet (*S* = 1) ground states. Hydrogen atoms omitted for clarity.

Table S6. Relative energies (in eV) of different spin states in cluster **7**.

	**B97-3c**	**B3LYP**
Triplet state (*S* = 1)	0.00	0.00
Closed-shell singlet state (*S* = 0)	0.21	0.26
Open-shell singlet state (M_s = 0)	0.15	0.06

Table S7. Key DFT-optimized bond parameters (Å, deg) for clusters $\mathbf{7}^{Cy}$ (R = Cy) and **7** (R = Ph).

	$\mathbf{7}^{Cy}$	**7**			
	S = 1	*S* = 1	*S* = 0	M_s = 0	X-ray
Ti1–O1 (oxo)	1.948	1.953	1.950	1.953	1.970(2)
Ti1–O1′ (oxo)	1.958	1.947	1.936	1.946	
Ti1–O2 (oxo)	1.949	1.947	1.959	1.947	
Ti2–O1′ (oxo)	1.946	1.950	1.944	1.950	
Ti2–O2 (oxo)	1.954	1.947	1.925	1.948	
Ti2–O2′ (oxo)	1.947	1.946	1.963	1.946	
Ti–OPOPh$_2$	2.030–2.048	2.030–2.048	2.008–2.060	2.030–2.047	2.025(3)
P–O	1.548–1.550	1.542–1.549	1.542–1.549	1.542–1.549	
Ti1···Ti1′	2.895	2.890	2.929	2.889	2.933(2)
Ti2···T2′	2.902	2.903	2.914	2.903	
Ti1···T2	2.916	2.899	2.926	2.899	
Ti1···T2′	2.889	2.894	2.819	2.894	
Ti1–O1–Ti1′	95.68	95.34	97.55	95.33	96.25(16)
Ti2–O2–Ti2′	96.04	96.46	97.63	96.46	
O–P–O	115.67–116.44	116.95–117.70	115.86–118.72	115.86–118.72	115.9(3)

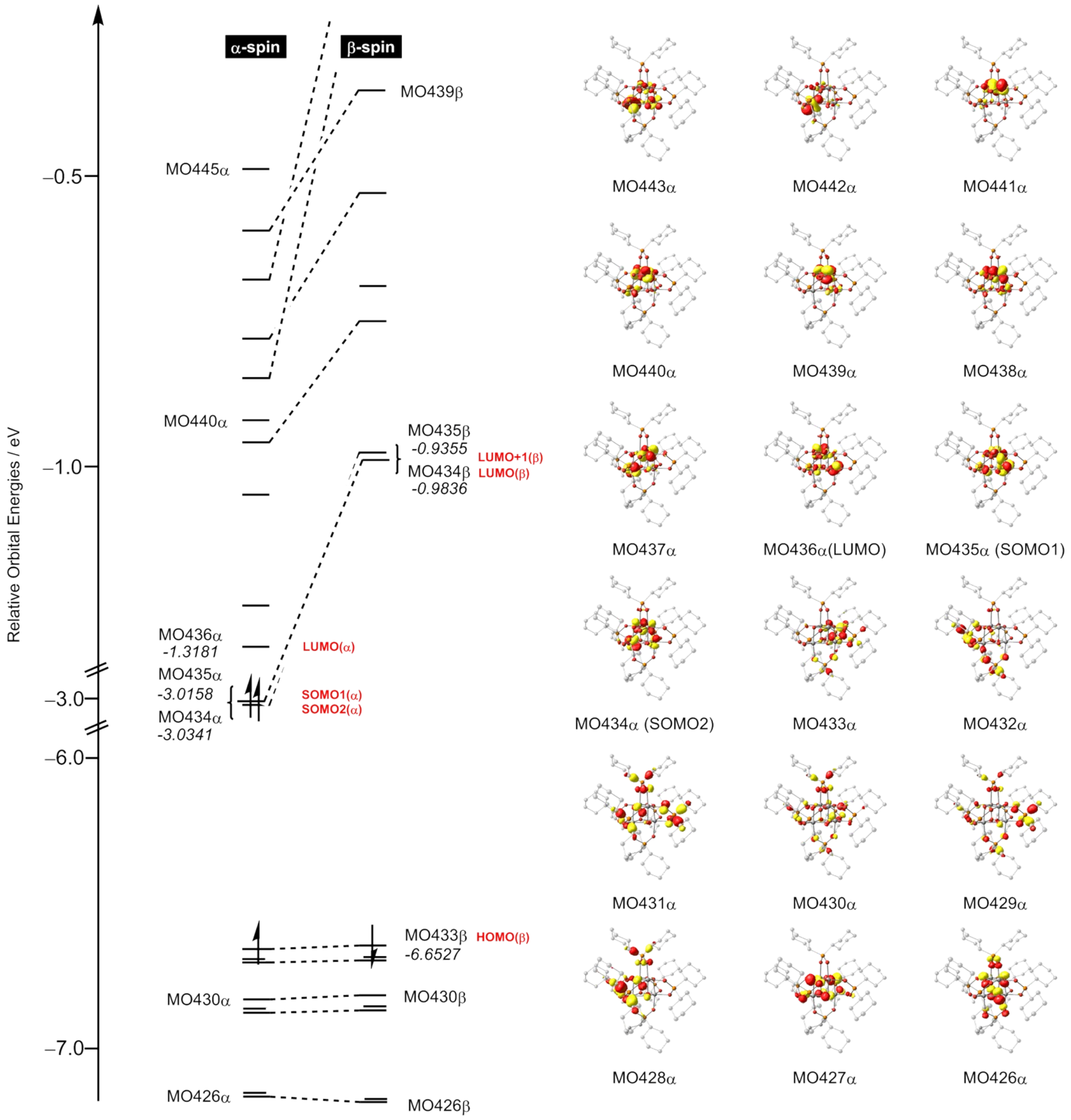

Figure S71. Canonical Kohn-Sham molecular orbital diagram (B3LYP/def2-TZVP(–f)/def2-SV(P)) of **7**^{Cy}.

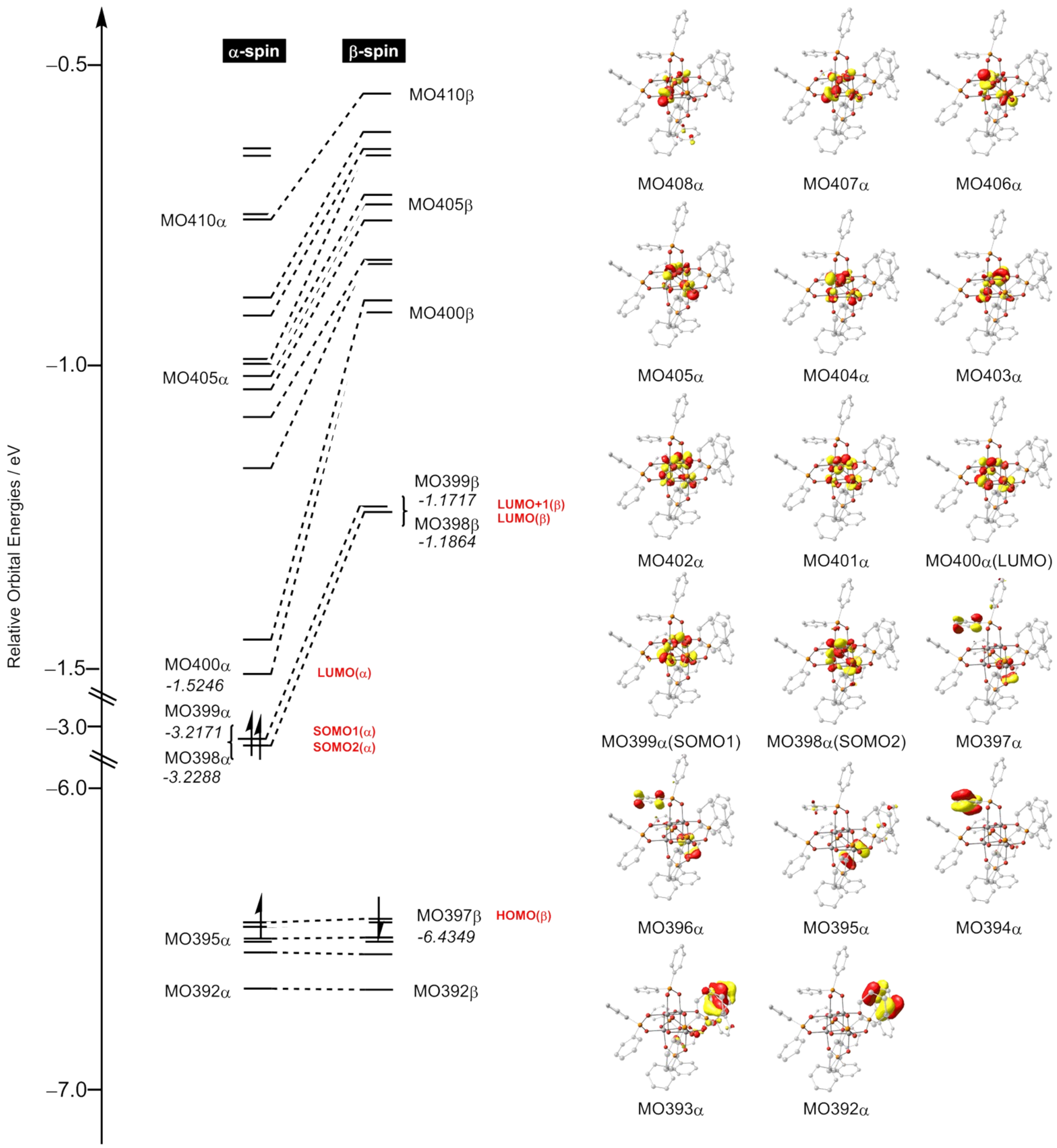

Figure S72. Canonical Kohn-Sham molecular orbital diagram (B3LYP/def2-TZVP(–f)/def2-SV(P)) of **7**.

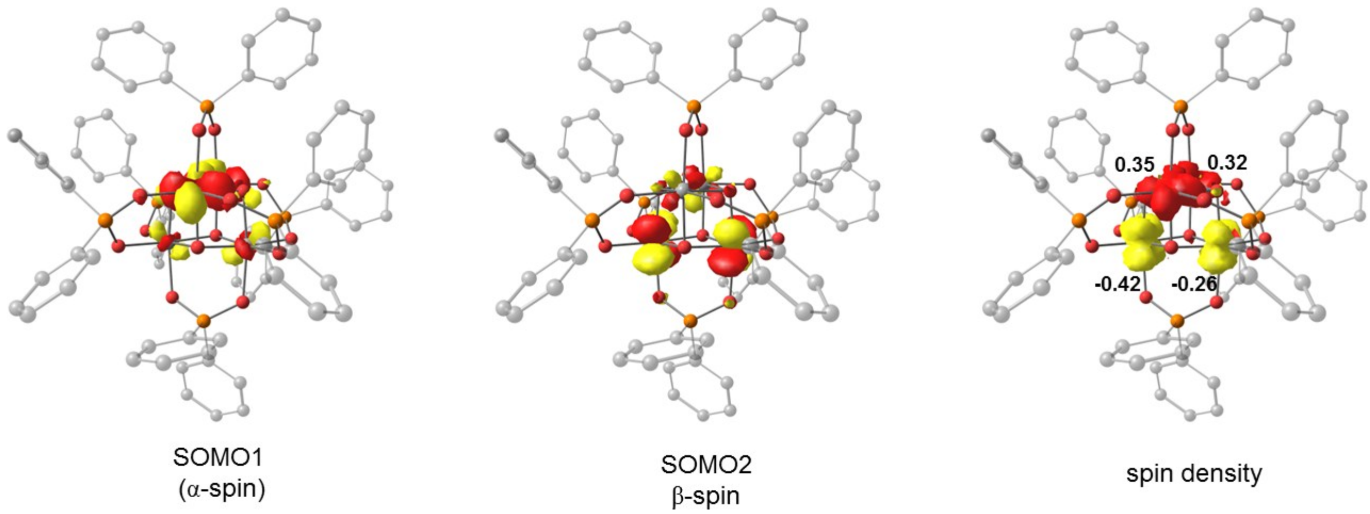

Figure S73. Singly-occupied α- and β-spin magnetic orbitals (from corresponding orbital transformation) for the broken-symmetry (M_s = 0) state of **7** (R = Ph). This state lies 0.06 eV above the triplet ground state, indicative of ferromagnetic coupling between the two spin centres. The orbitals shown correspond to antiferromagnetic coupling of the two unpaired electrons (isovalue = 0.05 au). The spin density plot is shown along with Loewdin atomic spin populations.

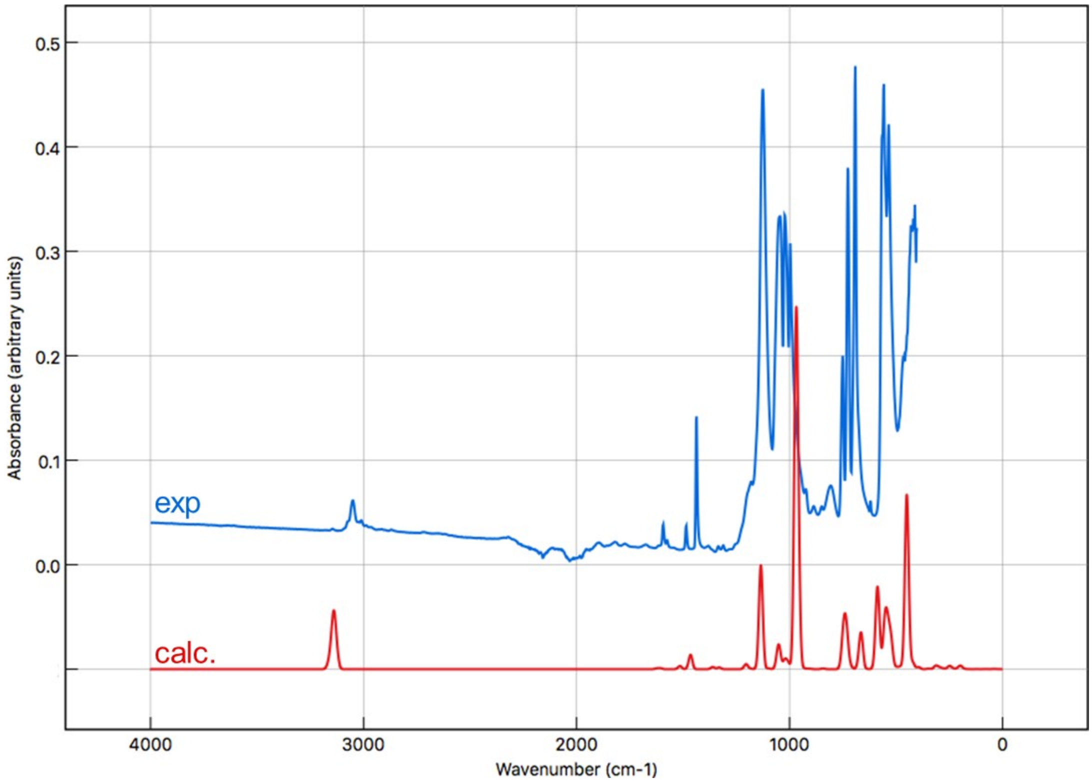

Figure S74. ATR-IR spectrum of bulk sample of **7** collected in an argon filled glovebox. Spectrum calculated by DFT (B97-3c) for **7** shown in red (scaled intensities). Note the spectrum collected for **7** is distinct from that of Ph_2PO_2H and although similar to that for **5** shows sharper signals indicative of a high symmetry structure.

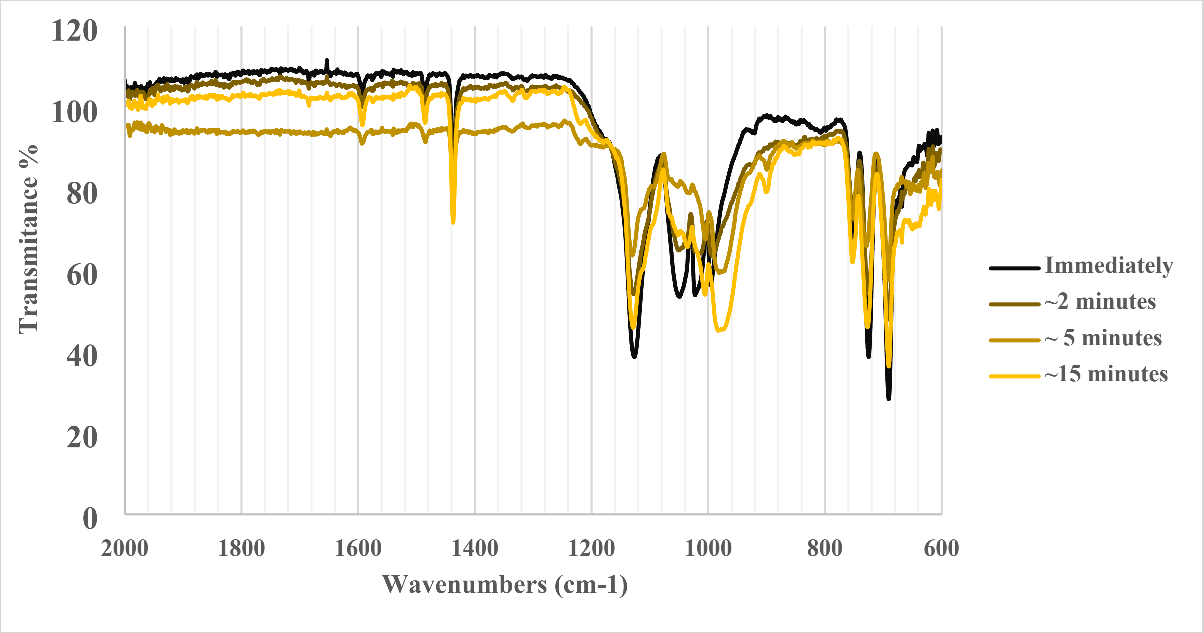

Figure S75. ATR-IR spectrum of a sample of **7** under air during oxidisation in air (grey to yellow).

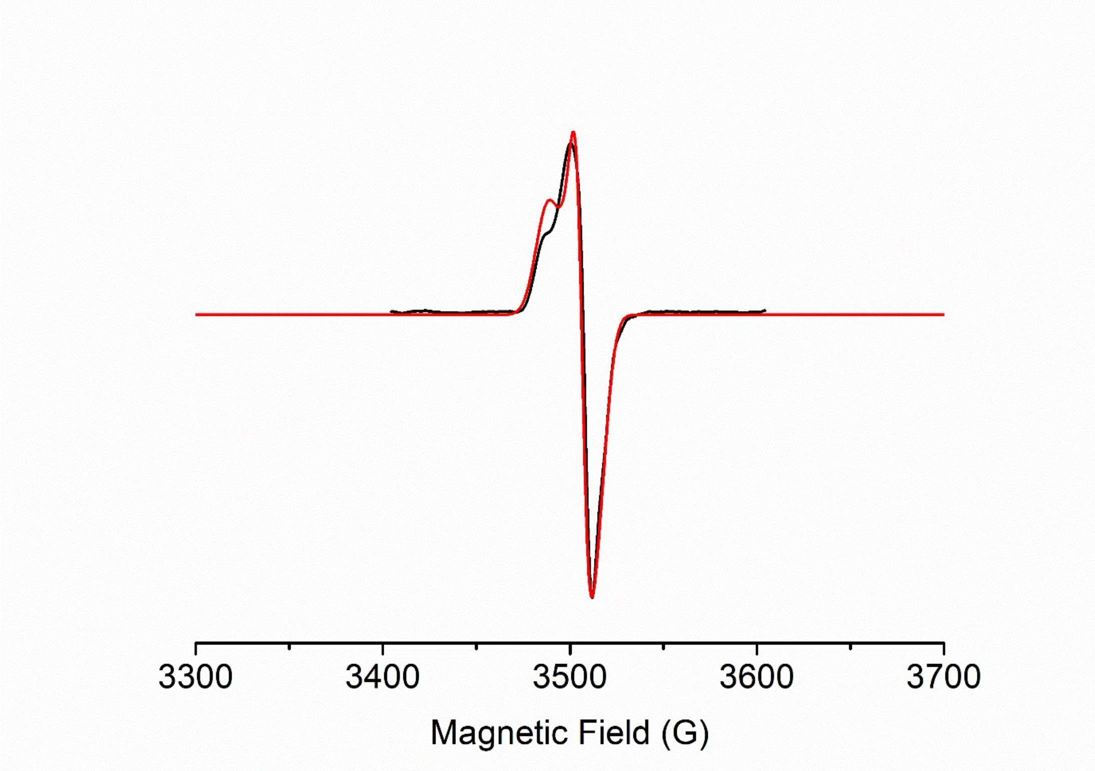

Figure S76. Experimental (black) and simulated (red) X-band EPR spectrum at room temperature of a powder of **7** exposed to air (oxidation with a colour change from black to yellow), modelled with S = ½ and g = 2.017; 2.006; 2.001

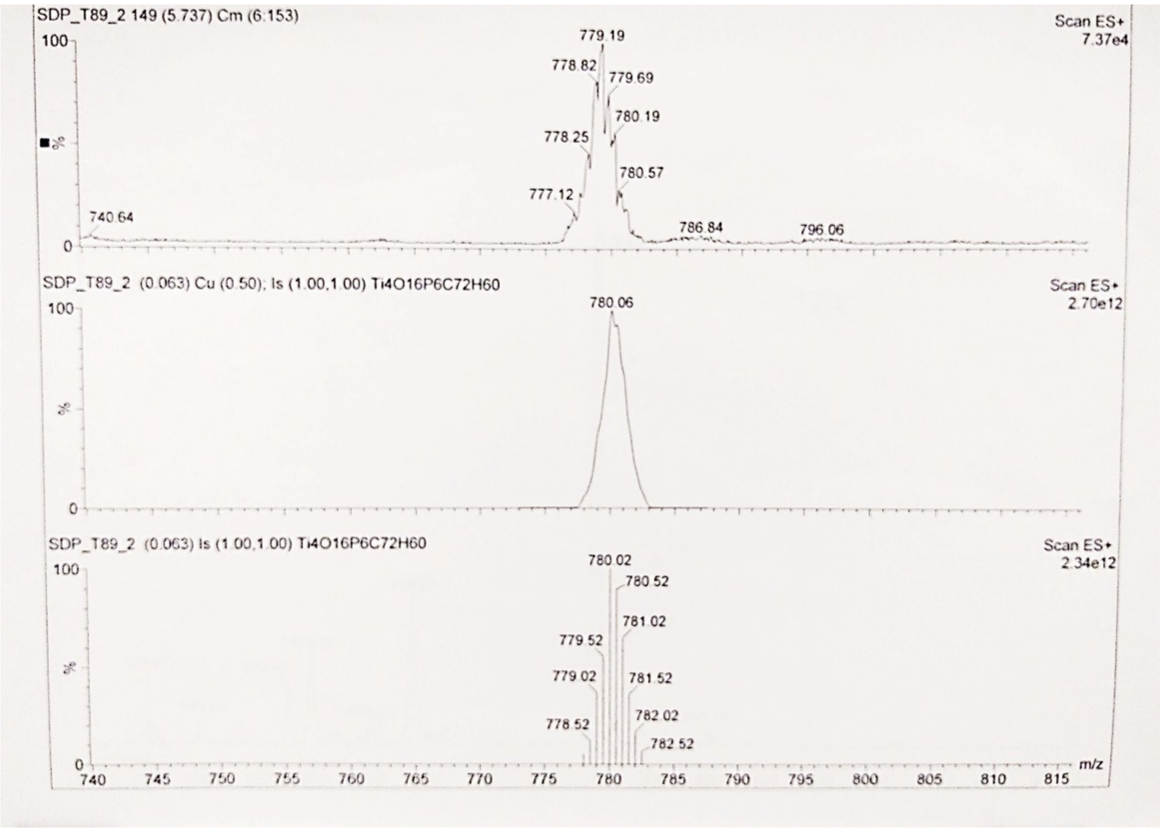

Figure S77. ESI Mass Spectrum of a solution of **7-ox** dissolved in CH_2Cl_2 (collected, above; predicted, middle and below): $[Ti_4O_4(Ph_2PO_2)_6]_4^{2+}$ found 779.2, predicted 779.0, N.B. this 2+ signal was not observed when a solution of **5** was injected into the spectrometer.

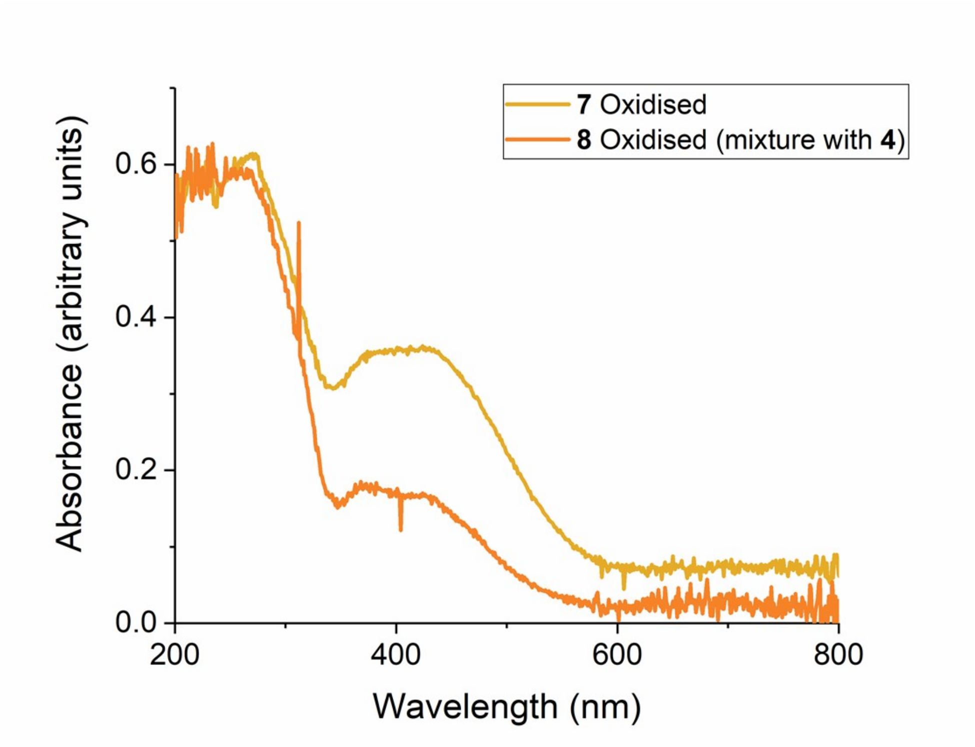

Figure S78. Diffuse reflectance UV-vis spectrum of a sample of **7** and **8** after oxidation under air (yellow colour) (N.B. sample of **8** was mixed with starting material **4**). An absorbance peaking at 412 nm attributed to superoxide/peroxide formation causing the yellow colouration.[34] The OMCT absorbance can be seen rising at higher energies (e.g. < 350 nm).

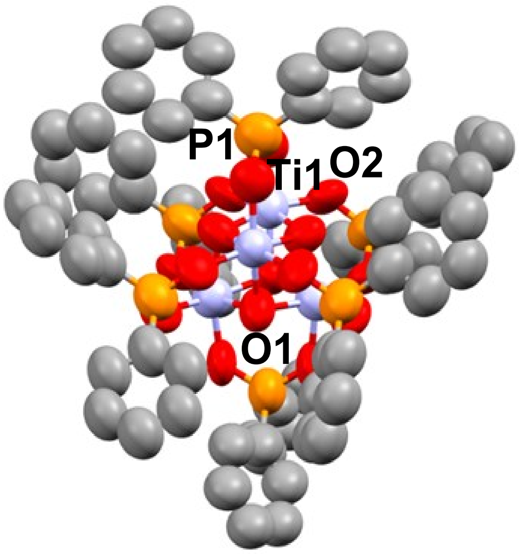

Figure S79. Solid state structure of a single crystal of **7** after oxidation in air (hydrogen atoms omitted for clarity). Ellipsoids displayed at 50% probability. Approximate bond lengths (Å) and angles (°) (*Caution: low quality data set not suitable for accurate bond length/analysis*) **7**: Ti1-O1, 1.963(13); Ti1-O2, 1.941(19); Ti1···Ti1’, 3.018(17); P1-O2, 1.545(17); Ti1-O1-Ti1’, 100.5(10), O2-P1-O2’ 115.7(15).

Characterisation and DFT calculations for 8, $[Ti_4O_4(Cy_2PO_2)_5(O^iPr)_2]$ and 8-ox $[Ti_4O_4(Cy_2PO_2)_5(O^iPr)_2][O_2]$

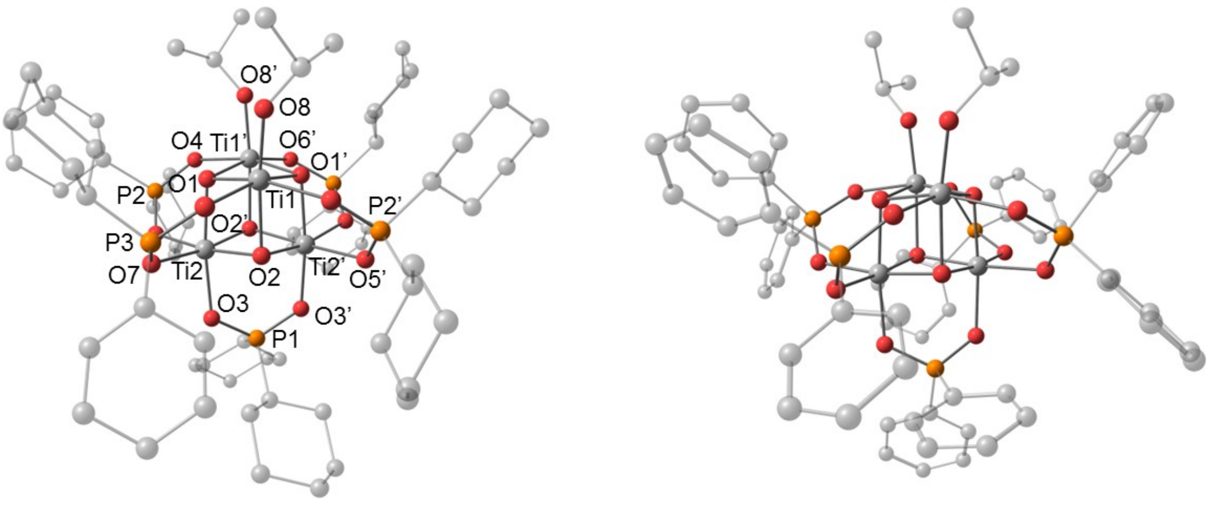

Figure S80. DFT-optimized geometries (B97-3c) of clusters **8** (left, R = Cy) and $\mathbf{8^{Ph}}$ (right, R = Ph) in their doublet (*S* = 1/2) ground states. Hydrogen atoms omitted for clarity.

Table S8. Key DFT-optimized bond parameters (Å, deg) for clusters **8** (R = Cy) and **8**^{Ph} (R = Ph).

	**7** (R = Cy)		**7**^{Ph} (R = Ph)
	S = 1/2	X-ray	*S* = 1/2
Ti1–O1 (oxo)	1.929	1.925(2)	1.940
Ti1–O1′ (oxo)	1.982	1.957(2)	1.986
Ti1–O2 (oxo)	2.069	2.056(2)	2.064
Ti2–O1 (oxo)	1.985	2.053(2)	1.965
Ti2–O2 (oxo)	1.924	1.932(19)	1.934
Ti2–O2′ (oxo)	1.907	1.953(2)	1.906
Ti1–O8 (iso-propylate)	1.802	1.795(3)	1.796
Ti–$OPOPh_2$	2.006–2.059	2.010(2)–2.032(2)	2.008–2.070
P–O	1.544–1.553	1.519(2)–1.528(2)	1.538–1.551
Ti1···Ti1′	2.917	2.947(12)	2.918
Ti2···T2′	2.806	2.911(12)	2.804
Ti1···T2	2.992	3.008(8)	2.981
Ti1···T2′	3.019	3.047(9)	3.020
Ti1–O1–Ti1′	96.36	98.76(9)	95.80
Ti2–O2–Ti2′	94.28	97.08(9)	94.30
O–P–O	114.94–115.95	114.48(14)–115.52(19)	116.00–117.77

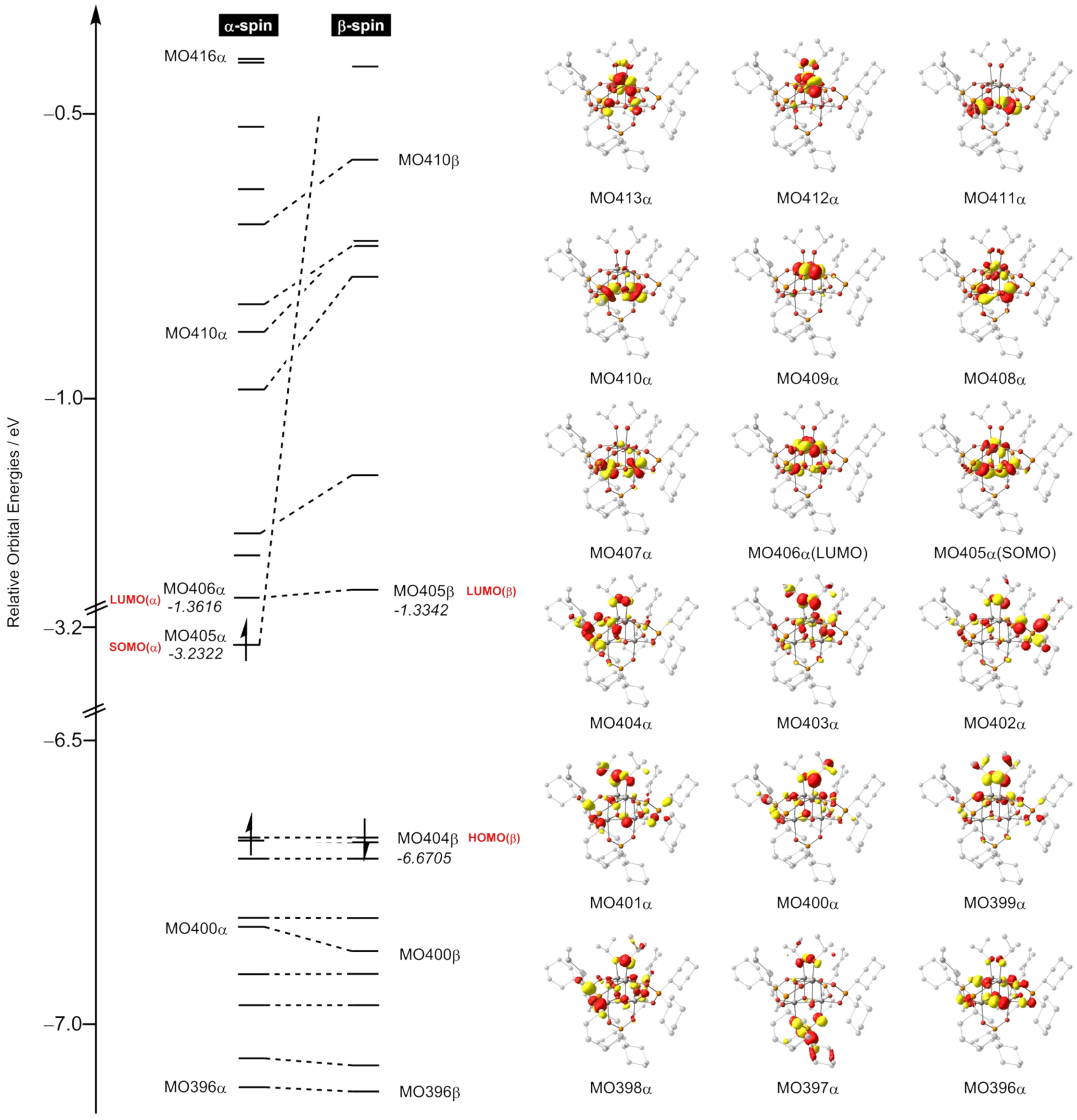

Figure S81. Canonical Kohn-Sham molecular orbital diagram (B3LYP/def2-TZVP(–f)/def2-SV(P)) of **8**.

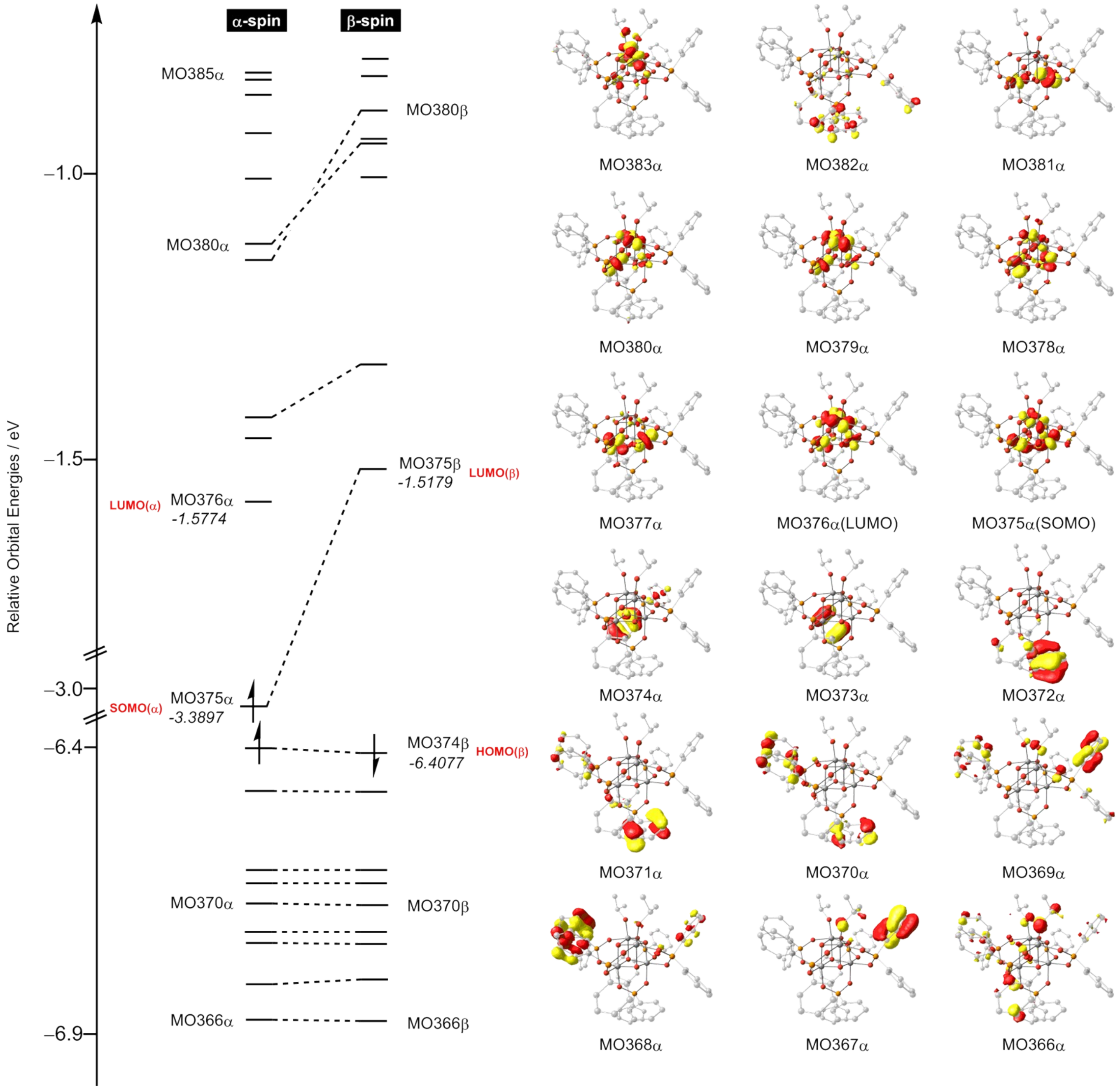

Figure S82. Canonical Kohn-Sham molecular orbital diagram (B3LYP/def2-TZVP(–f)/def2-SV(P)) of $\mathbf{8^{Ph}}$.

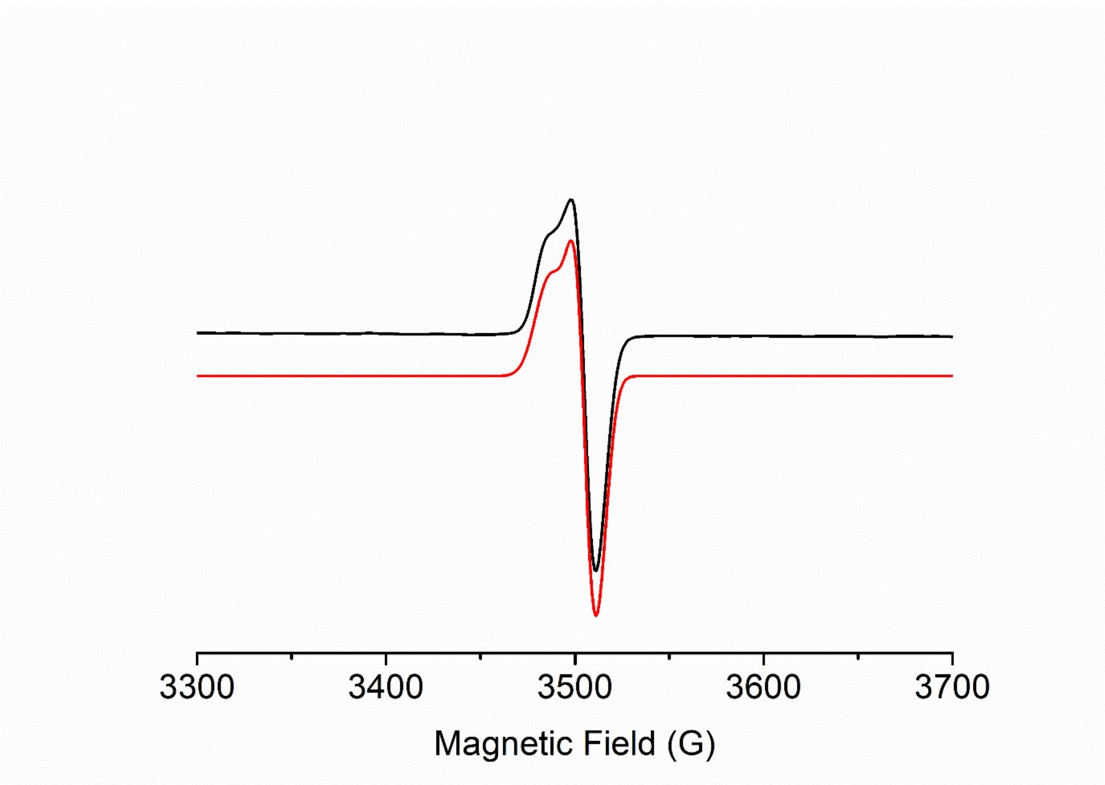

Figure S83. Experimental (black) and simulated (red) X-band EPR spectrum at room temperature of a powder of **8** exposed to air (oxidation with a colour change from blue to yellow), modelled with S = ½ and g = 2.0182; 2.007; 2.0023

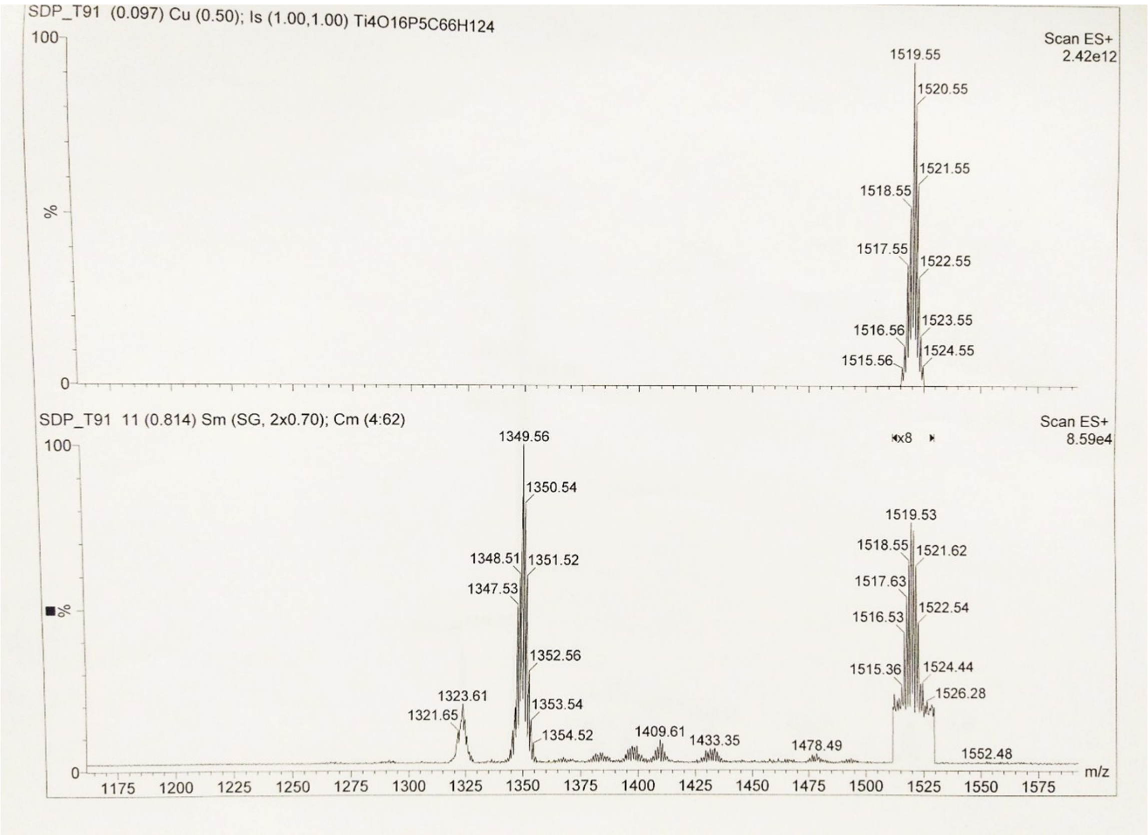

Figure S84. ESI Mass Spectrum of **8-ox** dissolved in CH_2Cl_2 (calculated above; collected below, with signal for [**8**]$^+$ amplified x8): $[Ti_4O_4(O^iPr)_2(Cy_2PO_2)_5]^+$ found 1519.5, predicted 1519.6. Major other signal (m/z = 1349.6) for $[Ti_4O_4(O^iPr)_3(Cy_2PO_2)_4]^+$ as seen for ESI-MS of **4**.

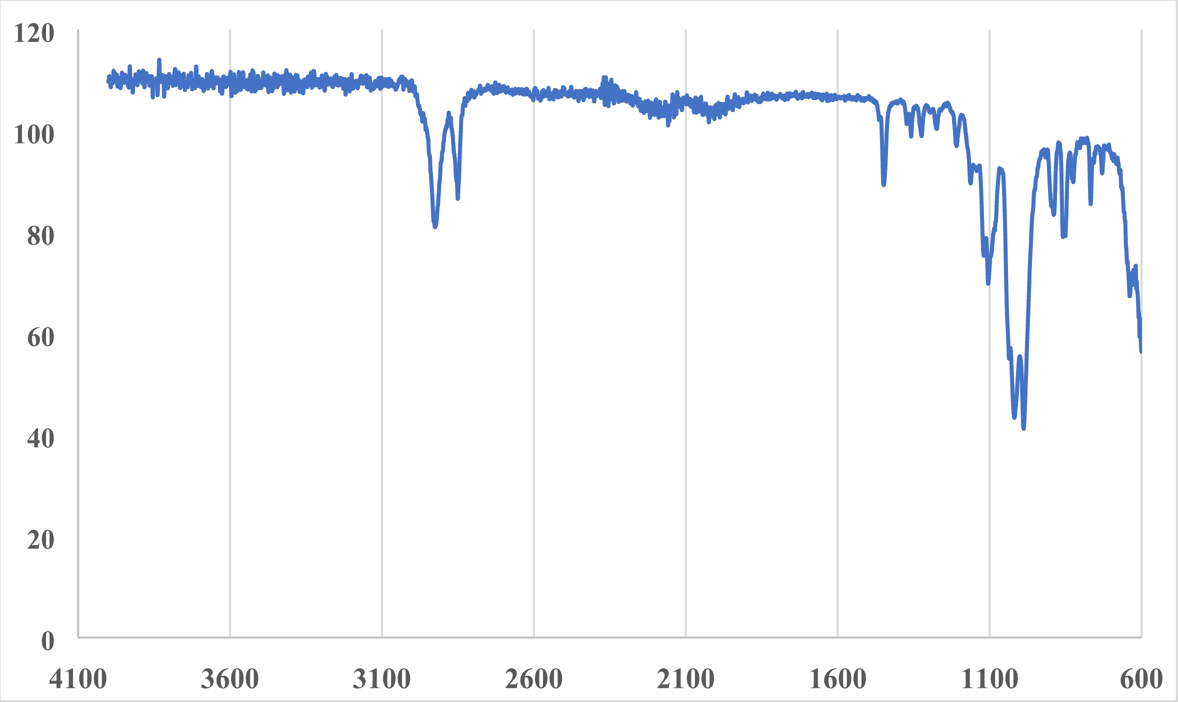

Figure S85. ATR-IR spectrum of a sample of **8** under air after oxidation (yellow). N.B. some **4** may also be present in the sample.

Crystallography information

Data were collected using an Enraf Nonius Kappa CCD diffractometer (at 180 K), a Bruker D8 VENTURE equipped with high-brilliance I µS Cu-Kα radiation (1.54178 Å) (at 180K), or at Beamline I19 of Diamond Light Source employing silicon double crystal monochromated synchrotron radiation (0.6889 Å) (at 100(2) K). Data integration and reduction were undertaken with SAINT and XPREP or with Xia2. Multi-scan empirical absorption corrections were applied to the data using SADABS[35] or the AIMLESS tool in the CCP4 suite. All structures were solved *ab initio* using Sir92[36] or Superflip[37] then refined with Crystals.[3] Crystallographic data have been deposited with the CCDC.

Several single crystal X-ray diffraction experiments were conducted on **7-ox**, which was a transparent yellow crystal (after single-crystal to single-crystal transformation from **7**), including at a synchrotron facility. The crystals diffracted with a clear pattern which became very weak at high angle, this may be due to micro-cracking during the single-crystal to single-crystal transformation.

Table S11. Crystallographic data. **5/6**, **5^{Bu4}** and **7-ox** are lower quality datasets and ae useful for connectivity only, and should not be used for accurate bond length of angle analysis.

Compound	**1**	**3**	**4**
CCDC No.	1902112	1902113	1902111
X-ray source	Mo Kα	Cu Kα	Cu Kα
Formula	C_{42} H_{86} O_{10} P_2 Ti_2	C_{81} H_{99} O_{17} P_5 Ti_3	C_{60} H_{116} O_{16} P_4 Ti_4
M	908.88	1643.23	1409.07
Crystal System	Triclinic	Triclinic	orthorhombic
Space group	P -1	P -1	F d d d
T [K]	180	180	180
a [Å]	13.6325(2)	12.4678(3)	15.2031(9)
b [Å]	13.7555(2)	17.6542(5)	21.8563(13)
c [Å]	14.7582(2)	20.1669(5)	43.312(3)
α [deg]	92.5897(7)	95.0910(17)	90
β [deg]	100.6998(7)	101.4601(17)	90
γ [deg]	93.1673(9)	103.3116(17)	90
V [Å^3]	2710.88(4)	4191.23(10)	14391.8(9)
Z	2	2	8
θ range [deg]	1.485 – 27.428	2.258 - 67.127	4.683 - 66.793
Reflns collected	37059	213647	21757
R int	0.056	0.161	0.037
No. of data/restr/param	12073/1156/577	13525/2167/1117	3178/324/274
R_1 [I>2σ(I)]	0.0560	0.0764	0.0566
wR_2 [all data]	0.1404	0.1651	0.1872
GoF	0.9816	0.9600	0.9833
Largest diff. pk and hole [eÅ^3]	−0.57 - 0.47	−0.98 - 0.98	−0.36 - 0.63

Compound	**5**.tol	**5** (73%) / **6** (27%)	**5^{Bu4}**
CCDC No.	1902115	1912034	1912035
X-ray source	Cu Kα	Cu Kα	Synchrotron
Formula	C_{67} H_{77} O_{16} P_4 Ti_4	$C_{67.55}$ $H_{75.45}$ $O_{15.73}$ $N_{0.27}$ P_4 Ti_4	C_{64} H_{76} O_{26} P_4 Ti_4 $.C_{3.5}H_4$
M	1452.82	1458.29	2936.74
Crystal System	monoclinic	monoclinic	Triclinic
Space group	P 2_1/c	P 2_1/c	P -1
T [K]	180	180	100(2)
a [Å]	12.5978(2)	12.6744(3)	13.2500(3)
b [Å]	16.2766(3)	16.2433(4)	16.5507(3)
c [Å]	33.9865(5)	33.7928(7)	33.5428(10)
α [deg]	90	90	96.740(2)
β [deg]	97.2246(8)	97.3936(12)	93.665(3)
γ [deg]	90	90	102.553(2)
V [Å^3]	6913.58(23)	6899.22(14)	7100.0(2)
Z	4	4	2
θ range [deg]	2.621 - 66.604	2.621 - 66.604	0.595 – 35.955

Reflns collected	50079	47361	44780
R int	0.066	0.055	0.053
No. of data/restr/param	12093/216/876	12116/1707/931	22022/3696/1847
R_1 [I>2σ(I)]	0.0392	0.0485	0.1100
wR_2 [all data]	0.1117	0.1214	0.3843
GoF	0.9093	0.9540	1.3078
Largest diff. pk and hole [eÅ^3]	-0.8 – 1.1	-0.96 – 1.33	-1.7 – 3.43

Compound	**8**	**7**	**7-ox** (and repeat)	
CCDC No.	1902116	1902114	1912033	
X-ray source	Synchrotron	Cu Kα	Cu Kα	Cu Kα
Formula	C_{72} H_{138} O_{16} P_5 Ti_4	C_{72} H_{60} O_{16} P_6 Ti_4	C_{72} H_{60} O_{16} P_6 Ti_4	C_{72} H_{60} O_{16} P_6 Ti_4
M	1606.34	1558.70	1558.70	1558.70
Crystal System	Monoclinic	Cubic	Cubic	Cubic
Space group	C 2/c	I -4 3 m	I -4 3 m	I -4 3 m
T [K]	100(2)	180	180	180
a [Å]	25.1958(4)	15.0884(5)	15.069(9)	15.088(5)
b [Å]	17.3647(3)	15.0884(5)	15.069(9)	15.088(5)
c [Å]	22.5546(3)	15.0884(5)	15.069(9)	15.088(5)
α [deg]	90	90	90	90
β [deg]	123.9560(8)	90	90	90
γ [deg]	90	90	90	90
V [Å^3]	8185.20(11)	3435.02(11)	3421.5176(13)	3434.7(10)
Z	8	2	2	2
θ range [deg]	1.469 – 36.082	4.143 - 66.875	4.149 - 58.538	4.144 - 66.064
Reflns collected	88383	6134	4136	3066
R int	0.070	0.059	0.214	0.134
No. of data/restr/param	8551/216/492	597/244/67	489/244/67	535/366/66
R_1 [I>2σ(I)]	0.0569	0.0414	0.1389	0.2564
wR_2 [all data]	0.1851	0.1193	0.3164	0.5735
GoF	0.8883	1.0237	0.9304	1.5829
Largest diff. pk and hole [eÅ^3]	-0.41 - 1.13	-0.38, 0.28	-0.85, 1.67	-2.38, 1.99

Supporting Note 1.

*Refinement of co-crystal **5/6***

Data was collected for deep blue crystals grown from an irradiated (LW-UV, ~10 h) solution of **5** in toluene with 30 equiv. pyridine, which had been concentrated under vacuum. The blue crystals diffracted well and the dataset was collected using a unit cell very similar to that of pure **5** (Table S11). Structural refinement was initiated using the atom positions from the refined model of **5**. Inspection of the Fourier difference map after refinement revealed areas of electron density around one O^iPr group (Figure S61). C atoms were placed in these positions and the occupancies refined such that occupancy (O^iPr) + occupancy new atoms = 1. It was clear that the new atoms linked in a hexagonal arrangement with two of the six sites overlapping closely with the O^iPr O and one of the C atoms. The model was amended to include the minor occupancy unit as a pyridine, with the closest atom to Ti as a N. Further refinement (without restraints) indicated ~27% occupancy of the pyridine unit which retained a sensible hexagonal arrangement. To ensure a symmetrical arrangement (considering the overlap of electron density with the major occupancy component) restraints were placed upon the pyridine group for the final stage of refinement. The other three O^iPr groups were also inspected to determine if further replacement with pyridine was possible, two of these three groups show disorder of the O^iPr group over two sites, which hinders inspection of the residual electron density map. It was not possible to locate any further minor occupancy components and so the data suggests a minor occupany unit of $[Ti(iv)_3Ti(iii)O_4(O^iPr)_3(O_2PPh_2)_4(pyridine)]$.

A photoirradiated and concentrated solution of **5** in neat THF solvent also led to the formation of blue crystals, in this case only **5·**THF could be identified from the structural data, suggesting that a small % of a photoreduced complex (e.g. $[Ti(iv)_{4-x}Ti(iii)_xO_4(O^iPr)_{4-x}(O_2PPh_2)_4(THF)_x]$, $\mathbf{6^{THF}}$), co-crystallises with **5** in a similar way to that of **6**.

Supporting Note 2.

*Alkoxide exchange of **5** with EtOH and photoreaction of products*

Adding EtOH to a d^8-toluene solution of **5** causes a variety of new ^{31}P NMR signals to form indicative of alkoxide exchange, with a series of $[Ti_4O_4(O_2PPh_2)_4(O^iPr)_x(OEt)_{(4-x)}]$ (x = 0-4) complexes forming. Irradiation (LW-UV) forms a blue solution with the formation of both acetone and acetaldehyde and consumption of 88% of all starting materials after 5 hours. Analysis of the ^1H NMR integrals suggests that ~16% of all Ti−OR units were converted to the oxidised products acetone/aldehyde with an approximate 7:3 ratio of acetaldehyde to acetone.

Supporting Note 3.

Photocatalysis

The irradiation of a sample of **5** with 400 equiv of ^iPrOH in d^8-toluene under air led to the slow catalytic formation of acetone (7 equiv. acetone after 20 hours irradiation), it should be noted that mixing of air within the NMR tube was not optimised in this simple setup.

Supporting Note 4

Investigating paramagnetic NMR spectroscopy signals

During photo-irradiation experiments attempts were made to observe paramagnetic complexes using in-situ NMR spectroscopy. Photo-irradiation of a solution of **5** in the presence of ^iPrOH led to new minor broadened ^{31}P NMR signals identified at 48 and 196 ppm (Fig. S86), significantly shifted

from the typical region for Ti(IV)-(O_2PPh_2) complexes (16-32 ppm). Similarly, photo-irradiation of a solution of **4** in the presence of ^{i}PrOH led to new broadened ^{31}P NMR signals located at 86 and 241 ppm (Fig. S87), after prolonged MW-UV irradiation (e.g. > 5h, N.B. the signal at 86 ppm appears to grow in at a later stage of photo-irradiation). These signals are similarly shifted downfield to the signals observed from photo-reduced **5**. In both cases associated paramagnetically shifted ^{1}H signals could not be located. The new signals disappeared upon exposure of the solution to air, but It is unclear if these minor signals correspond to paramagnetic compounds or other decomposition products. The integrals of the minor ^{31}P signals in both experiments do not fully compensate for the loss of starting material (e.g. <10% by ^{31}P NMR integral after 5 h photo-irradiation), suggesting that the majority of paramagnetic species are not located in the spectra. The ^{31}P NMR signal at 239 ppm was also observed after re-solvation of a blue precipitate of a mixture of **8** and **4** was re-dissolved in d^{8}-toluene (Fig. S88).

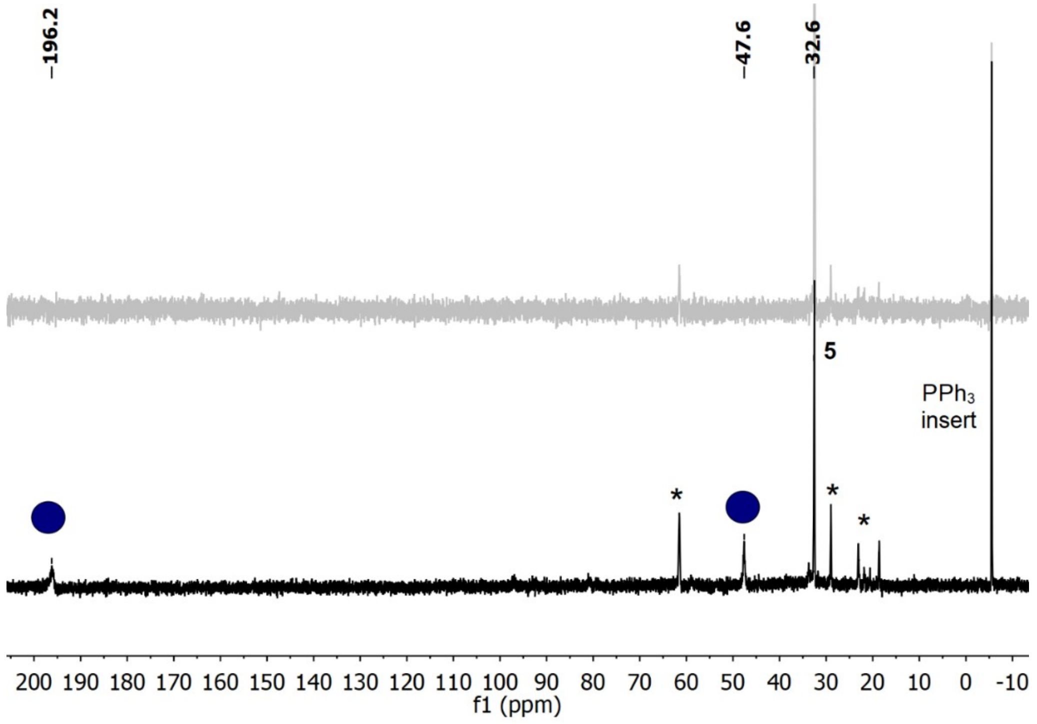

Figure S86. ^{31}P NMR spectrum (proton coupled) of **5** after 3 h irradiation with LW-UV light (grey spectrum before irradiation). Dark blue circles represent new signals that grow during photoreduction (* indicate minor impurities in capilliary containing PPh_3 and are not present when the capilliary is removed). N.B. due to slow precipitation of the products integration analysis is inaccurate in this experiment.

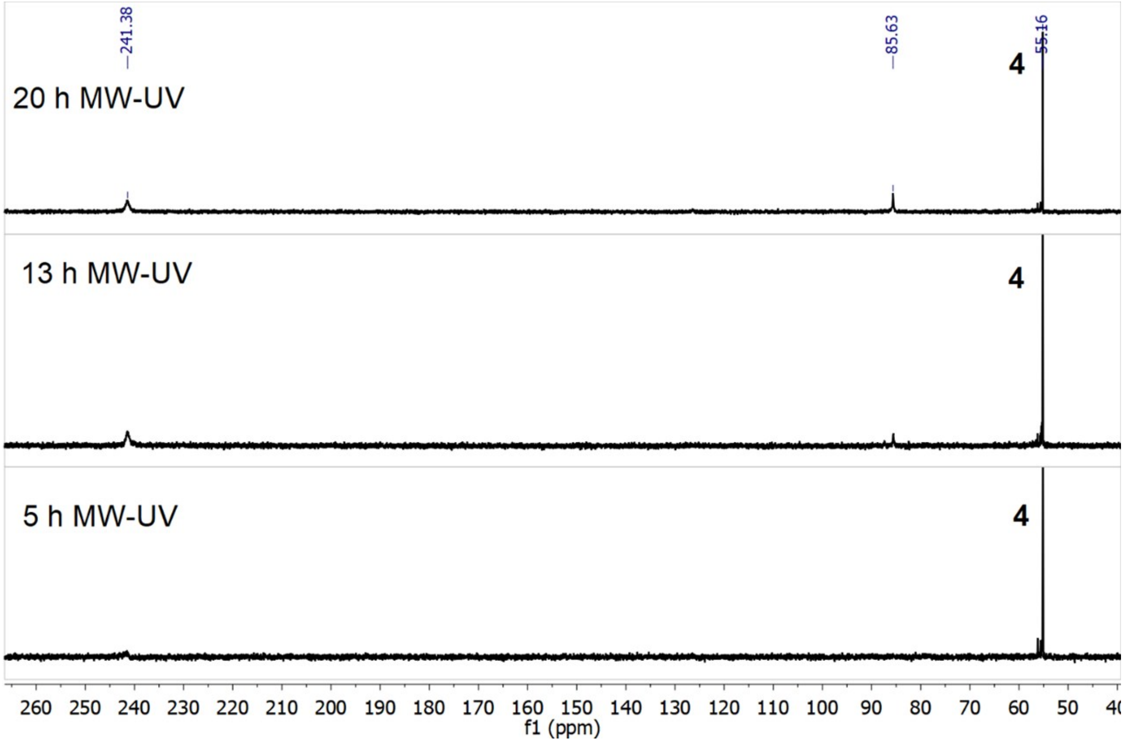

Figure S87. ^{31}P NMR spectrum (proton coupled) of **4** after 5, 13 and 20 h irradiation with MW-UV light.

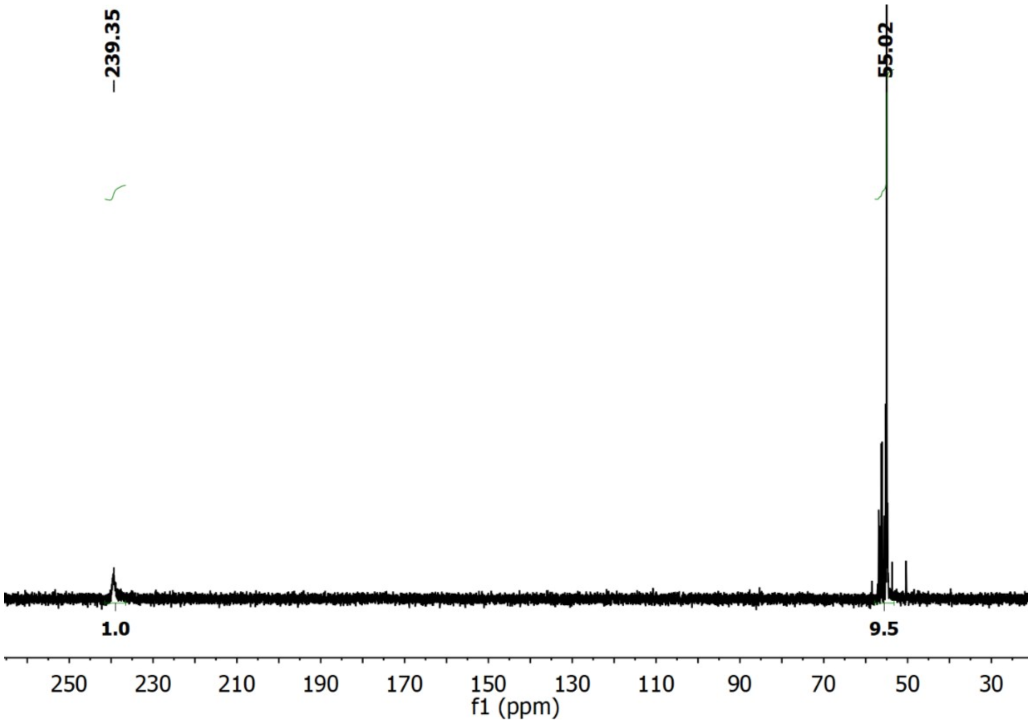

Figure S88. ^{31}P NMR spectrum (proton coupled) of blue powder containing **8** re-dissolved in d^8-toluene. Note the large quantity of starting material **4** (55 ppm) and diamagnetic decomposition products in the same region.

References

1. J. Tauc, A. Menth and D. L. Wood, *Phys. Rev. Lett.*, 1970, **25**, 749-752.
2. J. B. Benedict and P. Coppens, *J. Am. Chem. Soc.*, 2010, **132**, 2938-2944.
3. J. G. Brandenburg, C. Bannwarth, A. Hansen and S. Grimme, *J Chem Phys*, 2018, **148**, 064104.
4. F. Neese, *Wires Comput Mol Sci*, 2018, **8**, e1327.
5. A. D. Becke, *J Chem Phys*, 1997, **107**, 8554-8560.
6. S. Grimme, S. Ehrlich and L. Goerigk, *J Comput Chem*, 2011, **32**, 1456-1465.
7. S. Grimme, J. Antony, S. Ehrlich and H. Krieg, *J Chem Phys*, 2010, **132**, 154104.
8. A. D. Becke, *Phys Rev A*, 1988, **38**, 3098-3100.
9. C. T. Lee, W. T. Yang and R. G. Parr, *Phys Rev B*, 1988, **37**, 785-789.
10. A. D. Becke, *J Chem Phys*, 1993, **98**, 5648-5652.
11. A. Schafer, C. Huber and R. Ahlrichs, *J Chem Phys*, 1994, **100**, 5829-5835.
12. F. Weigend and R. Ahlrichs, *Phys Chem Chem Phys*, 2005, **7**, 3297-3305.
13. C. van Wullen, *J Chem Phys*, 1998, **109**, 392-399.
14. J. P. Perdew, *Phys Rev B*, 1986, **33**, 8822-8824.
15. L. Noodleman, *J Chem Phys*, 1981, **74**, 5737-5743.
16. F. Neese, F. Wennmohs, A. Hansen and U. Becker, *Chem Phys*, 2009, **356**, 98-109.
17. S. Kossmann and F. Neese, *Chem Phys Lett*, 2009, **481**, 240-243.
18. F. Weigend, *Phys Chem Chem Phys*, 2006, **8**, 1057-1065.
19. P. O. Lowdin, *J Chem Phys*, 1950, **18**, 365-375.
20. R. E. Stratmann, G. E. Scuseria and M. J. Frisch, *J Chem Phys*, 1998, **109**, 8218-8224.
21. T. Yanai, D. P. Tew and N. C. Handy, *Chem Phys Lett*, 2004, **393**, 51-57.
22. J. D. Chai and M. Head-Gordon, *J Chem Phys*, 2008, **128**.
23. Y. Zhao and D. G. Truhlar, *Theor Chem Acc*, 2008, **120**, 215-241.
24. J. P. Perdew, K. Burke and M. Ernzerhof, *Phys Rev Lett*, 1996, **77**, 3865-3868.
25. C. Adamo and V. Barone, *J Chem Phys*, 1999, **110**, 6158-6170.
26. A. V. Marenich, C. J. Cramer and D. G. Truhlar, *J Phys Chem B*, 2009, **113**, 6378-6396.
27. R. L. Martin, *J Chem Phys*, 2003, **118**, 4775-4777.
28. Y. Wang, Q. Liu, L.-l. Shi, C.-m. Cheng and L.-q. Chen, *Huaxue Shiji*, 2012, **34**, 785-786,806.
29. S. D. Pike, E. R. White, M. S. P. Shaffer and C. K. Williams, *Nat. Commun.*, 2016, **7**, 13008.
30. T. J. Boyle, R. P. Tyner, T. M. Alam, B. L. Scott, J. W. Ziller and B. G. Potter, *J. Am. Chem. Soc.*, 1999, **121**, 12104-12112.
31. J. Rohlicek and M. Husak, *J. Appl. Crystallogr.*, 2007, **40**, 600-601.
32. I. D. Brown and D. Altermatt, *Acta Crystallographica Section B*, 1985, **41**, 244-247.
33. A.S. Wills, VaList, Program available from www.ccp14.ac.uk
34. C. Kormann, D. W. Bahnemann and M. R. Hoffmann, *J. Phys. Chem.*, 1988, **92**, 5196-5201.
35. Bruker (2001). SADABS. Bruker AXS Inc., Madison, Wisconsin, USA.
36. A. Altomare, G. Cascarano, C. Giacovazzo, A. Guagliardi, M. C. Burla, G. Polidori and M. Camalli, *J. Appl. Crystallogr.*, 1994, **27**, 435.
37. L. Palatinus and G. Chapuis, *J. Appl. Crystallogr.*, 2007, **40**, 786-790.
